# Applications of Metal–Organic Frameworks and Their Derivatives in Electrochemical CO_2_ Reduction

**DOI:** 10.1007/s40820-023-01092-8

**Published:** 2023-04-30

**Authors:** Chengbo Li, Yuan Ji, Youpeng Wang, Chunxiao Liu, Zhaoyang Chen, Jialin Tang, Yawei Hong, Xu Li, Tingting Zheng, Qiu Jiang, Chuan Xia

**Affiliations:** 1 School of Materials and Energy, University of Electronic Science and Technology of China, Chengdu, 611731 People’s Republic of China; 2 Research Center for Carbon-Neutral Environmental and Energy Technology, University of Electronic Science and Technology of China, Chengdu, 611731 People’s Republic of China

**Keywords:** Metal–organic frameworks, Derivatives, Catalyst, CO_2_ reduction reaction, Electrocatalysis

## Abstract

Electrochemically reducing CO_2_ to more reduced chemical species is a promising way that not only enables the conversion of intermittent energy resources to stable fuels, but also helps to build a closed-loop anthropogenic carbon cycle. Among various electrocatalysts for electrochemical CO_2_ reduction, multifunctional metal–organic frameworks (MOFs) have been employed as highly efficient and selective heterogeneous electrocatalysts due to their ultrahigh porosity and topologically diverse structures. Up to now, great progress has been achieved in the design and synthesis of highly active and selective MOF-related catalysts for electrochemical CO_2_ reduction reaction (CO_2_RR), and their corresponding reaction mechanisms have been thoroughly studied. In this review, we summarize the recent progress of applying MOFs and their derivatives in CO_2_RR, with a focus on the design strategies for electrocatalysts and electrolyzers. We first discussed the reaction mechanisms for different CO_2_RR products and introduced the commonly applied electrolyzer configurations in the current CO_2_RR system. Then, an overview of several categories of products (CO, HCOOH, CH_4_, CH_3_OH, and multi-carbon chemicals) generated from MOFs or their derivatives via CO_2_RR was discussed. Finally, we offer some insights and perspectives for the future development of MOFs and their derivatives in electrochemical CO_2_ reduction. We aim to provide new insights into this field and further guide future research for large-scale applications.

## Highlights

- The electrochemical techniques utilizing metal-organic frameworks (MOFs)-based catalysts for converting CO_2_ into chemical species are discussed.
- The structure–activity relationship of MOF-based catalysts in electrocatalytic CO_2_ reduction reactions is thoroughly reviewed
- The challenges and opportunities of large-scale applications of MOF-based materials in electrochemical CO_2_ reduction reactions are discussed, and possible directions for the future development of MOFs and their derivatives are outlined.

## Graphical abstract

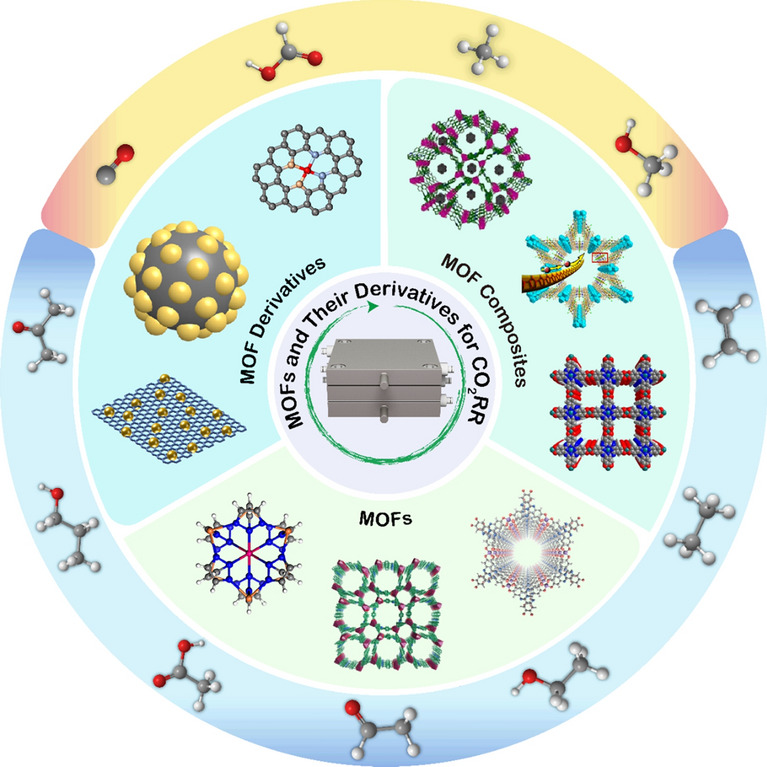

## Introduction

The overuse of fossil fuels has caused a rapid increase in atmospheric CO_2_ concentrations and disrupted the natural equilibrium of the carbon cycle, leading the global warming and subsequent consequences such as frequent storms, drought, and rising sea levels [1]. As climate change is getting worse, it is urgent to protect the ecological environment by developing advanced technologies to close the carbon loop. One potential approach is shifting current industries’ energy dependence from fossil fuels to renewable sources such as solar, wind, and thermal [2, 3]. However, unlike the controllable base-load energy in modern power grids, the inherent intermittency of renewable energy sources greatly limits their use or efficiency [4]. To solve the energy fluctuation problem, storing renewable energy in the chemicals form by converting CO_2_ into chemical feedstocks and fuels is a more promising way, which not only efficiently utilizes renewable energy but also reduces carbon emissions [5, 6].

Electrocatalytic reduction of CO_2_ to value-added chemicals using renewable electricity provides a practical solution to offset the extra carbon footprint [7–12]. However, the high activation barrier to break the symmetrical linear structure and stable C = O bonding (750 kJ mol^−1^) make the electrochemical process difficult [13–17]. Furthermore, the CO_2_ reduction reaction (CO_2_RR) process involves multi-electron/proton transfer processes and generates many different reaction intermediates, which are associated with a number of different reactions pathways and result in a large variety of products [18–24]. In recent years, efforts have been extensively dedicated to the design of catalysts with high efficiency and selectivity, resulting in noteworthy progress. Metal–organic frameworks (MOFs) are a class of porous coordination polymers consisting of metal ions/clusters coordinated to organic ligands, the open frameworks have high permanent porosity, high crystallinity, and long-range order [25–30]. The high surface area, tunable porosity, diversity in inorganic units and organic linkers make them attractive in catalytic reactions especially in the field of electrochemical CO_2_RR [31–37]: (1) The remarkably high porosity and surface area of MOFs facilitate the exposure of more active sites and boost rapid mass transport. (2) The electronic structure of MOFs can be precisely modulated via metal ion doping or functionalization of organic ligands, which influences the reaction pathways for CO_2_ electroreduction. (3) The varied organic linkers and abundant metal ions/clusters make MOFs attractive precursors or templates to fabricate numerous advanced MOF derivatives [such as metal/metal oxide nanoparticles, carbon materials and single-atom catalysts materials (SACs)] [38–47] as electrocatalysts for selective CO_2_RR.

There are various methods used for the synthesis of MOFs, including solvothermal, hydrothermal, microwave, and mechanochemical methods. The most common method is solvothermal synthesis, which involves the reaction of metal salts and organic ligands in a solvent at high temperature and pressure. The solvothermal method allows for precise control of the size, shape, and properties of the resulting MOFs. Hydrothermal synthesis is similar to solvothermal synthesis, but the reaction takes place in an aqueous solution instead of an organic solvent. This method is advantageous for the synthesis of water-stable MOFs. Microwave synthesis involves the use of microwave radiation to heat the reaction mixture and promote the formation of MOFs. This method is rapid and efficient and allows for the synthesis of MOFs in minutes rather than hours. Mechanochemical synthesis involves the use of mechanical force to induce chemical reactions between metal ions and ligands. This method is advantageous for the synthesis of MOFs with high thermal stability and is more environmentally friendly than traditional synthesis methods. Overall, the method chosen for the synthesis of MOFs for CO_2_RR involves a careful balance between catalytic activity, stability, and selectivity, with consideration given to the properties of the metal, ligand, and pore structure.

Primitive MOFs usually have drawbacks including low electrical conductivity, instability and inactive metal nodes due to the blockage of metal centers by organic ligands, which result in poor CO_2_RR performance. These limitations can be addressed by converting unstable MOFs into MOF derivatives, which can maintain the highly porous structure of the original MOF while also providing improved electrical conductivity and stability. MOF derivatives can be produced through electrochemical/chemistry reduction, surface modification, pyrolysis under specific conditions, and so on, which allow for precise control of material morphology, composition, surface area, and electronic structure of the metal nodes. For example, by controlling decomposition of MOFs under an inert atmosphere at high temperatures or under specific conditions can produce MOF-derived carbon materials or metal/metal oxide nanoparticles, respectively. Notably, MOF precursors with high thermal stability and metal loading can be thermally decomposed under an inert atmosphere to form SACs where isolated metal atoms are embedded in a carbon matrix, greatly enhancing the utilization efficiency of metal atoms. Overall, MOF derivatives largely expands the family of MOF materials and deliver superior performance to the pristine MOFs.

**Fig. 1:**
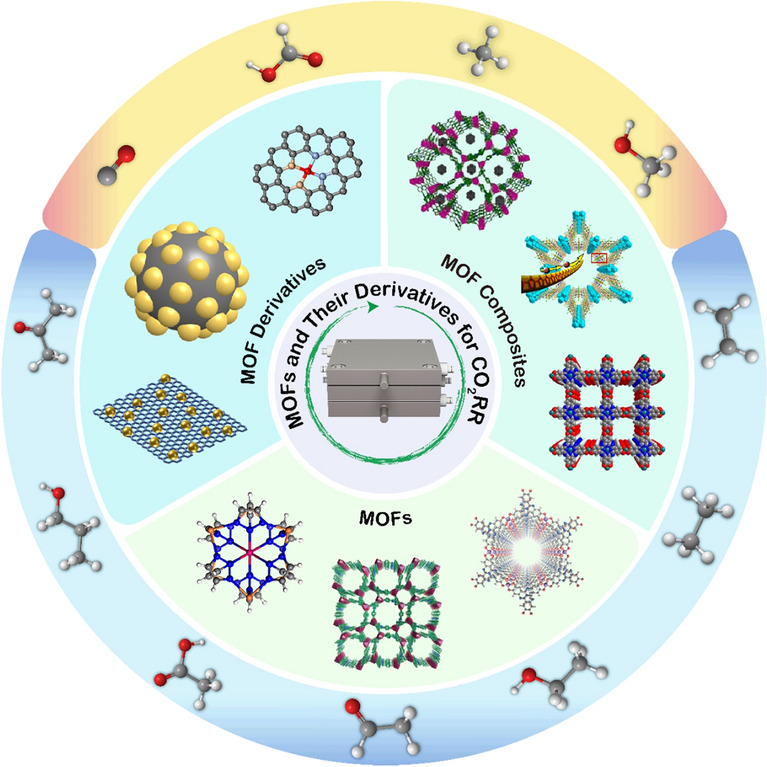
Schematic of MOFs and their derivatives for CO_2_RR

In the past years, MOF-related catalysts in electrochemical CO_2_RR systems have been thoroughly investigated, it is timely for us to take a systematic review to summarize the recent advances and pertinent challenges in this field (Fig. 1). The review starts with an introduction of the CO_2_RR reaction mechanisms at the molecular level, as well as a brief summary the electrolyzer structures. Then we discuss how to improve the selectivity and activity in electrochemical CO_2_RR toward different products from the perspective of materials design strategies such as pore structure modification, central metal atom substitution, and coordination environment adjustments. Finally, this review concludes with some of our insights about the research challenges and future directions, hoping to stimulate continuous innovations for advancing MOF-derived functional materials for electrochemical CO_2_RR.

## Mechanism and Electrolyzer Types of Electrocatalytic reduction of CO_2_

### Mechanism of Electrocatalytic Reduction of CO_2_

CO_2_ reduction was first reported by Royer in 1870 when they observed the formation of formic acid in an aqueous medium [48]. Since then, the mechanism of electrochemical CO_2_RR has been gradually discovered and a number of different possible reaction intermediates are identified, which essentially determine the reaction routes and final products [49, 50]. According to the number of carbon atoms, these products are typically divided into C_1_, C_2_, and C_3_ molecules, such as carbon monoxide (CO), formic acid (HCOOH), methanol (CH_3_OH), methane (CH_4_), ethylene (C_2_H_4_), acetic acid (CH_3_COOH), ethanol (CH_3_CH_2_OH), *n*-propanol (CH_3_CH_2_CH_2_OH), and acetone (CH_3_COCH_3_). Table 1 shows the corresponding half-reactions for different products as well as their standard reduction potentials versus reversible hydrogen electrodes (RHE) in both acidic and basic conditions [51, 52]. The large variety of possible CO_2_RR reaction pathways and their similar reduction potentials make the selective reduction to specific products a great challenge. Furthermore, in an aqueous electrolyte, the competition of hydrogen evolution reaction (HER) is a thorny issue that needs to be addressed as well.

**Table 1:** The half-reactions of electrochemical CO_2_RR as well as their standard reduction potentials versus reversible hydrogen electrode (RHE)

Products	Acid		Base	
	Equation	Potential (V)	Equation	Potential (V)
H_2_	2H^+^ + 2e^−^ → H_2_	0.000	2H_2_O + 2e^−^ → H_2_ + 2OH^−^	–0.828
CO	CO_2_ + 2H^+^ + 2e^−^ → CO + H_2_O	–0.104	CO_2_ + 2H_2_O + 2e^−^ → CO + 2OH^−^	–0.932
HCOOH	CO_2_ + 2H^+^ + 2e^−^ → HCOOH	–0.171	CO_2_ + H_2_O + 2e^−^ → HCOO^−^ + OH^−^	–0.639
CH_3_OH	CO_2_ + 6H^+^ + 6e^−^ → CH_3_OH + H_2_O	0.016	CO_2_ + 5H_2_O + 6e^−^ → CH_3_OH + 6OH^−^	–0.812
CH_4_	CO_2_ + 8H^+^ + 8e^−^ → CH_4_ + 2H_2_O	0.169	CO_2_ + 6H_2_O + 8e^−^ → CH_4_ + 8OH^−^	–0.659
C_2_H_4_	CO_2_ + 12H^+^ + 12e^−^ → C_2_H_4_ + 4H_2_O	0.085	2CO_2_ + 8H_2_O + 12e^−^ → C_2_H_4_ + 12OH^−^	–0.743
C_2_H_6_	2CO_2_ + 14H^+^ + 14e^−^ → C_2_H_6_ + 4H_2_O	0.144	2CO_2_ + 10H_2_O + 14e^−^ → C_2_H_6_ + 14OH^−^	–0.685
CH_3_CH_2_OH	2CO_2_ + 12H^+^ + 12e^−^ → CH_3_CH_2_OH + 3H_2_O	0.084	2CO_2_ + 9H_2_O + 12e^−^ → CH_3_CH_2_OH + 12OH^−^	–0.744
CH_3_CHO	2CO_2_ + 10H^+^ + 10e^−^ → CH_3_CHO + 3H_2_O	0.053	2CO_2_ + 7H_2_O + 10e^−^ → CH_3_CHO + 10OH^−^	–0.775
CH_3_COOH	2CO_2_ + 8H^+^ + 8e^−^ → CH_3_COOH + 2H_2_O	0.098	2CO_2_ + 5H_2_O + 8e^−^ → CH_3_COO^−^ + 7OH^−^	–0.653
CH_3_CH_2_CH_2_OH	3CO_2_ + 18H^+^ + 18e^−^ → CH_3_CH_2_CH_2_OH + 5H_2_O	0.095	3CO_2_ + 13H_2_O + 18e^−^ → CH_3_CH_2_CH_2_OH + 18OH^−^	–0.733
CH_3_COCH_3_	3CO_2_ + 16H^+^ + 16e^−^ → CH_3_COCH_3_ + 5H_2_O	0.102	3CO_2_ + 11H_2_O + 16e^−^ → CH_3_COCH_3_ + 16OH^−^	–0.726

#### C_1_ Pathways

**Fig. 2:**
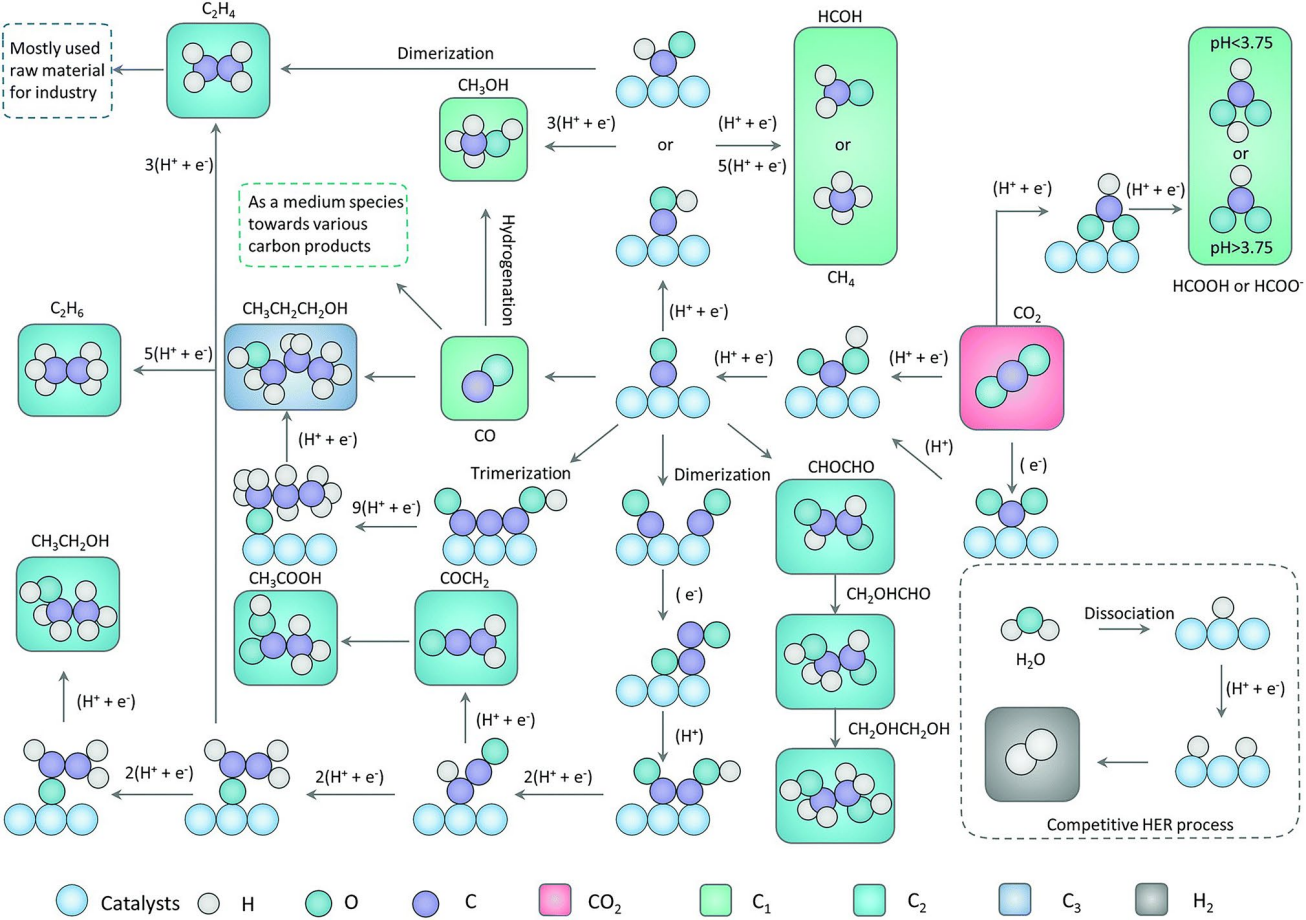
Possible mechanistic pathways of CO_2_ reduction to C_1_ and C_2_ products on catalysts. Reproduced with permission from Ref. [48]

In general, the first step of electrochemical CO_2_RR is to absorb CO_2_ molecules onto the catalyst surface and form *CO_2_^−^, which can further accept protons and/or electrons to form various intermediates that determine the final products [49, 53]. Figure 2 shows the possible mechanistic pathways of electrochemical CO_2_ reduction to C_1_ and C_2_ products, respectively. For C_1_ products especially two-electron products, such as CO and HCOOH (or HCOO^−^), the rate-limiting step is usually identified to be the formation process of *CO_2_^−^ [49, 52, 54]. The reaction pathway toward CO or HCOO^−^ is typically determined by the absorption configurations of the intermediate on the catalyst surface [55, 56]. Typically, when *CO_2_^−^ binds to the catalyst surface through two oxygen atoms and forms the *OCHO intermediate, then HCOOH is preferably formed by a proton-coupled electron transfer step. The reaction pathway toward CO is similar, a number of studies, which combines experimental and theoretical evidence, found that *COOH is a key descriptor for CO production [49, 52, 55, 57–60]. A carbon-bound *COOH intermediate is generated through the initial binding of CO_2_ to the surface of the catalyst. Subsequently, the concerted proton-electron transfer (CPET) steps are readily triggered by attacking the oxygen atoms and forming H_2_O, after dehydration, *CO intermediate can be easily desorbed as gaseous CO molecules when the binding energy between the catalyst and the intermediate is relatively low. In contrast, if the binding energy is strong, *CO can get further protonated to two intermediates *CHO or *COH. Then, the *CHO intermediate may be subsequently reduced via the protonation of its carbon atom, resulting in the formation of *CH_2_O (desorb as HCOH) and *CH_3_O. And *CH_3_O can be further reduced via the protonation of its carbon atom or oxygen atom to get CH_4_ or CH_3_OH, respectively. On the other hand, *COH also can be further reduced to *C and then hydrogenation to CH_4_ through the CPET steps [49, 52, 57].

#### C_2_ Pathways

*CO is a crucial intermediate for electrochemical CO_2_RR to get C_2_ product, and the reaction pathways of *CO intermediates via multiple CPET or other steps determine the final C_2_ products. Cu-based materials are the most efficient catalysts explored that show appreciable selectivity and faradaic efficiency for C_2_ products. On Cu(100) surface, *CO dimerization occurs prior to protonation at low overpotential [61], and is the rate-determining step for the formation of C_2_H_4_, CH_3_CH_2_OH, and CH_3_CH_2_CH_2_OH [13]. Besides the dimerization of *CO, the C–C bond could be formed by the coupling of other further protonated species. For example, on Cu(100) at high overpotential or on Cu(111) surface, *CO is protonated to *CHO before coupling with CO to form *COCHO, which is a key intermediate for getting C_2_ products [61, 62]. Another example is that *CH_2_ intermediate could be generated by the formation of C-H bonds from PECT steps, and it is a pivotal intermediate in the generation of C_2_H_4_ and CH_3_COOH [13, 63]. *CH_2_ can also be further protonated to *CH_3_ intermediate, which leads to C_2_H_6_ product via *CH_3_ dimerization. In addition, the reaction pathway for the formation of both C_2_H_4_ and CH_3_CH_2_OH productions involves CO insertion into *CH_2_, which is more commonly known as a Fischer–Tropsch-like step [64, 65]. Due to the abundance of intermediates and their protonation possibilities, there are many reaction pathways that form different C_2_ products, leading to uncontrollable product generation. Significantly, both experiments and density functional theory (DFT) calculations suggest that the rate-determining step in C–C coupling involves a decoupled proton-electron transfer, while whether the C–C coupling is an electrochemical step or a chemical step is still up for debate [66–69].

### Electrolyzer Types

**Fig. 3:**
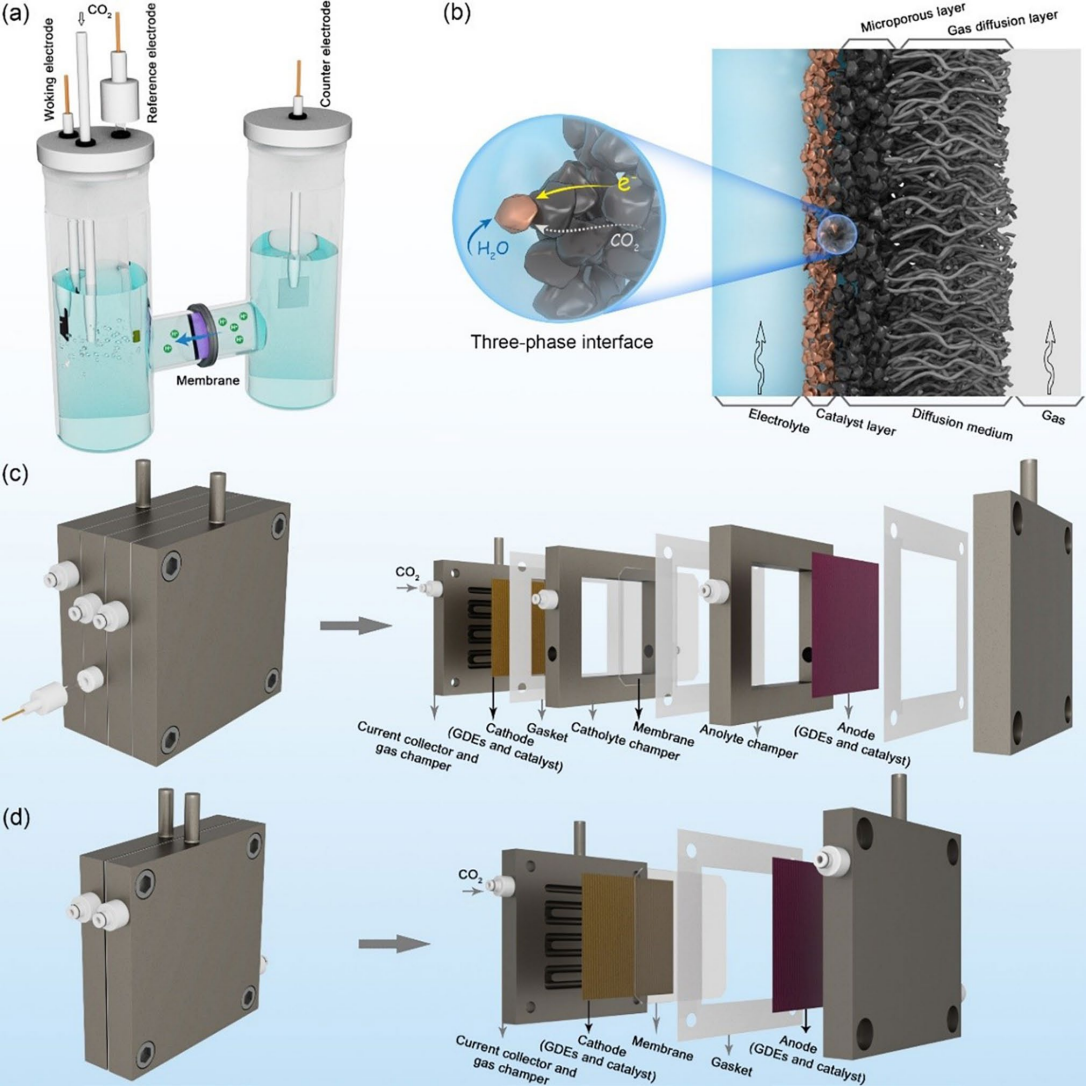
Schematic diagrams of the **a** H-types cell, **b** structure of GDEs, **c** flow cell, and **d** MEA cell

In the research of electrochemical CO_2_RR, there are mainly three types of electrolyzers: H-type cells, flow cells, and membrane electrode assembly (MEA) cells. Figure 3a shows a simplified configuration of a traditional H-type cell. The cell is composed of independent cathode and anode chambers, with the characteristics of easy operation, facile assembly, and low cost [13, 70, 71]. The two chambers are separated by an ion-exchange membrane, which allows the flow of ions while preventing the oxidation of the CO_2_ products by limiting their transport from cathode to anode. Catalysts are usually deposited or coated on conductive but inactive substrates (glassy carbon or carbon paper (CP)) and serve as working electrodes. In an H-type cell, CO_2_ molecules are commonly bubbled from the bottom of the aqueous electrolyte and saturate the electrolyte, then transferred to the interface of the working electrode where CO_2_RR takes place. However, the finite CO_2_ solubility in aqueous electrolyte (only 0.034 M under ambient conditions) results in limited CO_2_ reduction current densities (less than 100 mA cm^−2^) [57, 70]. Furthermore, the thick diffusion layer (> 50 μm) leads to poor mass transport between the catalyst surface and bulk electrolyte, resulting in a slow reaction rate [70, 72, 73]. Although H-type cells are hindered by such limitations for practical applications, it provides valuable information for evaluating the intrinsic catalytic performance of the catalysts.

To meet the industrial utilization of the electrochemical CO_2_RR, flow cells were developed so that CO_2_ can be efficiently delivered to the cathode continuously. Before introducing flow cells in more detail, it is necessary to learn about the structure and function of gas diffusion electrodes (GDEs). Figure 3b is a schematic of GDEs, which consists of a porous catalyst layer (CL) and a diffusion medium (DM) [74, 75]. The DM typically serves as the gas-permeable and electron-conductive substrate on which the CL is deposited. The substrate not only plays a role in determining the local electronic environment of the catalysts, but also influences the mass transfer of the reactants and products to and from the CL. Most DM consists of two parts, the gas diffusion layer (GDL) and the microporous layer (MPL). The GDL acts as a porous medium, which permits diffusion of both gaseous CO_2_ to the CL and gaseous products (H_2_, CO, CH_4_, C_2_H_4_) away from the CL surface [76, 77]. The unique structure of GDEs forms a gas–liquid-solid three-phase interface where the electrochemical CO_2_RR occurs without solubility limitations [78, 79]. To stabilize the triple-phase interface, MPL composed of carbon black nanoparticles is commonly treated with hydrophobic polytetrafluoroethylene to prevent flooding of electrolytes into GDEs, resulting in efficient mass transport for gaseous CO_2_.

In flow cells, GDEs are often used for better control of the three-phase interface where the gaseous CO_2_ feedstock can be directly reduced without mass diffusion limitation. Typical membranes in flow cells include the cation exchange membrane (CEM), anion exchange membrane (AEM), or a bipolar membrane (BPM), the type of membrane affects the applicable electrolyte conditions and the ion transport kinetics. As shown in Fig. 3c, a polymer electrolyte membrane is sandwiched between two electrolyte channels, dividing the cell into two parts: the anodic and cathodic compartments. Both cathode and anode electrolytes are continuously flowed through electrolyte channels by a pump. The anode side typically carries out a complementary oxidation reaction, which is most commonly water oxidation, typically using IrO_x_ nanoparticles on Ti mesh/GDEs [80–82]. In the cathode chamber, a GDE sits at the interface of the inbound CO_2_ and the flowing aqueous electrolyte, which largely improves the mass transport of gas–liquid interface, making it possible to conduct electrochemical CO_2_RR at industrial-scale current densities [70, 83, 84].

The MEA cell is another emerging class of electrolyzers that has been commonly applied to electrochemical CO_2_RR. As shown in Fig. 3d, by removing the flowing electrolyte channel between the GDE and membrane, it directly combines GDEs and ion exchange membrane into one unit. Benefiting from the uniqueness of this configuration, the MEA cell could significantly decrease the distance between the cathode and anode and thus boot the mass/electron transfer, resulting in high energy efficiency [85–88]. In addition, the removal of the flowing liquid electrolyte could relieve GDE flooding and reduce contamination of the cathode catalyst from impurities in electrolyte [89, 90].

## Using MOFs and their Derivatives as Catalysts for CO_2_ Electroreduction to Value-Added Chemicals

MOFs are porous crystalline materials synthesized from the coordination bonds of metal ions/clusters and organic ligands, and they possess open frameworks with tunable porous properties. In 2012, a copper rubeanate MOF was first reported as a catalyst for electrochemical CO_2_RR and was shown to effectively convert CO_2_ into HCOOH in 0.5 M KHCO_3_ solution, with an HCOOH selectivity above 98% [91]. Subsequently, Senthil Kumar et al. reported the generation of oxalic acid from CO_2_RR using Cu_3_(BTC)_2_ (BTC = 1,3,5-ben-zenetricarboxylicacid) catalyst in N,N-dimethylformamide, and a high Faradic efficiency (FE) of 51% [92] was achieved. Since then, MOF-related catalysts have received significant attention in electrochemical CO_2_RR and become a rapidly growing research area. Some of these materials have demonstrated obvious activity, selectivity, and stability for electrochemical CO_2_RR in various electrolytes. C_1_ chemicals, such as CO, HCOOH, CH_4_, and CH_3_OH, are generally the main products in current MOF-catalyzed CO_2_RR systems, whereas the desirable C_2_ products are relatively rare. Thus, this paper mainly focuses on the C_1_ products produced from MOF-related materials and discusses strategies to enhance their electrocatalytic performance, while state-of-art MOF-related materials for catalyzing CO_2_ to C_2+_ are also briefly summarized.

### Carbon Monoxide

CO is a crucial building block for the large-scale production of commodity and specialty chemicals [93]. Among all the products from electrochemical CO_2_RR, CO is one of the most economically viable products and has a high ratio of molecular weight per electron [94, 95]. As mentioned above, an ideal catalyst that selectively catalyzes the electrochemical reduction of CO_2_ into CO should possess not only strong adsorption energies of *COOH intermediate but also weak adsorption energies of *CO. However, the binding energies of *COOH and *CO are generally proportionally related and follow the scaling relations, it is hard to alter the reaction pathways individually. Fortunately, the linear relationship can be broken by regulating the intrinsic physical/chemical properties and electrochemical microenvironments of the catalytic materials. Owing to their tunable chemical properties, MOF-related materials are perfect candidates for CO_2_RR because of their tunable structure and compositions. MOFs also have the advantage of well-defined single-atom sites; this is helpful to elucidate the surface dynamic changes and chemical adsorption of reaction intermediates. So far, a number of MOF-related materials have been extensively explored for the reduction of CO_2_ to CO (Table 2), we will discuss their structure–activity relationships from the perspective of morphology, conductivity, and the coordination environment of metal center.

**Table 2:** Representative MOF-related catalysts for the electrochemical reduction of CO_2_ to CO

Catalyst	MOF precursor	Electrolyte	FE (%)	Potential (V *vs.* RHE)	Refs.
Al_2_(OH)_2_TCPP-Co	–	0.5 M KHCO_3_	76	−0.7	[96]
PCN-222(Fe)	–	0.5 M KHCO_3_	91	−0.6	[97]
NiPc-Ni(NH)_4_	–	0.5 M KHCO_3_	96.4	−0.7	[98]
NiPc-NiO_4_	–	0.5 M KHCO_3_	98.4	−0.85	[99]
Cu-THQ	–	1 M C_5_H_14_ClNO + 1 M KOH	91	−0.45	[100]
PcCu-O_8_-Zn	–	0.1 M KHCO_3_	88	−0.7	[101]
ZIF-A-LD	–	0.1 M KHCO_3_	90.6	−1.1	[102]
MNU-15	–	0.5 M KHCO_3_	99.2	−0.6	[103]
CALF20	–	1 M KOH	94	−0.97	[104]
CoCp_2_@MOF-545-Co	MOF-545-Co	0.5 M KHCO_3_	97	−0.7	[105]
PPy@MOF-545-Co	MOF-545-Co	0.5 M KHCO_3_	98	−0.8	[106]
Ni_SA_-N_2_-C	MgNi-MOF-74	0.5 M KHCO_3_	98	−0.8	[107]
Ni–N_4_/C–NH_2_	Ni-ZIF-8	0.5 M KHCO_3_	96.2	−1.0	[108]
CoPc©Fe–N-C	Fe-ZIF-8	0.5 M KOH	93	−0.84	[109]
STPyP-Co	–	0.5 M KHCO_3_	96	−0.62	[110]
Zn-TCPP(Co)-MOF	–	0.5 M CsHCO_3_	86.2	−0.7	[111]
Cu-MOFs	–	0.1 M KHCO_3_	77.5	−0.89	[112]
TPY-MOL-CoPP	–	0.1 M NaHCO_3_	92.2	−0.86	[113]

#### Pristine MOFs

**Fig. 4:**
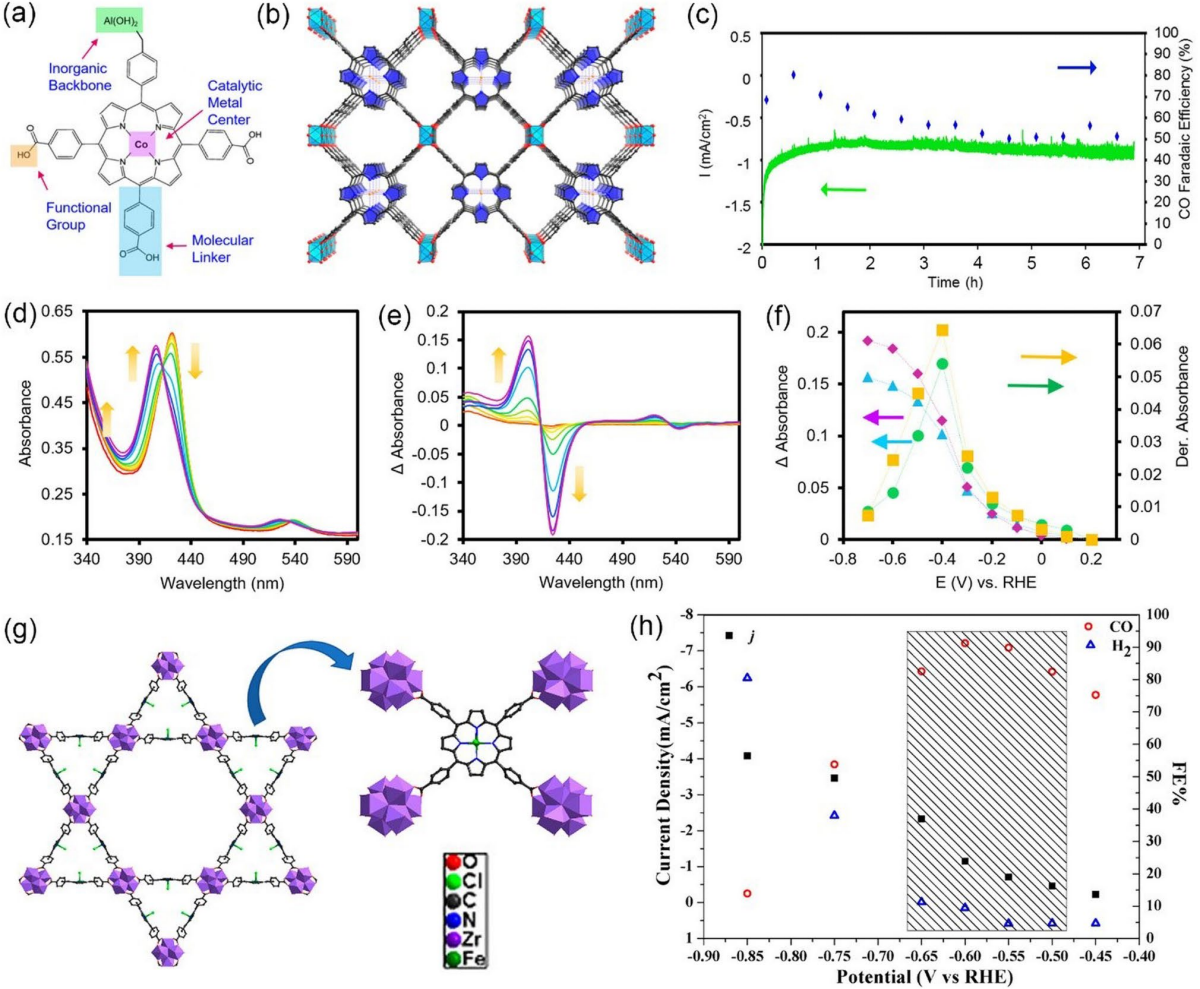
**a** MOF catalyst allows for the modulation of metal centers, molecular linkers, and functional groups at the molecular level. **b** Organic building units, in the form of cobalt-metallated TCPP, are assembled into a 3D MOF, Al_2_(OH)_2_TCPP-Co with variable inorganic building blocks. **c** Stability of the MOF catalyst is evaluated through chronoamperometric measurements in combination with faradaic efficiency measurements. **d** In situ spectroelectrochemical analysis reveals the oxidation state of the cobalt catalytic unit of the MOF under reaction conditions. Upon varying the voltage from 0.2 to -0.7 V vs RHE, the Co(II) Soret band decreases at 422 nm and is accompanied by a C5o(I) Soret band increase at 408 nm. This change is quantified and plotted **e** to elucidate a formal redox potential of the Co center, which is deemed to be at the peak of the first derivative **f** of the Co(II) bleach and Co(I) enhancement. Reproduced with permission from Ref. [96]. **g** 3D crystal structure of PCN-222(Fe). **h** Steady-state current density and the selectivity for each gas product in a potential range from -0.45 to -0.85 V vs. RHE. Reproduced with permission from Ref. [97]

Morphology and size are two important characteristics of metal–organic framework (MOF) materials that influence their properties and performance in various applications. In the case of MOFs, the morphology can vary from a crystalline powder to a dense film or even an ordered nanostructured material. The morphology of MOFs can be influenced by several factors such as the synthesis method, the precursor choice, and the reaction conditions. On the other hand, the dimensions of the MOF particles or the size of the pores within the MOF structure determines their adsorption properties. Controlling the morphology and size of MOF material not only maximizes the active sites but also balances both charge and mass transport, resulting in high catalytic activity. For example, Kornienko et al. [96] fabricated a porous thin film of Al_2_(OH)_2_TCPP-Co(TCPP-H_2_ = 4,4′,4″,4‴-(porphyrin-5,10,15,20-tetrayl)tetrabenzoate) MOF and employed it as an electrocatalyst for the efficient and selective reduction of CO_2_ to CO in aqueous electrolytes, as early as 2015. By optimizing the catalytic linker unit (Fig. 4a) and assembling a porous thin film MOF (Fig. 4b) on a conductive substrate, a three-dimensional (3D) porous working electrode was successfully prepared. As shown in Fig. 4c, when tested in CO_2_-saturated KHCO_3_ electrolyte for continuous electrolysis, Al_2_(OH)_2_TCPP-Co exhibited good catalytic activity and stability, with a maximum CO FE of over 76% and good stability over 7 h at −0.7 V *vs.* RHE. The cobalt centers are electrically linked to the electrode and are reduced to the catalytically active Co(I) state (Fig. 4d–f). And the rate-limiting step might be either a CO_2_ molecule adsorbing onto a Co(I) porphyrin coupled with a one-electron reduction or a one-electron reduction of a Co(I)-CO_2_ adduct. Thanks to the nanoscopic MOF morphology and thickness, the number of active sites is maximized and both charge and mass transported could be simultaneously balanced, thus improving the electrocatalytic performance. Moreover, Dong et al. [97] also use the same ligand TCPP as a building block to synthesize a 3D highly stable porphyrin-based MOF of PCN-222(Fe) (Zr_6_O_8_(OH)_4_(H_2_O)_4_][(TCPP-Fe-(III)-Cl)_2_). As shown in Fig. 4g, it consists of Zr_6_ clusters and square planar Fe-TCPP ligands, resulting in a star-shaped network. Thanks to the micromorphology, PCN-222(Fe) extensively exposes the porphyrin active sites, which have a substantial catalytic effect on the electrochemical reduction of CO_2_. Furthermore, the permanent porosity of PCN-222(Fe) conferred a promising CO_2_ adsorption ability, which is also advantageous in CO_2_ electrochemical reduction since the reduction kinetics are closely related to the CO_2_ concentration. When tested in CO_2_-saturated 0.5 M KHCO_3_ aqueous solution at various steady-state potentials from -0.45 to -0.85 V *vs.* RHE, PCN-222(Fe)/C (PCN-222(Fe) mixed with carbon black) showed excellent catalytic selectivity and activity toward CO. As shown in Fig. 4h, the product distribution in the gas phase was dependent on the applied potentials, and the maximum FE of CO occurs at -0.60 V *vs.* RHE (91%). In the low overpotential region, the Tafel slope of PCN-222(Fe)/C is 188 mV dec^−1^, which indicates that a one-electron reduction of CO_2_ to CO_2_^−^ radical is a probable rate-limiting step. PCN-222(Fe)/C also showed high structural stability after continuous electrolysis for 10 h, however, the low CO_2_-to-CO current density (< 10 mA cm^−2^) limited its large-scale industrial applications.

**Fig. 5:**
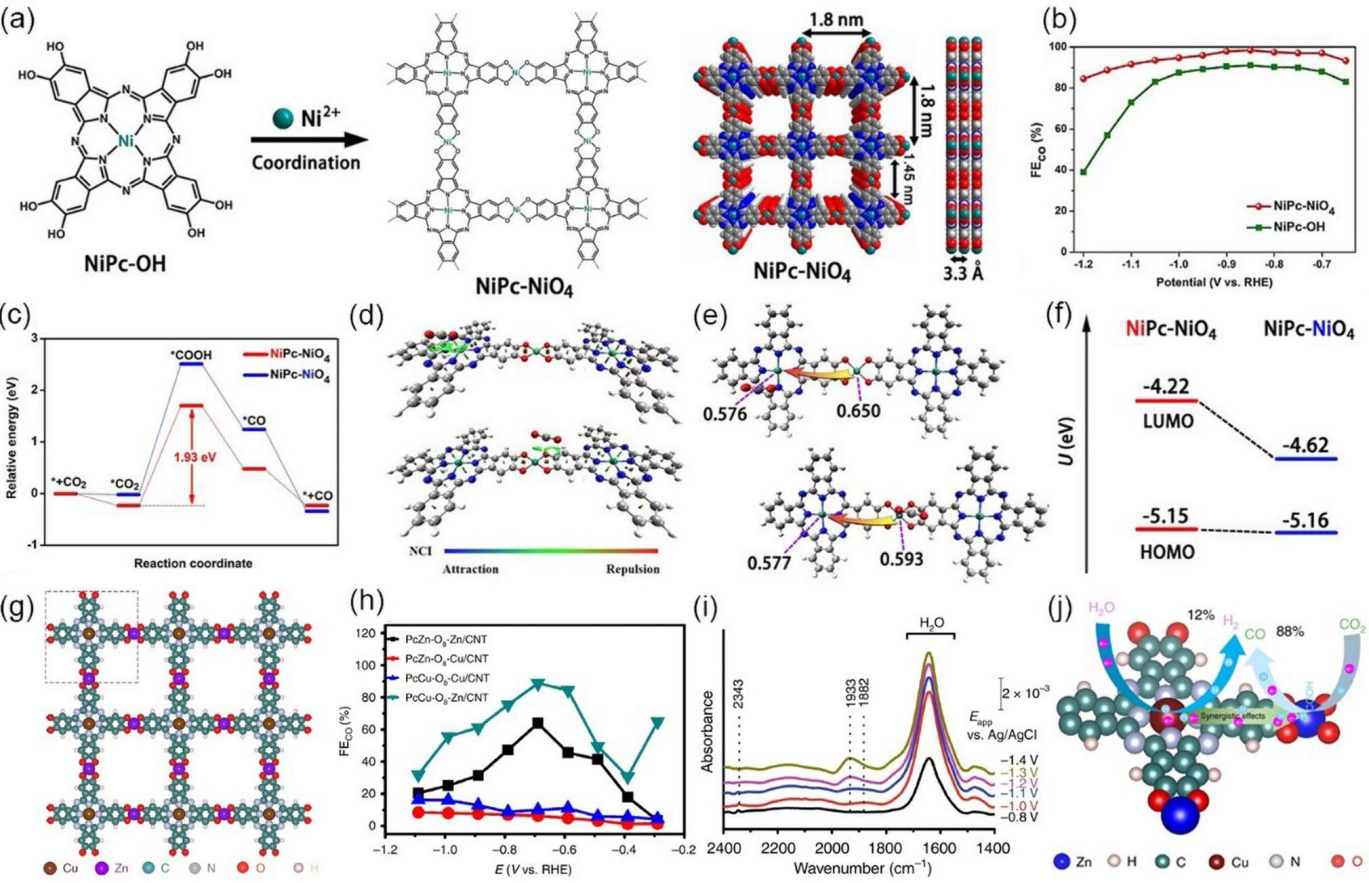
**a** Illustration of the preparation steps of NiPc-NiO_4_. Top and side view of their structures with 2 × 2 square grids in AA-stacking mode. **b** Faradaic efficiencies of CO. **c** Calculated energy diagrams for CO_2_-to-CO conversion on two proposed active sites in NiPc-NiO_4_. **d** The non-covalent interaction (NCI) between CO_2_ and NiPc-NiO_4_ structure. **e** Mulliken charge of different Ni atoms in NiPc-NiO_4_. **f** Energy level of HOMO and LUMO of different Ni atoms in NiPc-NiO_4_ when introducing CO_2_. Reproduced with permission from Ref. [99]. **g** Schematic structure of PcCu-O_8_-Zn (the dashed rectangular indicates the unit cell). **h** Faradaic efficiency of CO for PcCu-O_8_-Zn/CNT, PcCu-O_8_-Cu/CNT, PcZn-O_8_-Zn/CNT and PcZn-O_8_-Cu/CNT at different potentials. **i** Operando surface-enhanced infrared absorption (SEIRA) spectro-electrochemical analysis of PcCu-O_8_-Zn/CNT in CO_2_-saturated 0.1 M KHCO_3_. **j** Schematic HER and CO_2_RR reaction process of PcCu-O_8_-Zn. Reproduced with permission from Ref. [101]

Choosing electron-donating ligands as well as their orientation and bonding arrangements is an efficient strategy to improve the conductivity of MOFs, these functional groups can increase the conductivity of MOFs by creating pathways for the flow of electrons and increasing the density of free electrons in the material [114–116]. Owing to the high overlap of d-*π* conjugation orbitals between the nickel node and the planar Ni-phthalocyanine-substituted X (X: o-phenylenediamine or catechol), Zhang et al. [98] employed Ni-phthalocyanines (NiPc) as the building block for the construction of a porous intrinsically conductive two-dimensional (2D) MOF (NiPc-Ni(NH)_4_). The 2D NiPc-Ni(NH)_4_ MOF showed a high electrical conductivity of 2.39 × 10^–4^ S m^−1^, and NiPc-Ni(NH)_4_ nanosheets showed outstanding CO_2_-to-CO electrocatalytic performance with a high CO selectivity of 96.4% at −0.7 V *vs.* RHE in CO_2_-saturated 0.5 M KHCO_3_ electrolyte. DFT calculations revealed that the active site is Ni-N_4_ moiety in NiPc. The presence of square planar Ni(NH)_4_ nodes can efficiently accelerate the proton/electron transport to the active sites, thus accelerating the reaction kinetics during the electrochemical CO_2_RR. Following the same strategy, Yi et al. [99] employed NiPc as the building block to prepare the phthalocyanine-based MOF (Ni–Pc–NiO_4_) via the solvothermal synthesis method (Fig. 5a), and then exfoliated the bulk powder into 2D nanosheets through high-frequency sonication at room temperature. A two-contact probe method was applied to test the electrical conductivity of NiPc-NiO nanosheets at room temperature. The 2D NiPc-NiO nanosheets showed good electrical conductivity (4.8 × 10^–5^ S m^−1^). Such good electrical conductivity would be beneficial for the electron transfer to the active sites during CO_2_RR, thereby improving electrochemical activity and energy conversion efficiency. As expected, when tested in CO_2_-saturated 0.5 M KHCO_3_ electrolyte, NiPc-NiO nanosheets showed a high CO FE of > 90% in a wide potential range from -0.65 to -1.1 V *vs.* RHE, reaching the maximum of 98.4% at -0.85 V *vs.* RHE, surpassing NiPc-OH monomer (Fig. 5b). X-ray absorption spectroscopy (XAS) and X-ray photoelectron spectroscopy (XPS) analysis confirmed that the NiPc sites and NiNO_4_ nodes in NiPc-NiO_4_ were well maintained after the CO_2_RR, demonstrating the superior structural stability of NiPc-NiO_4_. Theoretical calculations show that the Gibbs free energy of the rate-determining step (RDS) (formation of *COOH intermediate) on NiPC (1.93 eV) is significantly lower than that on the NiO_4_ node (2.53 eV), revealing that NiPc is the active site for the electrochemical conversion of CO_2_ to CO (Fig. 5c). Compared to NiO_4_ node, NiPc showed stronger Van der Waals interaction with CO_2_ molecules (Fig. 5d) in the more electron-rich environment (Fig. 5e). It is worth noting that the lowest unoccupied molecular orbital (LUMO) energy level of NiPc-NiO_4_ shifts from −4.22 to −4.62 eV when the CO_2_ molecule moves from NiPc to NiO_4_ node (Fig. 5f), revealing the excellent reducibility of NiPc. In addition, another 2D conductive MOF was reported by Majidi et al. [100]. They use a catechol-based linker, tetrahydroxyquinone (THQ), to synthesize the Cu-based 2D conductive MOF (Cu-THQ) nanoflakes, and the product shows good electrical conductivity of 1.5 × 10^–7^ S cm^−1^. The presence of the THQ linker in Cu-THQ nanoflakes resulted in a large distance between Cu centers, which not only keeps the Cu center from agglomeration but also ensures the reoxidation of the reduced Cu center during the CO_2_RR process. When tested in a hybrid electrolyte (1 M C_5_H_14_ClNO + 1 M KOH), Cu-THQ showed high CO current densities and low overpotential. DFT calculations revealed that the higher the CO coverage, the lower the free energy for CO adsorption on the Cu surface, resulting in a high CO production rate. Zhong et al. [101] also designed layered 2D conductive MOFs (PcCu-O_8_-Zn) with bimetallic centers (ZnO_4_/CuN_4_) to improve electrocatalytic CO_2_RR activity (Fig. 5g). They immobilized a mixture of PcCu-O_8_-Zn and carbon nanotubes (CNTs) onto a carbon paper substrate as the working electrode and tested its CO_2_RR performance in 0.1 M KHCO_3_. As shown in Fig. 5h, at −0.7 V vs. RHE, the PcCu-O_8_-Zn/CNT can effectively reduce CO_2_ to CO with a FE of 88%. Moreover, such performance could be sustained for over 10 h, demonstrating excellent stability. They conducted a series of operando experiments and theoretical calculation to demonstrate that the synergistic effect of Cu and Zn sites in PcCu-O_8_-Zn is essential for the high catalytic activity and selectivity toward CO production (Fig. 5i). The ZnO_4_ units facilitate CO_2_ reduction, while the CuN_4_ units promote proton and electron transfer during the reaction process (Fig. 5j).

**Fig. 6:**
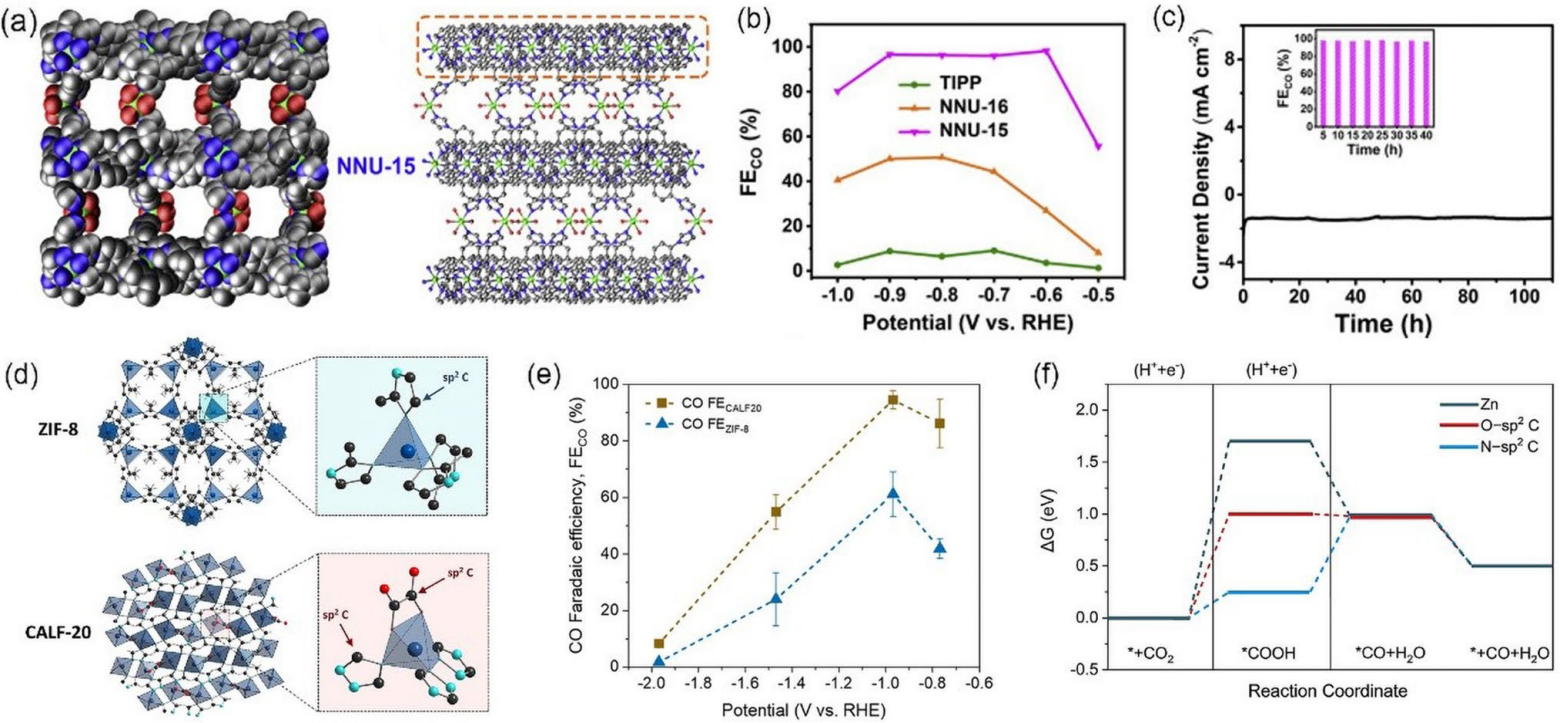
**a** 3D frameworks for NNU-15 along the b-axis and 3D frameworks with the open holes in NNU-15. **b** Faradic efficiencies for CO of TIPP, NNU-16 and NNU-15. **c** Durability test of NNU-15 at the potential of -0.6 V versus RHE (inset: CO FF at different time). Reproduced with permission from Ref. [103]. **d** Schematic illustration of the crystal structure of ZIF-8 and CALF20, including the chemical structure of polyhedron zinc nodes. **e** CO Faradaic efficiencies at different applied potentials for CALF20 and ZIF-8 in 1.0 M KOH. **f** Calculated free energy diagram for electrochemical reduction of CO_2_ to CO over CALF20 at U = 0.00 V vs RHE (the dashed lines are simply to guide the eye). Reproduced with permission from Ref. [104]

Ligand engineering can also modulate the coordination environment on metal center, thus boosting the activity of electrochemical CO_2_RR. By virtue of 1,10-phenanthroline doping, Dou et al. [102] synthesized a ligand-doped product (ZIF-A-LD, ZIF-A: ZIF-8 was activated to generate open Zn sites) with excellent charge transfer ability. Then, the ZIF-A-LD was mixed with carbon black to prepare a working electrode (ZIF-A-LD/CB). When tested in 0.1 M KHCO_3_, ZIF-A-LD/CB exhibited a higher CO FE compared to pristine ZIF-8. DFT calculations revealed that the charge transfer from the dopant phenanthroline molecule (excellent electron-donating ability) to the adjacent *sp*^2^ C sites in the imidazolate enables stronger electrons movement from the active sites to the antibonding orbitals of CO_2_, which facilitates *COOH formation and boost CO_2_RR activity. Huang et al. [103] reported a stable MOF (NNU-15), Co(OH)_2_(H_2_O)_2_(Co-TIPP) (TIPP = [5,10,15,20-tetra (4-imidazol-1-yl)phenyl]porphyrin), which contains two OH^−^ coordinated Co ions to mimic the active surface status of the catalysts under alkaline CO_2_RR conditions (Fig. 6a). It exhibits an outstanding ability to capture and convert the CO_2_ molecule. When tested in CO_2_-saturated 0.5 M KHCO_3_ electrolyte, NNU-15 shows a high FE of 99.2% for CO at -0.6 V *vs.* RHE (Fig. 6b) and excellent long-term stability of 110 h (Fig. 6c). The NNU-15-CO_2_ intermediate can be detected during the CO_2_RR process, demonstrating that the metal catalytic center of MOF can cooperate with OH^−^ to capture and activate the CO_2_ molecule to form the HCO_3_^−^-chelating active center, and thus getting a remarkable activity for CO_2_ conversion to CO. In another study, Al-attas et al. [104] take a ligand engineering strategy to enhance the efficiency of CO_2_ conversion to CO for Zn-based MOFs. They prepared two types of Zn-based MOFs with different ligands. As shown in Fig. 6d, one is Zn_2_(Tz)_2_Ox, which is assembled from Zn ions and 1,2,4-triazolate ligand (Calgary Framework 20, CALF20), and the other is Zn(MeIm)_2_ that is assembled from Zn ions and 2-methylimidazolate ligand (zeolitic imidazolate framework-8, ZIF-8). They used a gas-diffusion electrode in a flow electrolyzer (circulated with 1.0 M KOH solution) to assess the activity and selectivity of Zn-based MOFs toward CO_2_RR. As shown in Fig. 6e, CALF20 shows higher CO FE than ZIF-8 over the whole potential range and the highest FE for CO production is located at −0.97 V *vs.* RHE with a partial current density of − 32.8 mA cm^−2^. The difference in the Zn oxidation state between CALF20 and ZIF-8 is confirmed by soft X-ray spectra, indicating that the Zn site in CALF20 exhibited a lower electron density due to the electron-withdrawing character of the triazole ligand. DFT calculations revealed that the C_2_O_2_ bridge promotes stronger charge transfer, and the *sp*^2^ C sites in azolate are the active sites with low overpotential for CO_2_RR. More electrons are produced on the adjacent active sites of the azole ligand and facilitated *COOH formation (Fig. 6f), resulting in the high current density and FE toward CO production.

#### MOF-Derived Materials

**Fig. 7:**
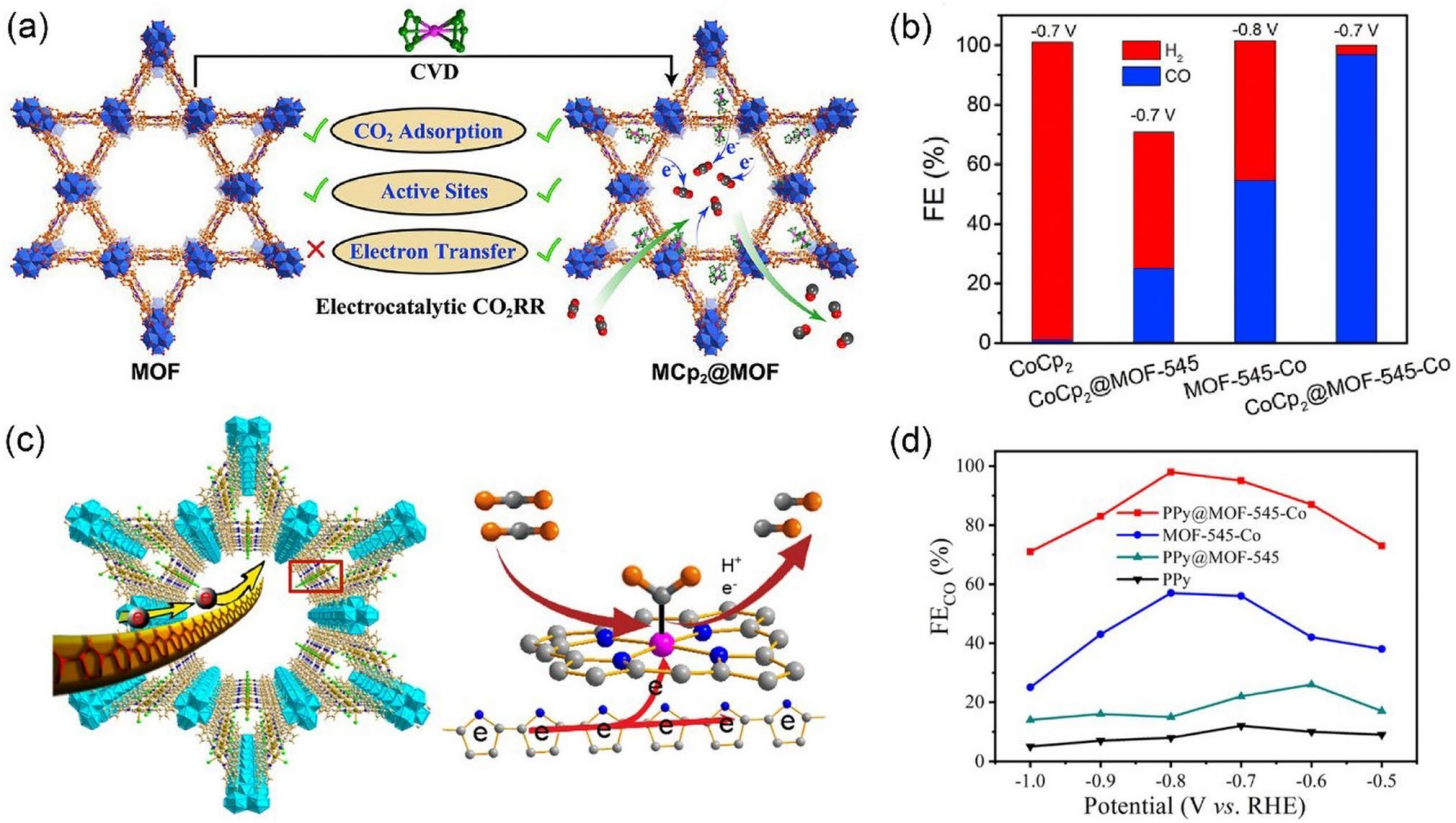
**a** Comparison of MCp_2_@MOF and MOF in electrocatalytic CO_2_RR. **b** Electrocatalytic CO_2_RR performances of CoCp_2_@MOF-545-Co and comparative samples. Reproduced with permission from Ref. [105]. **c** Schematic presentation for the advantages of PPy in the channel of MOF-545-Co for electrocatalytic CO_2_RR. **d** FE_CO_ of PPy@MOF-545-Co and contrastive samples measured under different voltages. Reproduced with permission from Ref. [106]

Generally speaking, MOFs are considered to be insulators or poor conductors due to the insulating nature of the organic ligands. The poor conductivity of MOFs is an important limitation for their CO_2_ catalysis applications. One simple strategy to improve charge transport is mixing MOFs with conductive carbon, but this can result in the loss of mass activities. A more effective way to overcome charge transport is introducing guest redox molecules into the frameworks. For example, Xin et al. [105] introduced cobaltocene into MOF-545-Co to prepare CoCp_2_@MOF-545-Co through a facile chemical vapor deposition method (Fig. 7a). Compared with MOF-545-Co, CoCp_2_@MOF-545-Co shows high CO_2_-to-CO activity and the maximum FE of CO was dramatically increased from 55% (at −0.8 V *vs.* RHE) to 97% (at −0.7 V *vs.* RHE) (Fig. 7b). The excellent CO_2_ reduction performance of CoCp_2_@MOF-545-Co was demonstrated to originate from the synergy effect of MOF-545-Co and CoCp_2_. There are several reasons accounting for this consequence. Firstly, in metallocene electron orbits, the participation of d-orbits enlarges the delocalized π-electron from the cyclopentadienyl ring, thus making metallocene a perfect electron-donating or carrying unit. Secondly, the presence of porphyrin ring ligands in MOF-545-Co are ideal charge transfer carriers, and metal in the center of the porphyrin ring might serve as an efficient electron acceptor and active catalysis center. DFT calculations revealed that the introduction of metallocene can create continuous electron transfer channels in MOFs and the strong binding interaction between metalloporphyrin and reactants during the CO_2_RR process can largely reduce the adsorption energy of CO_2_. This work shows that the conductivity of MOF materials is a crucial factor that affects the efficiency of electrochemical CO_2_RR. Furthermore, it could open a new avenue to develop highly selective CO_2_RR electrocatalysts. In another study, Xin et al. [106] followed the same strategy to enhance the conductivity of MOFs materials. They insert the conducting polypyrrole (PPy) molecule into the channel of MOF-545-Co through a facile in-situ low-temperature polymerization method. As shown in Fig. 7c, in the obtained PPy@MOF-545-Co, the PPy molecule acts like a cable in the MOF channel, it can boost the conductivity of MOFs and facilitate electrons transfer to the active center of Co-TCPP. The charge-transfer resistance of PPy@MOF-545-Co (7.5 Ω) was proved to be much lower than that of MOF-545-Co (12.5 Ω), indicating that it possessed a better electron transfer ability. Subsequently, PPy@MOF-545-Co was applied as the working electrode and its electrocatalytic CO_2_RR activity was tested using an H-type cell in the CO_2_-saturated 0.5 M KHCO_3_ electrolyte. As shown in Fig. 7d, PPy@MOF-545-Co exhibits excellent catalytic activity toward CO with a maximum FE up to 98%, which is much higher than its MOF-545-Co counterpart. The CO partial current density was also remarkably higher than the samples in the controlled experiments. Furthermore, after continuous electrolysis at -0.8 V *vs.* RHE for 10 h, the chemical structure of PPy@MOF-545-Co remained unchanged, revealing its excellent stability. The charge-transfer resistance of PPy@MOF-545-Co (7.5 Ω) was also lower than that of MOF-545-Co (12.5 Ω), indicating that it possessed a better electron transfer ability. By impregnating guest redox molecules into the framework structure, the charge can transfer directly from the guest molecule to the metalloporphyrin center and the strategy was demonstrated to significantly enhance the charge transfer efficiency.

#### MOF-Derived Single-Atom Catalysts Materials

**Fig. 8:**
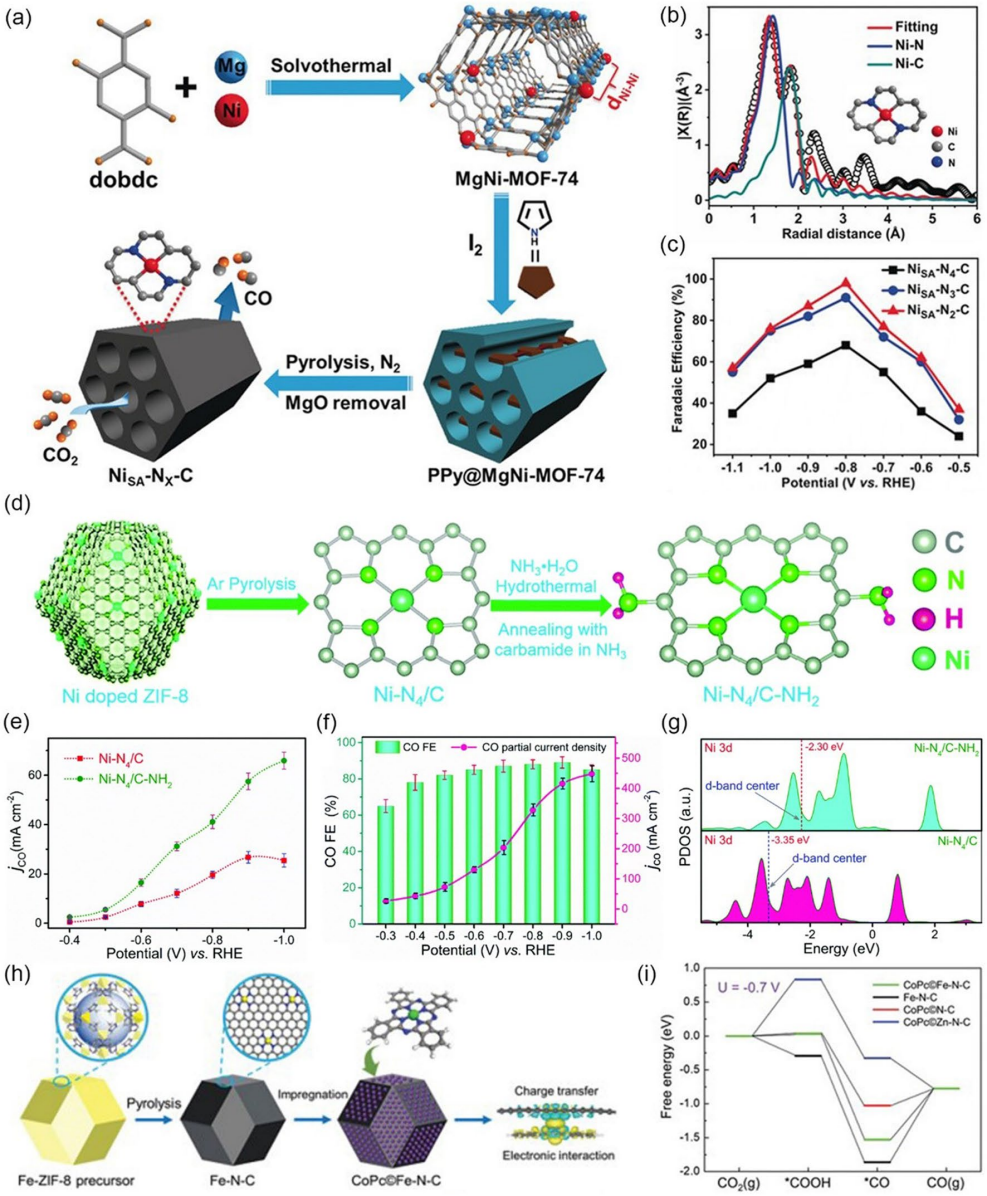
**a** Illustration showing the host–guest cooperative protection strategy for the fabrication of Ni_SA_-N_x_-C catalysts for electrocatalytic CO_2_ reduction. **b** EXAFS fitting and optimized model for NiSA-N_2_-C. **c** FEs of CO at different applied potentials in the CO_2_-saturated 0.5 M KHCO_3_ electrolyte. Reproduced with permission from Ref. [107]. **d** Schematic of the synthesis process for Ni–N4/C–NH_2_. **e** CO partial current density of Ni–N_4_/C and Ni–N_4_/C–NH_2_. **f** Electrocatalytic activity of Ni–N_4_/C–NH_2_ in the flow cell. **g** Projected DOS of Ni 3*d* in Ni–N_4_/C–NH_2_ and Ni–N_4_/C. Reproduced with permission from Ref. [108]. **h** Schematic illustration for the preparation of CoPc©Fe–N-C. The image on the far right is the calculated electron density difference of the CoPc©Fe–N-C structure. (Blue and yellow contours present electron depletion and electron accumulation, respectively. The isosurface level is set to be 0.0006 e Bohr-3). **i** Calculated free energy diagram for the CO_2_RR to CO at U = -0.7 V versus RHE on the Fe site in CoPc©Fe–N-C, Fe site in Fe–N-C, Co site in CoPc©N–C, and Zn site in CoPc©Zn-N–C, respectively. Reproduced with permission from Ref. [109]

Recently, single-atom catalysts have been demonstrated to show excellent catalytic performance for various reactions due to their controllable properties and high atom utilization efficiency. Particularly, MOFs are ideal sacrificial templates to fabricate SACs because of the uniformly dispersed metal sites and abundant heteroatoms to immobilize the single metal site, resulting in a number of catalysts with high selectivity and remarkable activity for CO_2_RR. For instance, Gong et al. [107] employ MgNi-MOF-74 as precursors to produce Ni_SA_-Nx-C SACs (x is N coordination numbers) (Fig. 8a). The presence of Mg^2+^ in MgNi-MOF-74 can regulate and control the interatomic distance between adjacent Ni atoms, while N atoms from pyrolyzed polypyrrole (PPy) serve as anchoring sites to stabilize the Ni atoms. By controlling the pyrolysis temperature, they prepared three single-atom Ni catalysts with different N coordination numbers. Among them, Ni_SA_-N_2_-C (Fig. 8b) exhibits the highest selectivity for CO and the maximum FE is 98% at −0.8 V *vs.* RHE in CO_2_-saturated 0.5 M KHCO_3_ (Fig. 8c). This work not only provides a strategy for the fabrication of SACs, but also opens an avenue to enhance the activity of SACs for CO_2_RR by controlling the metal coordination environment. Similarly, Chen et al. [108] report an amination strategy to regulate the electronic structure of SACs. As shown in Fig. 8d, they successful synthesized Ni–N_4_/C–NH_2_ SACs by a two-step method. A gas-tight H-type cell containing CO_2_-saturated 0.5 M KHCO_3_ electrolyte was used to evaluate the electrocatalytic activity of Ni–N_4_/C–NH_2_ and a maximum CO FE of 96.2% was achieved. Although the CO FE is slightly lower than that of Ni–N_4_/C (98.1%), the CO partial current density of Ni–N_4_/C–NH_2_ is found to be significantly enhanced, which is 2.5 times that of Ni–N_4_/C at −1.0 V vs. RHE (Fig. 8e). In order to meet requirements for industrial CO_2_-to-CO production, they applied a gas-fed flow cell equipped with a GDE to solve the mass transfer limitation caused by low solubility of CO_2_ in aqueous electrolysis. As shown in Fig. 8f, Ni–N_4_/C–NH_2_ achieves a remarkable CO partial current density of 447.6 mA cm^−2^ at −1.0 V vs. RHE, which is 7.0 times that in an H-type cell and is much larger than that of Ni–N_4_/C (250 mA cm^−2^), suggesting that amination treatment can indeed boost catalytic activity. DFT calculations revealed that the electronic structure of M–N/C catalysts are regulated by amino-modification (Fig. 8g), which enhances the adsorption energies of the reaction intermediates and accelerates the charge transfer rate, thus promoting the CO_2_ activation and transformation process. Moreover, phthalocyanine-based MOFs can also be used as catalyst regulators to modify the electronic structure of metal center and enhance the CO_2_RR to CO. For example, Lin et al. [109] took a synergistic catalysis strategy to boost the CO_2_RR activity by anchoring Fe–N sites into CoPc (CoPc©Fe–N–C) through a sequential pyrolysis and post-impregnation method (Fig. 8h). CoPc©Fe–N–C shows CO FE of above 90% over the measured potential range of -0.13 to -0.84 V *vs.* RHE, exceeding its counterpart. Significantly, CO current density reaches 275.6 ± 27.0 mA cm^−2^ at −0.84 V *vs.* RHE, which is sufficient to satisfy industrial requirements. The strong interaction between CoPc and Fe–N-C also reduces the *CO poisoning and accelerates desorption of CO. DFT calculations revealed that the adsorption energy of *CO and *H on CoPc©Fe–N–C is lower than that of Fe–N–C, while the *COOH formation energy does not change much (Fig. 8i), demonstrating unprecedented synergistic catalysis effect toward CO_2_RR.

### Formate/Formic acid

HCOOH/HCOO^−^ is not only a hydrogen storage chemical with high energy density, but is also widely applied in the green synthesis of a range of petrochemicals in modern energy systems [117–119]. Moreover, among all kinds of products from CO_2_RR, HCOOH possesses the highest profit per mole of electrons, exhibiting good economic prospects in large-scale industrial production [75]. To date, there are many MOF-related catalysts showing remarkable activity and selectivity for the production of HCOOH by CO_2_RR. Several representative examples are shown in Table 3, we will discuss their structure–activity relationships from the perspective of metal composition, such as In, Sn, and Bi.

**Table 3:** Representative MOF-related catalysts for selectively catalyzing the electrochemical reduction of CO_2_ to HCOOH

Catalyst	MOF precursor	Electrolyte	FE (%)	Potential (V *vs.* RHE)	Refs.
In-MOF **1**	–	0.5 M KHCO_3_	89.6	−1.3	[120]
CPs@V11	In-MOF	0.5 M KHCO_3_	90.1	−0.84	[121]
In_2_O_3_-x@C	MIL‑68	1 M KOH	97	−1.0	[122]
Sn-doped ZIF8	ZIF-8	0.5 M KHCO_3_	74	−1.1	[123]
Sn-N6-MOFs	–	0.5 M KHCO_3_	85.1	−1.23	[124]
Sn(101)/SnO_2_/C-500	Sn-MOF	0.5 M KHCO_3_	93.3	−0.8	[125]
Bi-BTC-D	–	0.5 M KHCO_3_	95.5	−0.86	[126]
2D CAU-17	–	0.1 M KHCO_3_	92.2	−0.9	[127]
Bi NSs	CAU-17	1 M KOH	98	−0.48	[128]
Bi_2_O_3_@C-800	CAU-17	0.5 M KHCO_3_	92	−0.9	[129]
CAU-17-fiber-400	CAU-17	0.1 M KHCO_3_	96.4	−0.9	[130]
BiInO-0.67@C	BiIn-MOFs	0.5 M KHCO_3_	91	−0.9	[131]
Bi-ene	Bi-MOFs	0.5 M KHCO_3_	100	−0.83	[132]
Bi_2_O_2_CO_3_	Bi-MOFs	0.5 M KHCO_3_	96.1	−0.67	[133]
Bi NPs	CAU-17	0.1 M KHCO_3_	92	−1.1	[134]
Cu dendrites	Cu-MOFs	0.5 M BMIM-BF_4_	98.2	−1.22	[135]
BiIn alloy NPs	Bi-MOFs	0.1 M KHCO_3_	97.6	−1.1	[136]
Bi NPs	CAU-7	0.5 M KHCO_3_	95	−0.97	[137]

#### In-based MOF

**Fig. 9:**
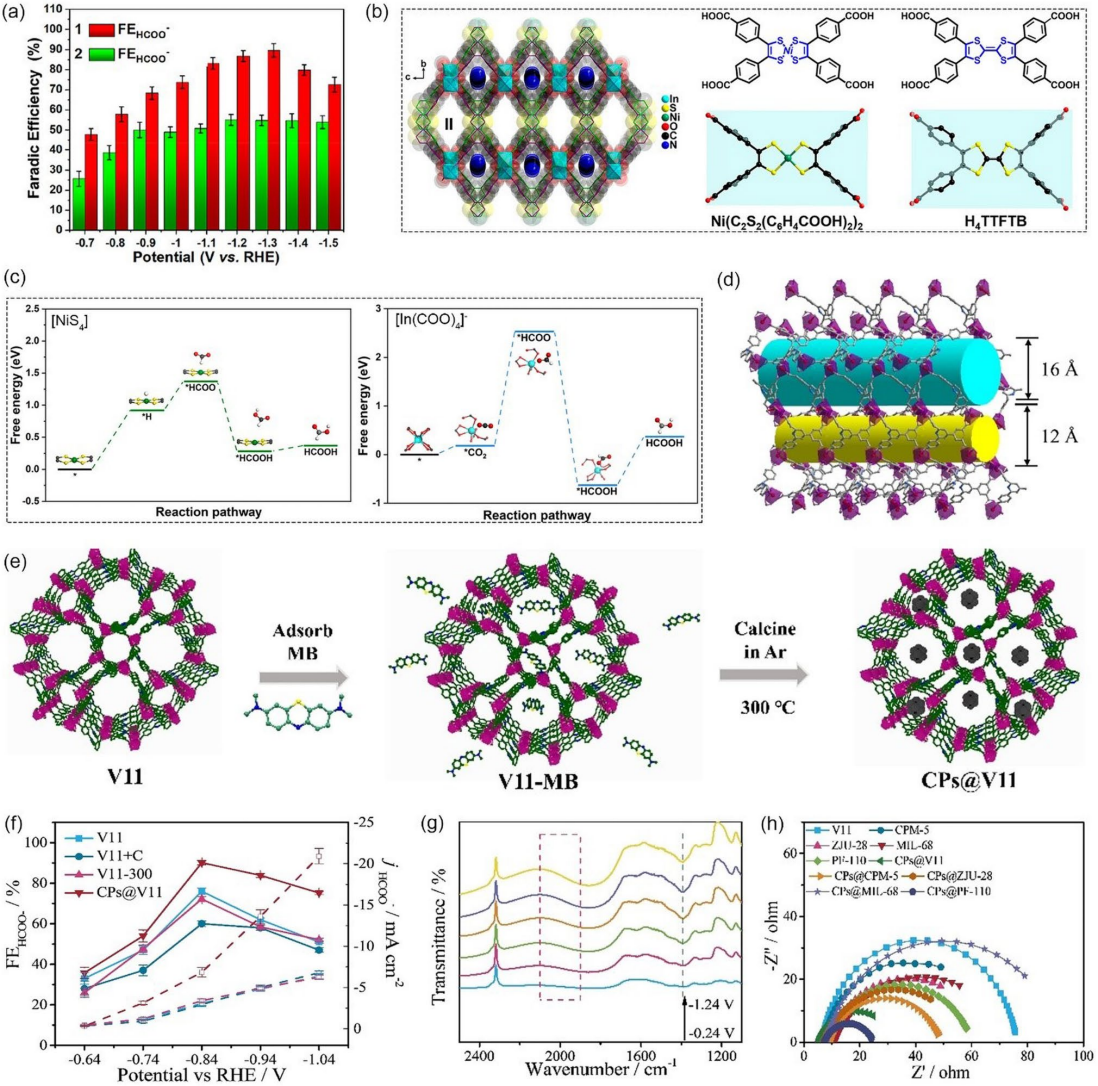
**a** Plots of FE_HCOO–_ for In-MOF **1** and In-MOF **2** versus applied potential. **b** Crystal structure of In-MOF **1** viewed along the a-axis, showing three rhombic pores and a two-fold interpenetrated framework (hydrogen atoms have been removed for clarity), and structures of ligands [Ni(C_2_S_2_(C_6_H_4_COOH)_2_)_2_] and H_4_TTFTB with different conformations. **c** Proposed reaction paths for the formation of HCOOH on the [NiS_4_] and [In(COO)_4_].^–^ sites. Reproduced with permission from Ref. [120]. **d** The view of the two types of 1D channels in V11. **e** Illustration of the preparation process of CPs@V11. **f** The comparison of the FE HCOO- and the j HCOO- for various samples. **g** Potential-dependent in situ FTIR spectra of CPs@V11. **h** Nyquist plots for the samples in CO_2_-saturated 0.5 M KHCO_3_ electrolyte. Reproduced with permission from Ref. [121]

Among the numerous catalyst materials that were studied, In-based catalysts were found to be selective toward the production of HCOOH. Recently, with the marriage of biomimetic and catalytic technology, a new revolution has emerged in structure design of In-based catalysts, which has significantly promoted the development of the CO_2_RR field. For example, by mimicking the active [NiS_4_] sites of formate dehydrogenase and CO-dehydrogenase, Zhou et al. [120] prepared a crystalline enzyme-mimicking three-dimensional In-based MOF, (Me_2_NH_2_^+^){In^III^-[Ni(C_2_S_2_(C_6_H_4_COO)_2_)_2_]} 3DMF 1.5H_2_O (In-MOF **1**, DMF = N,N-dimethylformamide), with excellent chemical and thermal stabilities. Compared to the isomorphic MOF, (Me_2_NH_2_^+^) [In^III^-(TTFTB)]·0.7C_2_H_5_OH·DMF (In-MOF **2**, TTFTB = tetrathiafulvalene-tetrabenzoate), In-MOF **1** exhibited higher HCOOH selectivity during CO_2_RR and the FE increased from 54.7% to 89.6% (-1.3 V *vs.* RHE) (Fig. 9a), demonstrating that the presence of unsaturated [NiS_4_] sites in MOFs can significantly enhance the CO_2_RR activity (Fig. 9b). DFT calculations further revealed that the formation energy of *HCOO intermediate on In-MOF **1** is much lower than that of In-MOF **2** (Fig. 9c), the introduced unsaturated [NiS_4_] sites are proved to be the absorption and catalytic site for CO_2_-to-HCOOH. Another In-based MOF was reported by Zhu et al. [121]. They constructed a stable 3D In-MOF (V11), {(Me_2_NH_2_) [In(BCP)]·2DMF}_n_ (H_4_BCP = isophthalic acid) consisting two types of channels (1.6 and 1.2 nm diameter) (Fig. 9d). Subsequently, methylene blue molecules were introduced into the framework of V11 and then converted to carbon nanoparticles (CPs), forming V11-supported CPs (CPs@V11) (Fig. 9e). When tested in CO_2_-saturated 0.5 M KHCO_3_ electrolyte, the catalytic performance of CPs@V11 (methylene blue mass load of 10%) was significantly improved and the highest FE_HCOOH_ is 90.1% at −0.84 V *vs.* RHE. In addition, it also exhibited higher HCOOH partial current density than that of pure V11 at the same potential (Fig. 9f), obviously, the introduction of CPs via pyrolysis of MB greatly enhances the catalytic activity of the MOF. In-situ Fourier Transform infrared spectroscopy (FT-IR) spectra at different potentials showed that there are obvious absorption peaks of *HCOO intermediate at 1394 cm^−1^, which gradually increase with decreasing potential (Fig. 9g). The introduced CPs not only improves electrochemical active surface area (ECSA) but also increases the conductivity, thus facilitating charge transfer (Fig. 9h) and enhancing the catalytic performance of the MOFs in terms of activity and selectivity. In addition, Qiu et al. [122] also synthesized an efficient In-based electrocatalyst (In_**2**_O_**3**_-x@C nanocorn) for converting CO_2_ to HCOOH through a two-step process involving In MOF preparation and carbonization. When tested in 1 M KOH electrolyte, such electrocatalyst exhibited excellent catalytic activity and stability, due to its unique nanocorn structure, high concentration of active sites, and favorable electronic transfer properties. The operando experiments have confirmed that In^3+^ species as the catalytic active sites for the production of HCOOH. Furthermore, DFT calculations have revealed that the presence of oxygen vacancies creates an electron-rich environment for the In^3+^ active sites, leading not only to an enhancement of the reducing power at the active sites but also to a reduction in the energy barrier for electron transfer.

#### Sn-based MOF

**Fig. 10:**
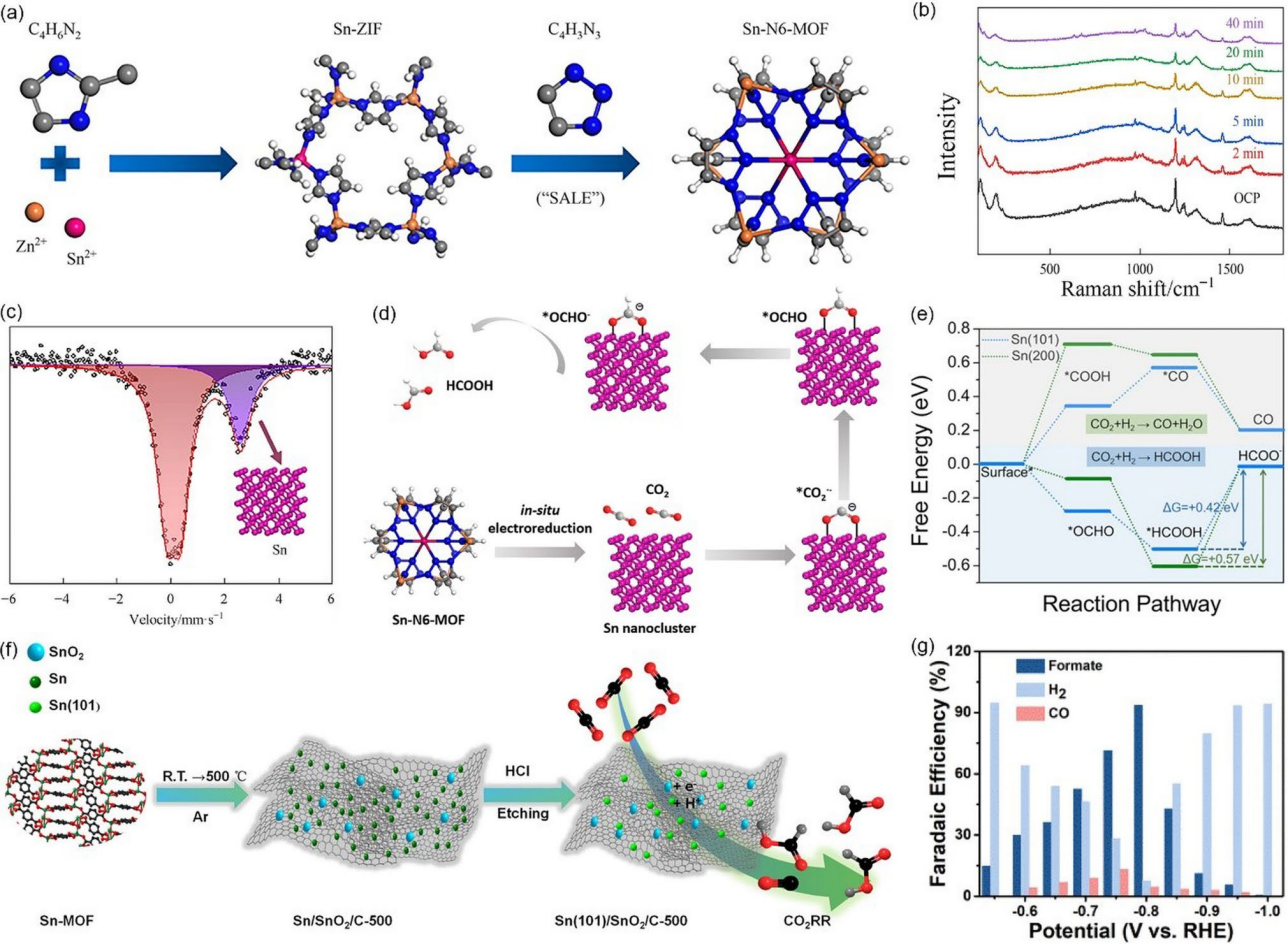
**a** Diagram of the synthetic procedures for Sn-N6-MOF. **b** In-situ Raman spectra measured with varying the acquiring time at -1.23 V vs. RHE in CO_2_-saturated 0.5 M KHCO_3_ aqueous. **c** Room temperature 119 Sn Mössbauer spectra acquired after maintained at -1.23 V vs. RHE in CO_2_-saturated 0.5 M KHCO_3_ aqueous for 1 h. **d** Proposed reaction pathway for the formation of HCOOH over Sn-N_6_-MOF. Reproduced with permission from Ref. [124]. **e** Calculated free-energy diagrams for HCOO^−^,CO formation. **f** Schematic illustration showing the preparation of Sn(101)/SnO_2_/C catalysts. **g** Electrocatalytic property of Sn(101)/SnO_2_/C-500. Reproduced with permission from Ref. [125]

Sn-based MOFs are another class of catalysts with excellent HCOOH selectivity. Geng et al. [123] prepared Sn-doped ZIF8 catalysts via an ion-exchange strategy, the method can efficiently integrate Sn into the node of ZIF-8 while preserving the whole framework structure. When tested in CO_2_-saturated 0.5 M KHCO_3_, Sn-doped ZIF8 showed high HCOOH activity and selectivity of 74% FE at −1.1 V vs. RHE. And the spatially separated Sn atoms are proposed to be responsible for the superior CO_2_RR activity of Sn-doped ZIF8. Similarly, Deng et al. [124] also replaces the Zn metal nodes in ZIF8 by Sn doping, and then follows a solvent-assisted linker exchange (SALE) process to obtain the Sn-N6-MOF catalyst (Fig. 10a). When tested in CO_2_-saturated 0.5 M KHCO_3_, such catalyst achieved excellent selectivity for HCOOH with FE_HCOOH_ up to 85.1% at −1.23 V *vs.* RHE. In-situ Raman spectra indicate the organic ligands in MOF are gradually lost during the CO_2_RR (Fig. 10b). Furthermore, ex-situ ^119^Sn Mössbauer results demonstrated the presence of zero-valence Sn metal after continuous electrolysis for 1 h at −1.23 V *vs.* RHE (Fig. 10c). A series of experiments results have revealed that Sn-N6-MOF catalyst undergoes in-situ structural reconstruction during the CO_2_RR process and then generates Sn nanoclusters, which are the real active sites for producing HCOOH (Fig. 10d). In a previous study, Wu et al. [125] also discovered that Sn(101) crystal plane is more favorable for the formation of HCOOH (Fig. 10e). They synthesized a Sn-MOF (Sn_3_O(1,4-BDC)_2_) precursor to prepare a series of Sn(101)/SnO_2_/C composites via the calcination and acidic etching processes (Fig. 10f). The Sn(101)/SnO_2_/C-500 (500 is the carbonization temperature) exhibits high selectivity toward HCOOH and achieved a FE_HCOOH_ up to 93.3% at −0.8 V *vs.* RHE (Fig. 10g). However, the FE_HCOOH_ of such catalyst is gradually reduced from 93.3% to 72.8% after continuous electrolysis for 13 h at −0.8 V *vs.* RHE.

#### Bi-based MOF

**Fig. 11:**
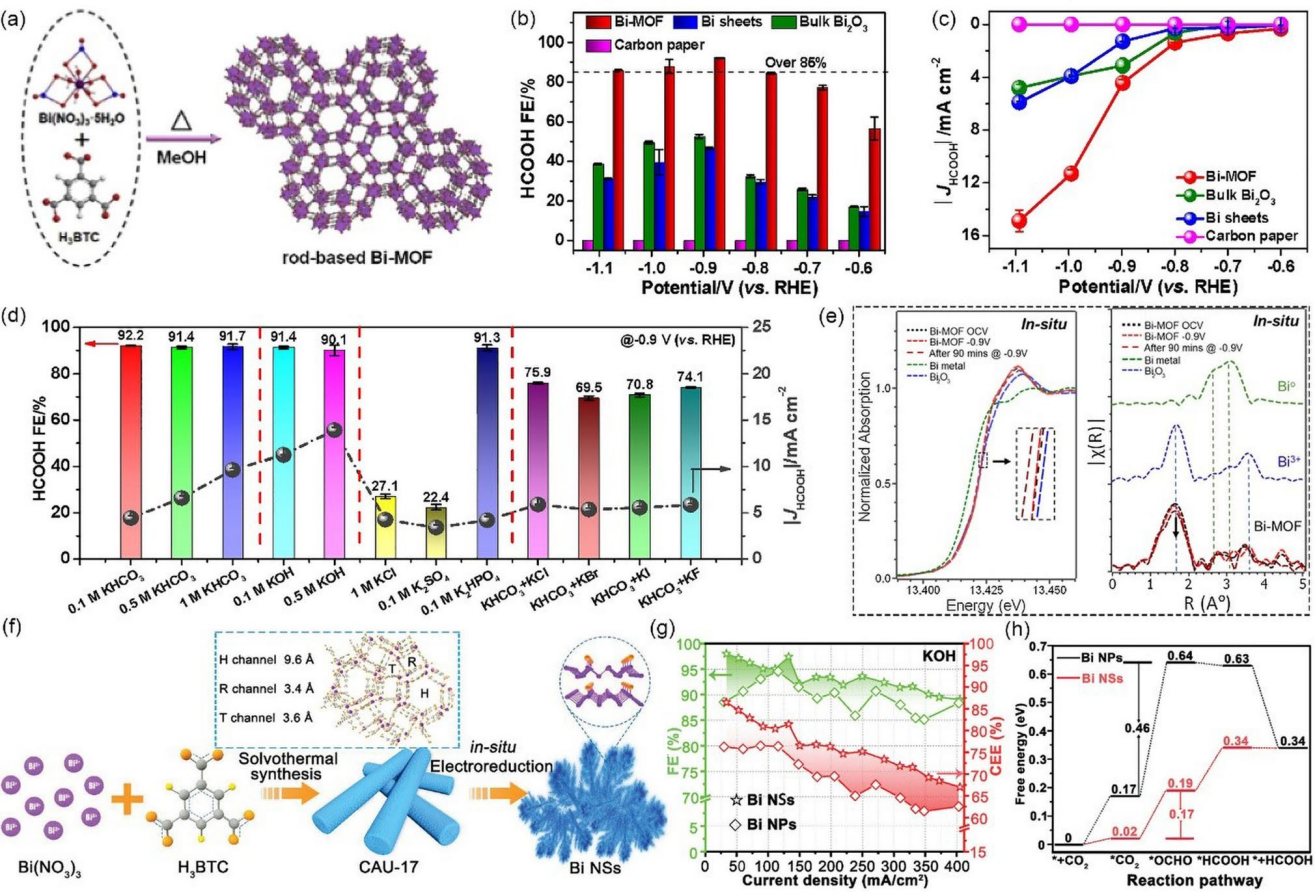
**a** Schematic depiction of the formation of Bi-MOF. Bi-O polyhedra shown in the MOF crystal structure are indicated in purple. **b** HCOOH FEs, **c** HCOOH partial current densities of Bi-MOF, Bi sheets, bulk Bi_2_O_3_, and carbon paper electrodes within a potential window of −0.6 to −1.1 V in CO_2_-saturated 0.1 M KHCO_3_ solution. **d** HCOOH FEs and HCOOH partial current densities of Bi-MOF in various electrolytes. **e** Comparison of Bi L_3_-edge X-ray absorption fine structure (XAFS) of Bi-MOF along with those for Bi metal and Bi_2_O_3_ as reference standards and represent their respective XANES and Fourier transform of EXAFS spectrum as a function of electrochemical bias and with electroreduction time under in-situ electrochemical CO_2_ reduction conditions. Reproduced with permission from Ref. [127]. **f** Schematic illustration of the preparation procedure of Bi NSs. (purple, gray, orange, and yellow balls represent Bi, C, O, and H, respectively). **g** FEs and cathodic energetic efficiency (CEEs) of formic acid over two electrocatalysts in 1 M KOH. **h** Gibbs free energy profiles for CO_2_ electroreduction to HCOOH on Bi NPs and Bi NSs. Reproduced with permission from Ref. [128]

Bi is also a promising electrocatalyst for CO_2_RR to produce formate in aqueous solutions because of its large overpotential for HER in the aqueous electrolyte (Bi is situated at the bottom corner point of the volcano plot), low toxicity, and good stability [138, 139]. Therefore, Bi-based MOFs have drawn tremendous attention as catalysts for CO_2_ reduction to HCOOH [140]. Zhang et al. [126] synthesized a Bi-MOF (Bi-BTC-D) by the hydrothermal method and evaluated its CO_2_RR performance in the CO_2_-saturated 0.5 M KHCO_3_ electrolyte. Electrochemical experiments showed HCOOH selectivity with a maximum FE of 95.5% at -0.86 V *vs.* RHE. DFT calculations revealed that BTC ligands in MOF structure can effectively regulate the catalytic activity of the Bi atoms. Li et al. [127] also use the same ligand (H_3_BTC) to construct a helical rod-based 2D Bi-MOF (CAU-17) with permanent crystallographic-independent channels (Fig. 11a), and it exhibits high CO_2_-to-HCOOH activity in the H-cell. Such catalyst achieves an excellent FE of 92.2% at −0.9 V *vs.* RHE (Fig. 11b, c). It is worth noting that the type of catholyte significantly affects the selectivity of HCOOH (Fig. 11d). When SO_4_^2−^ was added in the catholyte, the accumulated negative charges on the electrode surface can generate a potential difference in the electric double layer and facilitates the transport of polar water molecules instead of nonpolar CO_2_ molecules, leading to the dominating competitive HER. Operando XAS (Fig. 11e) and DFT calculations were also conducted to explore the origin of the high HCOOH selectivity on the Bi-MOF, and the results showed that the highly accessible Bi^3+^ and the unique channels played vital roles in the enhancement of CO_2_ adsorption and HCOOH conversion. Nevertheless, the maximum HCOOH partial current density is only 15 mA cm^−1^ at −1.1 V *vs.* RHE, which is far from meeting the requirements for industrial applications (Fig. 11c). Yang et al. [128] also synthesized a Bi-MOF (CAU-17) with claviform shape and then spray on CP as the precursor, following an in-situ electroreduction process to fabricate leafy Bi nanosheets (Bi NSs) (Fig. 11f). Electrochemical experiments, which were conducted in a flow cell reactor, revealed that Bi NSs are excellent catalyst with high HCOOH activity and selectivity in both 1 M KHCO_3_ or KOH electrolytes. The maximum FE of Bi NSs is up to 98% at the potential of −0.48 V *vs.* RHE (total current density up to 133 mA cm^−2^) (Fig. 11g). Significantly, the HCOOH partial current density reaches 374 mA cm^−2^ at −1.51 V *vs.* RHE in 1 M KHCO_3_. The outstanding performance may be associated with the hybrid Bi/Bi-O species on the surface of Bi NSs. DFT calculations also confirm that O atoms of the Bi-O surface may be beneficial to reduce the free energy barrier for *OCHO formation (Fig. 11h).

**Fig. 12:**
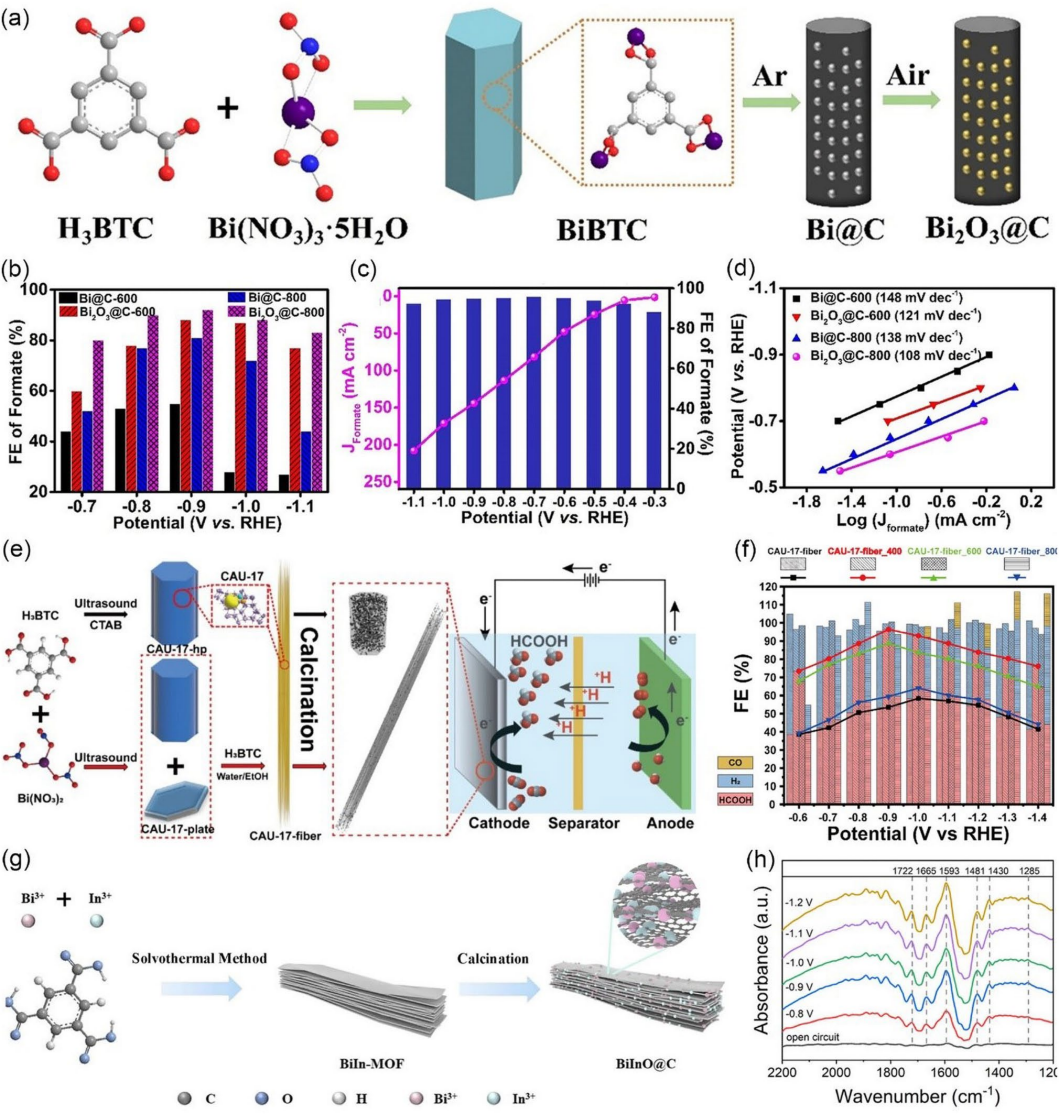
**a** Schematic illustration of the preparation procedure of Bi@C and Bi_2_O_3_@C catalysts. **b** FE of formate for various Bi-based composites in all potentials range. **c** FE and partial current density of HCOOH for Bi_2_O_3_@C-800 in all potentials range. **d** Tafel plots. Reproduced with permission from Ref. [129]. **e** Schematic representation of preparation of CAU-17-derived electrocatalysts. **f** CAU-17-fiber series at various potentials in CO_2_-saturated electrolyte based on a 2,500 s experiment. Reproduced with permission from Ref. [130]. **g** Schematic diagram of the preparation process of the BiInO-x@C catalyst. **h** In situ FT-IR spectra of BiInO -0.67@C at 1200 − 2200 cm^−1^. Reproduced with permission from Ref. [131]

Thanks to the tunable porosity and well-defined architectures, MOFs are particularly appealing with regard to fabricating versatile carbon hybrid nanostructures with well-defined compositions and morphologies. CAU-17, which is an ideal sacrificial template to fabricate various metal/carbon hybrids catalysts, has been widely used in CO_2_RR. For example, Deng et al. [129] construct carbon-nanorods-encapsulated bismuth oxides catalysts (Bi@C and Bi_2_O_3_@C) via the carbonization of CAU-17 in Ar and air atmosphere, respectively (Fig. 12a). The Bi_2_O_3_@C-800 shows a high FE_HCOOH_ of 92% at −0.9 V *vs.* RHE (Fig. 12b) in CO_2_-saturated 0.5 M KHCO_3_ electrolyte using an H-type reactor. However, the HCOOH partial current density is only 7.5 mA cm^−2^, which was attributed to the low solubility of CO_2_ in aqueous electrolytes. When they employ a flow cell configuration using 1 M KOH electrolyte, the HCOOH FE of Bi_2_O_3_@C-800 stays above 93% at −0.3 to −1.1 V *vs.* RHE and the HCOOH partial current density is significantly enhanced (Fig. 12c). The presence of crystalline Bi-O structure in the Bi_2_O_3_@C-800 is proved to be beneficial for promoting the reaction kinetics (Fig. 12d). The carbon matrix can significantly reduce the charge transfer resistance, which can promote the formation of *CO_2_^−^ intermediates. Therefore, the synergistic effect of Bi_2_O_3_ nanoparticles and carbon matrix is beneficial to improve the activity and selectivity for CO_2_-to-HCOOH. Ying et al. [130] prepared the CAU-17 fiber with a larger accessible surface area and abundant active catalytic sites via morphology engineering. Then, they calcined the fiber-shaped MOFs in an inert gas atmosphere to prepare Bi/C hybrids (CAU-17-fiber-x, x is the calcination temperature) for catalyzing CO_2_ to HCOOH (Fig. 12e). The CAU-17-fiber-400 gets the highest CO_2_-to-formate FE (96.4%), with a high partial current density (20.4 mA cm^−2^) at −0.9 V *vs.* RHE in CO_2_-saturated 0.1 M KHCO_3_ aqueous electrolyte using H-type cell (Fig. 12f). In the Bi/C hybrids, Bi nanoparticles (NPs) were encapsulated inside the CAU-17 derived porous fiber-shaped carbon framework, such a novel structure brought many unique benefits, such as larger accessible surface area and higher Bi content, thus improving the activity of CO_2_RR. Wang et al. [131] used another H_3_BTC ligand to prepare a Bi-based bimetallic MOF (BiIn-MOF), which was then calcined to obtain MOF-derived Bi/In bimetallic oxide nanoparticles/carbon (BiInO-x@C, x representative the ratio of Bi/In) in Ar atmosphere at 600 °C (Fig. 12g). Benefiting from the synergistic effect of bimetallic components, BiInO-0.67@C exhibits excellent activity and selectivity for the electroreduction of CO_2_ to formate. They also applied in-situ FT-IR to reveal the catalyst mechanism of BiInO-0.67@C. As shown in Fig. 12h, the peak at 1430 cm^−1^ is attributed to the symmetric stretching mode of OCO in the *HCOO species of the dioxygen bridge, indicating that the *HCOO pathway is the preferred route to produce formate on the catalyst.

**Fig. 13:**
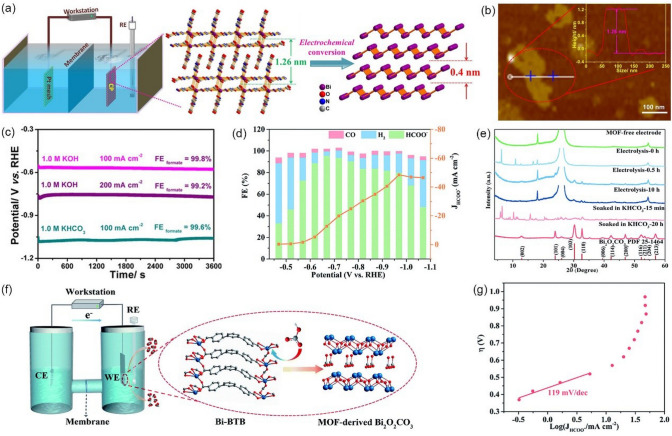
**a** Synthesis and characterizations of Bi-MOLs and Bi-ene. **b** Atomic force microscopy images of Bi-ene. **c** Chronopotentiometric curves at 100 and 200 mA cm.^−2^ in 1.0 M KHCO_3_ and KOH. Reproduced with permission from Ref. [132]. **d** FE of production and the current densities of HCOOH with Bi-BTB at different working potentials in CO_2_-saturated 0.5 M KHCO_3_ electrolyte. **e** The changes of XRD patterns for various samples. **f** Electrochemical cell for the electrolysis experiments and proposed mechanism for the formation of MOF-derived Bi_2_O_2_CO_3_. **g** Tafel plot for MOF-derived Bi_2_O_2_CO_3_ in CO_2_-saturated 0.5 M KHCO_3_ electrolyte. Reproduced with permission from Ref. [133]

In addition to carbon-based materials, MOFs can also be used to prepare ultrathin metallene materials. As demonstrated by Cao et al. [132], ultrathin Bi-based metal–organic layers could serve as a pre-catalyst to produce atomically thin bismuthene (Bi-ene) following in-situ electrochemical reconstruction (Fig. 13a). The as-obtained Bi-ene shows an average thickness of 1.28–1.45 nm (Fig. 13b) and exposes more active sites due to the two-dimensional nature. As a result, Bi-ene could deliver a FE_HCOOH_ close to 100% at a wide potential range in both KHCO_3_ and KOH electrolytes. Notably, the total current density can reach 200 mA cm^−2^ at −0.75 V *vs.* RHE in 1 M KOH (Fig. 13c). In-situ ATR-IR spectroscopy and DFT analysis confirmed that HCOOH is generated through the *HCOO intermediate on Bi-ene. Furthermore, Yuan et al. [133] prepared a Bi-1,3,5-tris(4-carboxy-phenyl) benzene (Bi-BTB) and discovered that the bismuth-carboxylate MOFs can be in-situ transformed to Bi_2_O_2_CO_3_ in an HCO_3_^−^ electrolyte. Bi-BTB exhibits an outstanding CO_2_-to-HCOOH performance (Fig. 13d). After electrolysis, the crystalline phase of Bi-BTB disappeared from the X-ray diffraction (XRD), while the peaks intensities of a new crystalline phase of Bi_2_O_2_CO_3_ dramatically increase, indicating that the true catalytic species are Bi_2_O_2_CO_3_ (Fig. 13e). Since Bi^3+^ ion and carboxylate belong to intermediate acid and hard base, Bi-BTB is not stable. Therefore, in the presence of HCO_3_^−^, the Bi–O bonds in Bi-BTB can be broken, resulting in the structural evolution from Bi-BTB to Bi_2_O_2_CO_3_ (Fig. 13f). A Tafel slope of 119 mV dec^−1^ for Bi_2_O_2_CO_3_ indicate that the initial electron transfer step is the RDS for the CO_2_RR (Fig. 13g). This work shows a good example of surface reconstruction and gives a strong signal that careful evaluation is required to distinguish the real reactive sites for different MOF electrocatalysts.

### Methane

Among all products derived from CO_2_RR, CH_4_ has attracted significant attentions because of its high values of mass heat (56 kJ g^−1^), good compatibility with current energy infrastructure [141–143]. Furthermore, the CO_2_RR reaction for CH_4_ formation is thermodynamically more favored than the reaction for both CO and HCOOH. However, as a deepest reduction product, the formation of CH_4_ involves eight electrons and sluggish kinetics, resulting in high overpotential and low selectivity. Therefore, it is extremely attractive to design catalysts with high activity and selectivity for CO_2_ reduction to CH_4_. To date, a number of MOF-related catalysts have been proved to be capable of promoting CO_2_RR toward CH_4_ (Table 4).

**Table 4:** Representative MOF-related catalysts for selectively catalyzing the electrochemical reduction of CO_2_ to CH_4_

Catalyst	MOF precursor	Electrolyte	FE (%)	Potential (V *vs.* RHE)	Refs.
Cu NPs	Cu-MOF-74	0.1 M KHCO_3_	~ 50	−1.3	[144]
N-Cu NPs	Cu^II^/ade-MOFs	0.1 M KHCO_3_	50	−1.6	[145]
Cu_2_O@Cu-MOFs	Cu-MOFs	0.1 M KHCO_3_	63.2	−1.71	[146]
Cu_2_O(111)@CuHHTP	CuHHTP	0.1 M KHCO_3_ + 0.1 M KCl	73	−1.4	[147]
Cu_4_^I^-MFU-4* l*	Cu_4_^II^-MFU-4* l*	0.5 M KHCO_3_	92	−1.2	[148]
Cu-based NNU-33(H)	–	1 M KOH	82.2	−0.9	[149]
Cu-DBC	–	1 M KOH	80	−0.9	[150]
HATNA-Cu-MOF	–	0.1 M KHCO_3_	78	−1.5	[151]
Cu(I)-MOFs (Cu-I)	–	1 M KOH	57.2	−1.08	[152]

**Fig. 14:**
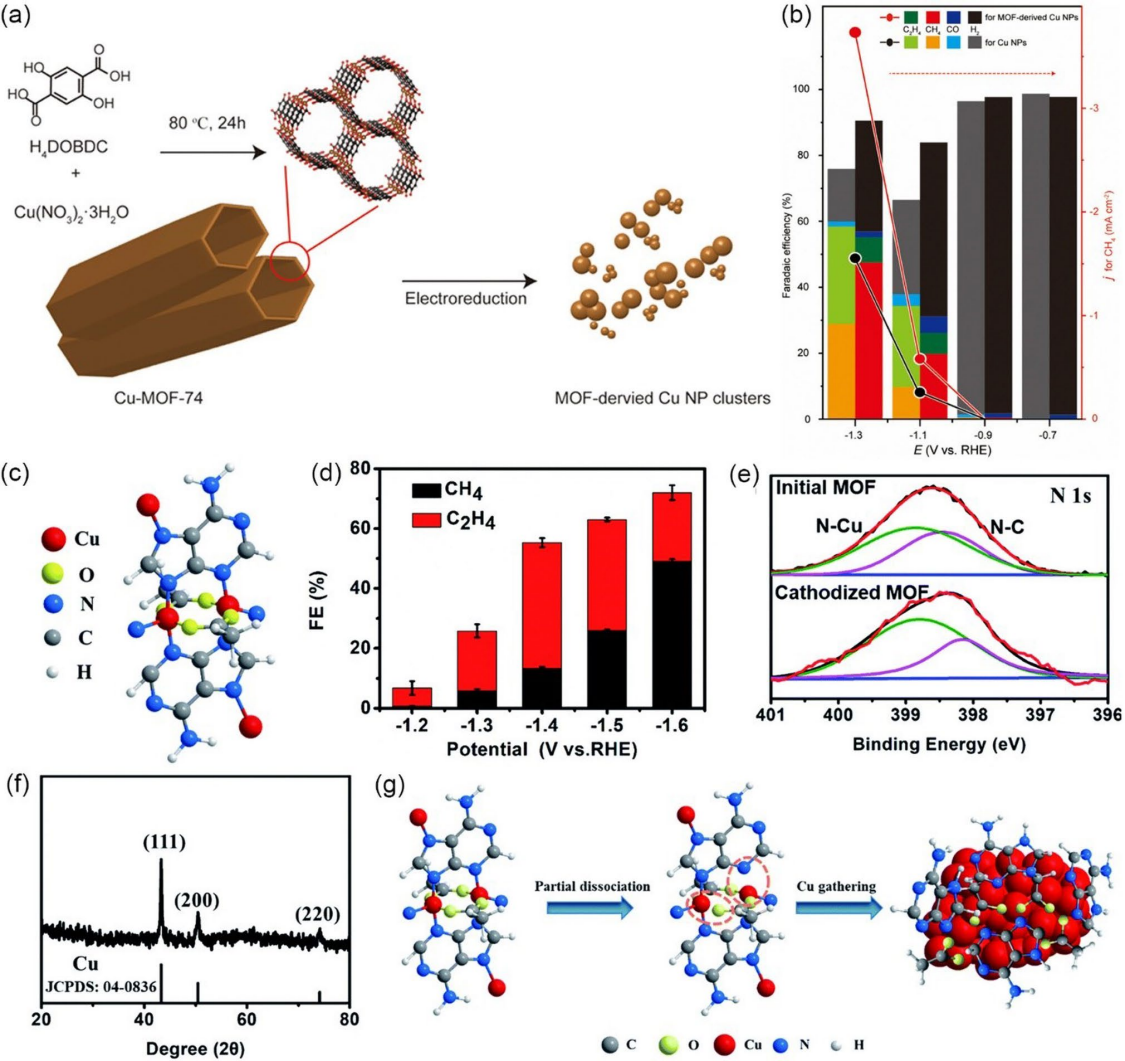
**a** Schematic illustration of the hydrothermal synthesis of Cu-MOF-74 and preparation of Cu NPs from Cu-MOF-74 by electroreduction. **b** Faradaic efficiencies for C_1_ and C_2_ hydrocarbons production and partial current density for CH_4_ production on Cu NP electrodes at the applied potential. Reproduced with permission from Ref. [144]. **c** Molecular structure of the Cu-ade monomer. **d** FE of CH_4_ and C_2_H_4_ for the s-Cu-ade MOF. **e** N 1*s* scan XPS patterns of the initial and cathodized Cu-ade MOFs. **f** XRD pattern of the cathodized s-Cu-ade MOF. **g** Proposed Cu-ade MOF evolution. Reproduced with permission from Ref. [145]

Generally, Cu-based catalysts show the best catalytic activity for the selective production of CH_4_ in the CO_2_RR system. For example, Kim et al. [144] prepared highly isolated Cu nanoparticles (Cu NPs) clusters with an average size of 30–50 nm for CO_2_-to-CH_4_, which were synthesized from Cu-MOF-74 precursor via an electroreduction process (Fig. 14a). When tested in 0.1 M KHCO_3_, the as-obtained Cu NPs clusters exhibited good catalytic activity toward CH_4_. As shown in Fig. 14b, the maximum FE of CH_4_ is approximately 50% at −1.3 V *vs.* RHE, while only 35% is achieved by commercial Cu NPs at the same conditions. It should be also noted that Cu NPs show the highest activity for CH_4_ at −1.3 V *vs.* RHE, and the CH_4_ partial current densities on Cu NPs are 2.3 times that of the commercial Cu NPs. It is believed that the activity difference is attributed to the extent of aggregation of Cu particles at nanoscales and MOF-derived Cu NPs are found to be less aggregated. Yang et al. [145] employed adenine and acetic acid ligands to fabricate Cu-ade MOF with different thicknesses (Cu-ade nanosheets (s-Cu-ade), nanoplates (p-Cu-ade) and nanocuboids (c-Cu-ade)). Figure 14c shows the molecular structure of the Cu-ade monomer, where stable MOFs were formed by the bonding of N with C and Cu. Electrochemical measurements demonstrated that s-Cu-ade has the best CO_2_ reduction performance in CO_2_-saturated 0.1 M KHCO_3_ electrolyte and the maximum FE of CH_4_ is over 50% at -1.6 V vs. RHE (Fig. 14d). It worth noting that the CO_2_ electroreduction process can induce the structure evolution of the Cu-MOF to form Cu nanoparticles functionalized by the nitrogen containing ligands (Fig. 14e, f). The presence of N-containing functional groups would activate the protons to obtain COH* or CHO* intermediate [153–156], which are critical intermediates for further hydrogenation to form CH_4_, thus boosting the conversion of CO_2_ to CH_4_ (Fig. 14g).

**Fig. 15:**
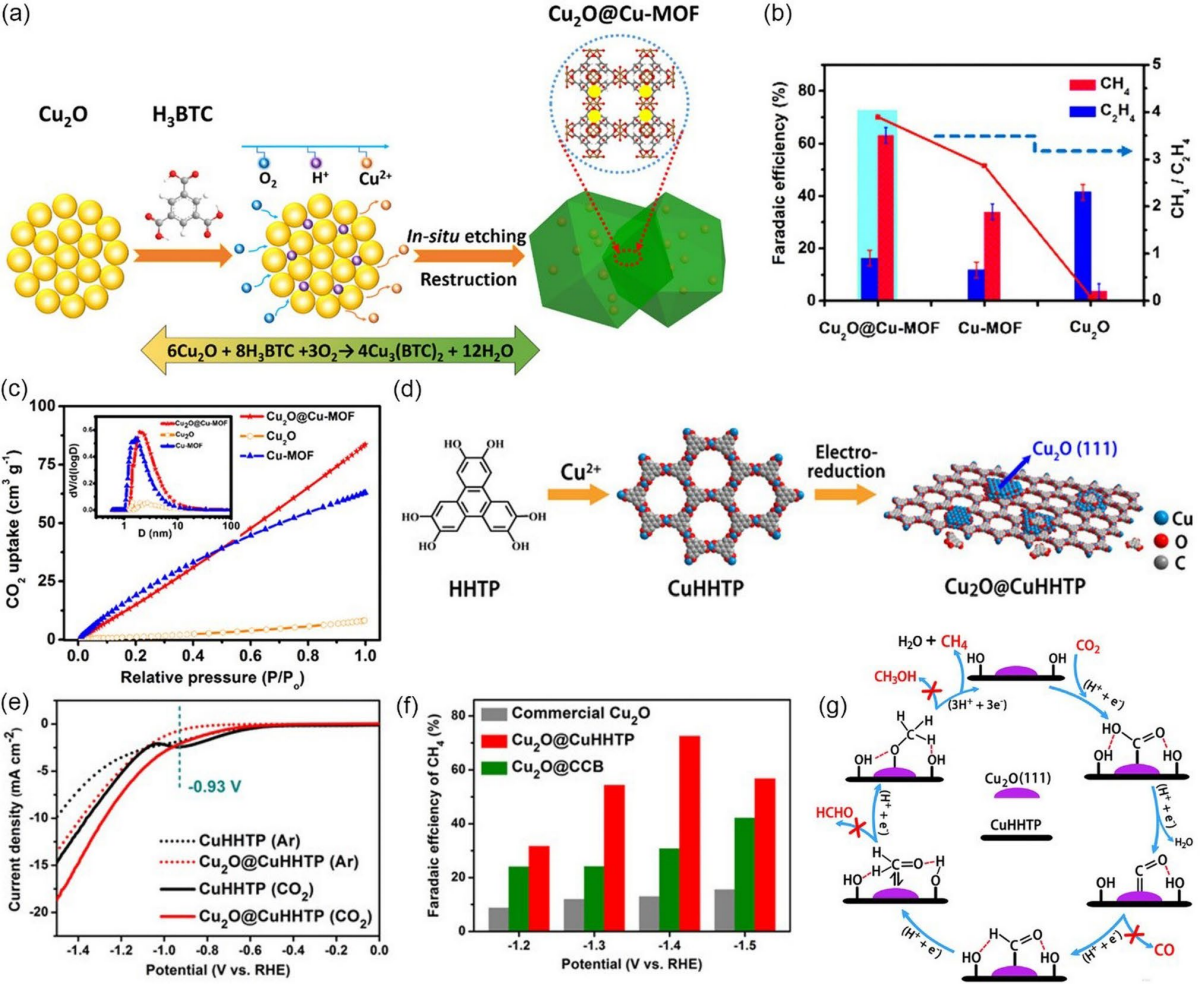
**a** Schematic illustration of the process to synthesize Cu_2_O@Cu-MOF. **b** FEs of CH_4_ and C_2_H_4_ and the ratio of CH_4_ to C_2_H_4_ for Cu_2_O@Cu-MOF, Cu-MOF, and Cu_2_O at − 1.71 V versus RHE in CO_2_-saturated 0.1 M KHCO_3_ solution. **c** CO_2_ adsorption curves of Cu_2_O@Cu-MOF, Cu-MOF, and Cu_2_O. Reproduced with permission from Ref. [146]. **d** Illustration of the solvothermal synthesis of CuHHTP and preparation of Cu_2_O@CuHHTP via electrochemical treatment of CuHHTP at the applied potential of -1.2 V vs. RHE for 30 min. **e** LSV curves of CuHHTP and Cu_2_O@CuHHTP in 0.1 M KCl/0.1 M KHCO_3_ electrolyte under Ar and CO_2_. **f** Comparison of CH_4_ FE between Cu_2_O@CuHHTP, Cu_2_O on conductive carbon black(Cu_2_O@CCB), and commercial Cu_2_O. **g** Proposed mechanism of Cu_2_O@CuHHTP for the formation of CH_4_. Reproduced with permission from Ref. [147]

As discussed above, achieving high hydrocarbon selectivity remains a great challenge. Fortunately, the synergistic strategy that combines Cu_2_O with Cu MOFs seems to be an effective way to enhance hydrocarbon selectivity, which has been confirmed by recent studies. For instance, Tan et al. [146] prepared an all-in-one hybrid Cu_2_O@Cu-MOF by time-resolved controllable restructuration. Briefly, the surface of Cu_2_O spheres can be oxidized to Cu^2+^ in the mixed alcohol solution at 80 °C, and then Cu^2+^ can further coordinate with H_3_BTC to form Cu-MOFs on the surface of Cu_2_O, resulting in Cu_2_O@Cu-MOF (Fig. 15a). When tested in CO_2_-saturated 0.1 M KHCO_3_ solution, Cu_2_O@Cu-MOF showed considerable FE_CH4_, which was significantly higher than that of both Cu_2_O and Cu-MOF, and the maximum FE was up to 63.2% at −1.71 V vs. RHE (Fig. 15b). Due to the porous nature of the framework, Cu_2_O@Cu-MOF exhibited considerable adsorption capacity of CO_2_ molecules (Fig. 15c), which significantly enlarge the local CO_2_ concentration on the active sites of the electrode, while the intrinsic catalytic activity of Cu_2_O can be maintained well simultaneously. Moreover, the Cu_2_O core embedded in the Cu-MOF accelerates charge transfer. On the contrary, Yi et al. [147] fabricated Cu_2_O(111) quantum dots with an average size of 3.5 nm on a porous conductive Cu-MOFs (CuHHTP) via an electroreduction process (Fig. 15d). Linear sweep voltammetry (LSV) tests are conducted in both Ar and CO_2_-saturated 0.1 M KCl + 0.1 M KHCO_3_ mixture solution. As shown in Fig. 15e, compared with pristine Cu-MOFs, the Cu_2_O(111)@CuHHTP shows larger current densities at same conditions, implying that Cu_2_O(111) quantum dots have high electrocatalysis activity for the CO_2_RR. Notably, Cu_2_O(111)@CuHHTP achieved high selectivity of 73% at -1.4 V vs. RHE toward CH_4_ with partial current density up to -10.8 mA cm^−2^ (Fig. 15f). The superior electrochemical performance was attributed to the following two reasons: 1) Due to the strong charge delocalization between Cu^2+^ and HHTP, Cu-MOFs shows excellent electronic conductivity (5.1 × 10^–5^ S m^−1^) and serves as a conductive substrate, which can accelerate the electron transfer to Cu_2_O(111) during the CO_2_RR process. 2) During electroreduction, Cu-O_4_ nodes in Cu-MOFs were partially reduced to Cu_2_O, thus exposing a number of hydroxyl groups of the uncoordinated HHTP ligand. The hydroxyl-rich environment was assumed to stabilize the *CO intermediate by hydrogen bonding (Fig. 15g) and improves the selectivity toward CH_4_ [157–159].

**Fig. 16:**
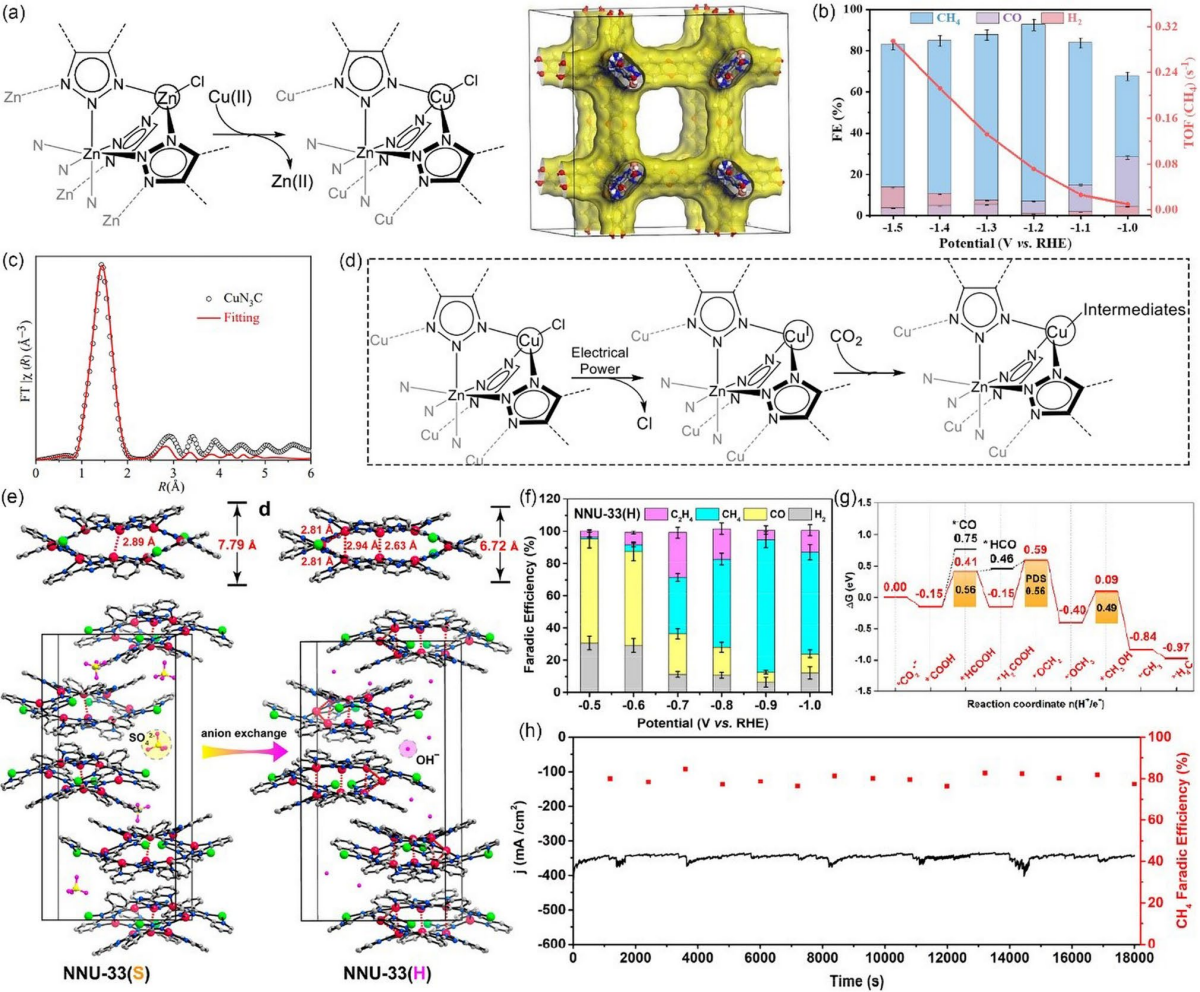
**a** Structures of local coordination environments, metal transformation process, and Cu_4_^II^-MFU-4 l (3D channel surface highlighted in yellow). **b** FE and TOF by Cu_4_^II^-MFU-4 l. **c** Cu K-edge EXAFS spectra and fitting for CuN_3_C at -1.2 V under CO_2_. **d** Illustration of the conversion from Cu(II) to Cu(I) ions and the formation of intermediates during the electrocatalysis. Reproduced with permission from Ref. [148]. **e** Structures of {Cu_8_} clusters and unit cell in NNU-33(S) and NNU-33(H), respectively. **f** Electrocatalytic performances of NNU-33(H) FE of H_2_, CO, CH_4_, and C_2_H_4_ products. **g** Calculated free energy diagram and the corresponding intermediates for CO_2_ electrocatalytic reduction to CH_4_ on the Cu_8_ model catalyst. **h** Current profile and FEs of CH_4_ at a constant voltage of -0.9 V vs RHE. Reproduced with permission from Ref. [149]

Apart from Cu_2_O, many other Cu(I)-based catalysts have also been demonstrated to be active for the selective reduction of CO_2_ to CH_4_. For example, Zhu et al. [148] prepared a Cu-MOF, Cu_4_ZnCl_4_(btdd)_3_ (Cu_4_^II^-MFU-4* l*, H_2_btdd = bis(1H-1,2,3-triazolo-[4,5-b],[4′,5′-i])dibenzo-[1, 4]-dioxin), by an ion exchange process that uses Cu(II) ions to replace outer sphere Zn(II) ions in Zn_5_Cl_4_(btdd)_3_ (MFU-4* l*) cluster (Fig. 16a). When tested in CO_2_-saturated 0.5 M NaHCO_3_ solution, Cu_4_^II^-MFU-4* l* showed obvious electrocatalytic CO_2_RR activity and achieved a high CH_4_ FE of 92% at -1.2 V vs. RHE (Fig. 16b), while MFU-4* l* only yielded minor CO product at the same condition. After long-term electrolysis, the Cu(II) ions were found to be reduced to Cu(I) ions (Cu_4_Zn-(btdd)_3_) with the trigonal pyramidal Cu(I)N_3_ sites, indicating that the Cu(I)N_3_ sites is the actual active centers for the CO_2_RR (Fig. 16c). Figure 16d shows the conversion process from Cu(II) to Cu(I) ions during the electrocatalysis. Moreover, in situ experiments and DFT calculations revealed that the synergistic interactions between Cu(I)N_3_ sites and adjacent aromatic hydrogen atoms can stabilize the key CO_2_-to-CH_4_ intermediates via hydrogen bonding. In order to understand the effect of intrinsic cuprophilic interactions inside the Cu(I) catalysts, Zhang et al. [149] used 2-(5-(3-(5-(pyridin-2-yl)-1H-,2,4-triazol-3-yl)phenyl)-1H-1,2,4-triazol-3-yl) pyridine (H_2_bptb) and Cu(I) ions to construct two MOFs (NNU-32 and NNU-33(S) (S = sulfate radical)). Then they transformed NNU-33(S) into NNU-33(H) (H = hydroxyl) through an anion exchange process in 1 M KOH solution (Fig. 16e). After structural transformation, NNU-33(H) showed enhanced cuprophilic interactions and expanded interlayer distances. The electrocatalytic CO_2_RR performance was tested in 1 M KOH solution using a flow cell and the results showed that NNU-33(H) exhibited a high FE_CH4_ value of 82.17% at -0.9 V vs. RHE (Fig. 16f). Significantly, the current density is up to 391.79 mA cm^−2^ at -0.9 V vs. RHE, which is sufficient to satisfy the requirements of industrial applications. The XAS, in situ Raman, XPS, and in situ FTIR were performed to confirm the structural stability of NNU-33(H) and those results suggested that the properties of NNU-33(H) are stable during CO_2_RR. The distances of Cu(I)-Cu(I) in both NNU-32 and NNU-33(H) are a little larger than twice the covalent radius of Cu but significantly shorter than twice its van der Waals radius, suggesting the existence of cuprophilic interactions in the crystal. Moreover, the distances of Cu ions in NNU-33(H) are shorter than that NNU-32, illustrating stronger cuprophilic interactions in NNU-33(H), which may be the key factor that influences the CH_4_ selectivity. DFT calculations revealed the fourth hydrogenation step (*H_2_COOH → *OCH_2_) is the potential determining step (PDS) (Fig. 16g). Significantly, the free energy of the PDS process is increased significantly from 0.74 to 1.11 eV after removing the Cu(I)-Cu(I) interaction, confirming that the internal Cu-Cu interaction plays an essential role in the CO_2_RR process. However, the durability of NNU-33(H) still needs to be improved (Fig. 16h).

**Fig. 17:**
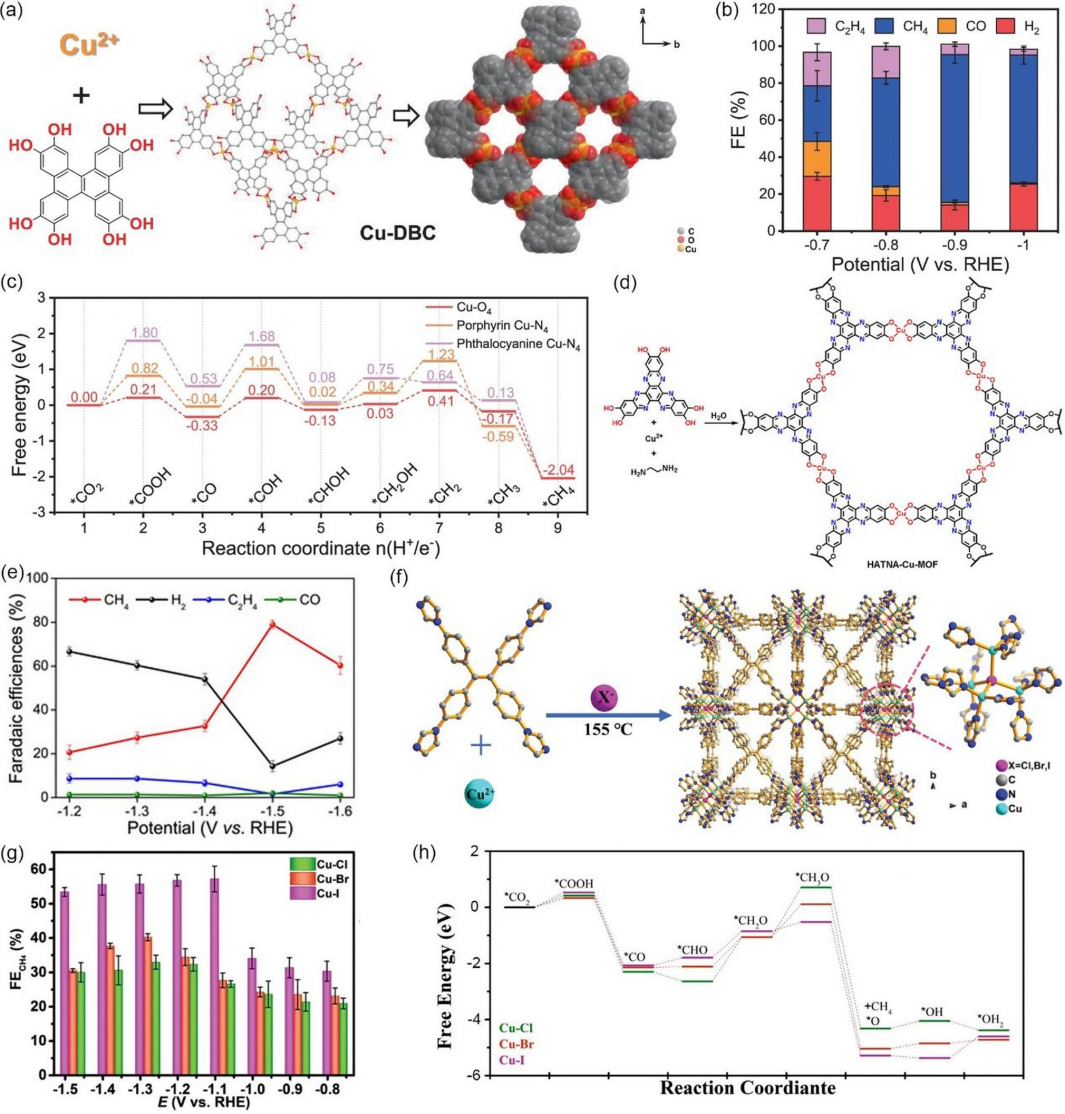
**a** Structure obtained by Cu ions and 8OH-DBC. **b** FEs of CO_2_RR products at different applied potentials. **c** Free energy profiles for the CO_2_RR-to-CH_4_ reaction pathway. Reproduced with permission from Ref. [150]. **d** Synthesis of HATNA-Cu-MOF. **e** Potential dependent FE of different reduction products. Reproduced with permission from Ref. [151]. **f** Schematic illustration of the synthesis and structure of Cu-X. **g** Average FEs of CH_4_ at different potentials over Cu-Cl, Cu-Br, and Cu-I catalysts. **h** Gibbs free energy profiles of CO_2_ reduction reaction on Cu-Cl, Cu-Br, and Cu-I. Reproduced with permission from Ref. [152]

As discussed before, reasonable regulation of the Cu coordination environment in MOFs is a common method to modulate the selectivity of electroreduction catalysis, this is also applicable for the CO_2_-to-CH_4_ process using MOF-based electrocatalysts. For example, Zhang et al. [150] employed the highly conjugated organic ligand (dibenzo-[g,p] chrysene-2,3,6,7,10,11,14,15-octaol, 8OH-DBC) to construct a Cu-based conductive MOFs (Cu- DBC) with abundant and uniformly distributed Cu-O_4_ sites (Fig. 17a). Cu-DBC exhibits an electrical conductivity of 1.2 × 10^–2^ S m^−1^ due to the charge delocalization between metal ions and conjugated ligands. Electrochemical experiments reveal that Cu-DBC delivers obvious CO_2_RR activity with a maximum CH_4_ FE of 80% at -0.9 V vs. RHE (Fig. 17b). The experimental measurements and DFT calculations further revealed that the Cu-O_4_ site in Cu-DBC is easier to be reduced into low-valence Cu sites during the activation process and is more energetically favorable for the following CO_2_ reduction compared to nitrogen-coordinated Cu sites (Fig. 17c). Liu et al. [151] designed a conjugated, nitrogen-containing ligand hexahydroxyl-hexaazatrinaphthylene (HATNA-6OH) and further synthesized 2D conductive MOFs (Cu_3_(C_24_H_6_O_6_N_6_)_2_ 1.5(NH_3_CH_2_CH_2_NH_3_), HATNA-Cu-MOF) by a solvothermal method in the presence of ethylenediamine (Fig. 17d). Owing to the synergic effects of the redox active copper catecholate nodes and favorable p-p stacking of the ligand, HATNA-Cu-MOF exhibits high CH_4_ selectivity with a FE of 78% at -1.5 V vs. RHE (Fig. 17e). Unfortunately, the CH_4_ partial current density is only -8.2 mA cm^−2^. In addition, Zhang et al. [152] prepared a series of Cu_4_X cluster-based MOFs ([Cu_4_X(TIPE)_3_]·3X, [X = Cl, Br, I, TIPE = 1,1,2,2-tetrakis(4-(imidazol-1-yl)phenyl)ethene], named as Cu-Cl, Cu-Br, Cu-I) to investigate the effect of different halogens atoms on the activity and selectivity of CO_2_RR products (Fig. 17f). Electrochemical experiments revealed that Cu-I is the optimal catalyst for CO_2_ reduction to CH_4_ with the highest FE_CH4_ of 57.2% at −1.08 V vs. RHE (Fig. 17g). Meanwhile, the CH_4_ partial current density is up to 60.7 mA cm^−2^ at the same potential. In order to explore the origin of the high activity and selectivity of Cu-I, DFT calculations was also conducted. As shown in Fig. 17h, when the radius of halogen atom increase, the d-band center of Cu site is shifting closer to the Fermi level, which can significantly reduce the formation energy of the PDS and promote subsequent CH_4_ formation.

### Methanol

CH_3_OH is one of the important chemicals in the production of organic compounds and synthetic gasoline [160]. Moreover, CH_3_OH is a promising liquid fuel to replace fossil fuels because of its environmental friendliness and ease of transportation [161, 162]. CH_3_OH can also be directly used in conventional internal combustion engines or in direct methanol fuel cells, making it stand out as the alternative fuel for building a sustainable society [163]. Recently, the catalytic CO_2_ hydrogenation to CH_3_OH by MOF-related materials has been investigated and several representative examples are listed in Table 5.

**Table 5:** Representative MOF-related catalysts for selectively catalyzing the electrochemical reduction of CO_2_ to CH_3_OH

Catalyst	MOF precursor	Electrolyte	FE (%)	Potential (V *vs.* RHE)	Refs.
OD Cu/C-1000	Cu-BTC	0.1 M KHCO_3_	43.2	−0.3	[164]
Cu@Cu_2_O-400	Cu-BTC	0.5 M KHCO_3_	45	−0.7	[165]
Pt_x_Zn/C (1 < x < 3)	ZIF-8	0.1 M NaHCO_3_	81.4	−0.9	[166]
CuSAs/TCNFs	ZIF-8	0.1 M KHCO_3_	44	−0.9	[167]
Cu_3_(HHTQ)_2_	–	0.1 M KHCO_3_	53.6	−0.4	[168]

**Fig. 18:**
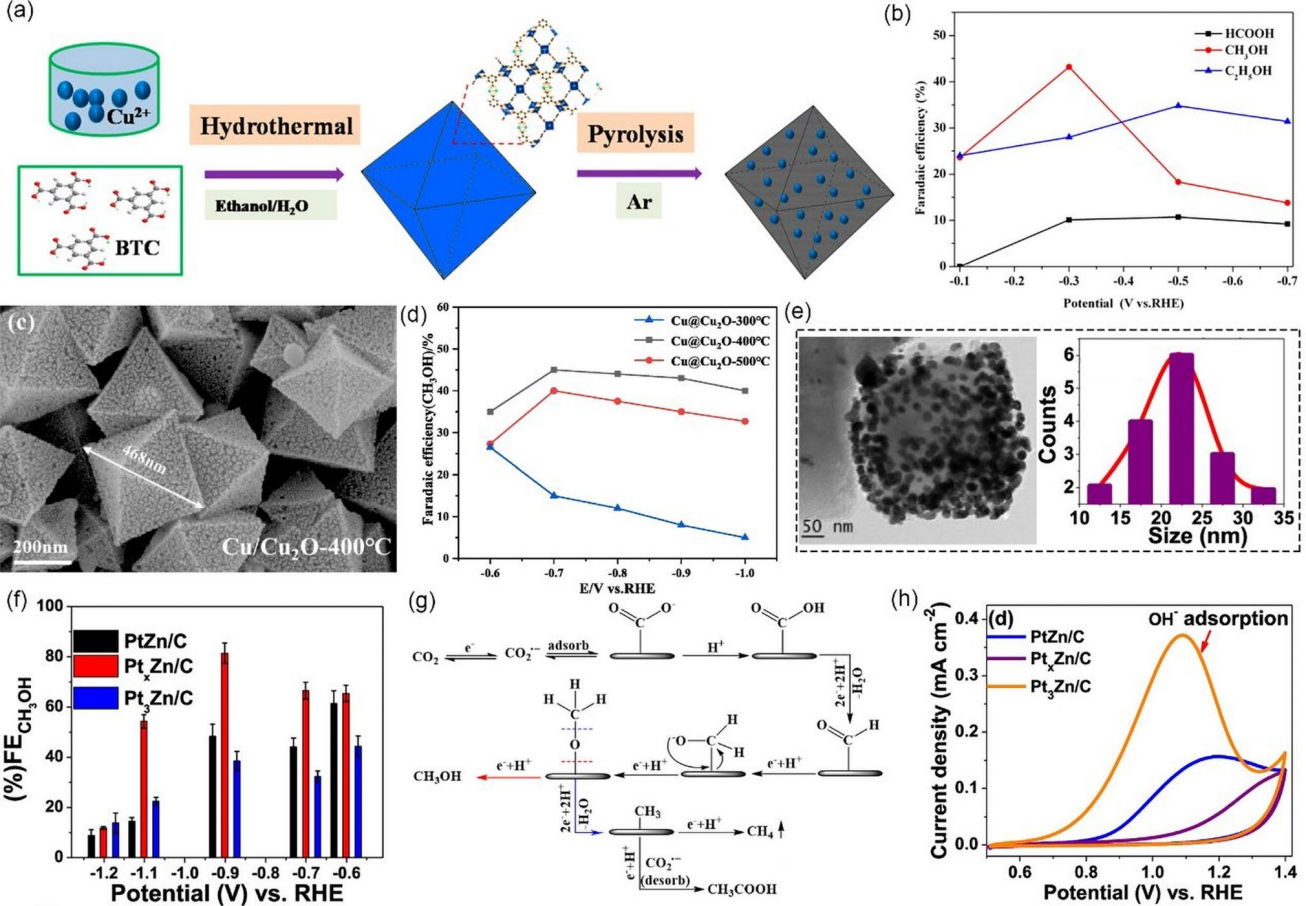
**a** Synthesis process of oxide-derived Cu/Carbon catalysts. **b** EE for CO_2_ electrochemical reduction of OD Cu/C-1000. Reproduced with permission from Ref. [164]. **c** Scanning electron microscope (SEM) images of Cu@Cu_2_O electrocatalysts derived from Cu-BTC pyrolysis at 400 °C. **d** CH_3_OH over Cu@Cu_2_O-T electrocatalysts at various applied potentials in CO_2_-saturated 0.5 M KHCO_3_. Reproduced with permission from Ref. [165]. **e** High resolution transmission electron microscopy (HR-TEM) images of PtxZn/C and corresponding particle size distribution. **f** FE of CH_3_OH in CO_2_RR over the intermetallic nano-alloys as a function of potentials. **g** Possible mechanism of CO_2_RR with product distribution over intermetallic nano-alloys. **h** CV traces for hydroxide adsorption in 0.1 M NaOH solution over the intermetallic nano-alloys. Reproduced with permission from Ref. [166]

Two impressive studies, which employed Cu-BTC as the precursor to fabricate catalysts with high selectivity for CH_3_OH were reported. In one study, Zhao et al. [164] carbonized Cu-BTC to synthesize oxide-derived Cu nanoparticles in a porous carbon matrix (OD Cu/C) under Ar atmosphere (Fig. 18a). Subsequently, they loaded OD Cu/C catalysts on CP as a working electrode and tested its CO_2_RR performance in CO_2_-saturated 0.1 M KHCO_3_ solution. As shown in Fig. 18b, OD Cu/C-1000 (1000 is the carbonization temperature) exhibits high selectivity and activity for CO_2_ reduction to CH_3_OH, with a maximum FE of 43.2% at −0.3 V vs. RHE. Significantly, the overpotential for CH_3_OH formation is only 190 mV. The synergistic effect between the highly dispersed copper and the porous carbon is beneficial for converting the adsorbed CO to alcohol, thus improving the activity and selectivity for CH_3_OH. Furthermore, the existence of a carbon matrix can protect the active sites' deactivation during the electrochemical reduction of CO_2_, thus enhancing the durability of OD Cu/C-1000. Another study was reported by Yang et al. [165]. Briefly, the PVP-modified Cu-BTC was synthesized by hydrothermal method and then calcined in air to obtain a carbon-supported Cu@Cu_2_O catalyst (Fig. 18c). Three samples were calcined at different temperatures, and Cu@Cu_2_O−400 ℃ was found to show distinct activity for catalyzing CO_2_ to CH_3_OH. The maximum FE_CH3OH_ was up to 45% at −0.7 V vs. RHE and continuous electrolysis for 2 h was demonstrated in CO_2_-saturated 0.5 M KHCO_3_ aqueous solution (Fig. 18d). Compared with other samples, Cu@Cu_2_O-400℃ has the highest concentration of Cu^+^, which could not only enhance the absorption capacity of the CO* intermediate but also promote its protonation on C site to form CHO*. Furthermore, the synergistic effect between Cu^0^ and Cu^+^ could adjust CO* binding energy, and the surface OH groups could help to increase the concentration of the *H, both facilitating the CH_3_OH formation following the protonation or CPET process. These conclusions are also confirmed by in-situ ATR-IR measurements. In addition, Payra et al. [166] also prepared carbon-supported intermetallic alloys as electrocatalysts toward CO_2_-to-CH_3_OH. Intermetallic PtZn/C, Pt_3_Zn/C, and Pt_x_Zn/C (1 < x < 3) NPs on N-doped carbon were synthesized by thermal decomposition of ZIF-8 under an inert atmosphere (Fig. 18e). Three synthesized intermetallic nano-alloys were deposited on glassy carbon electrode and tested in CO_2_-saturated 0.1 M NaHCO_3_ solution. The mixed-phase Pt_x_Zn/C was found to show the highest FE of up to 81.4% at −0.9 V vs. RHE (Fig. 18f). Figure 18g shows the possible reaction paths for CO_2_ reduction over the intermetallic nano-alloys and the CH_3_OH selectivity is governed by the bonding strength of the surface *–OCH_3_ species. A weak interaction between O and the catalysts surface could facilitate desorption of the whole *–OCH_3_ group, and thus increasing the selectivity of CH_3_OH. Theoretical calculations revealed that Pt_x_Zn has the lowest bonding energies of *OCH_3_ compared to the other nano-alloys, which was also confirmed by the results of *–OH adsorption strength on the surface of the catalytic electrode (Fig. 18h).

**Fig. 19:**
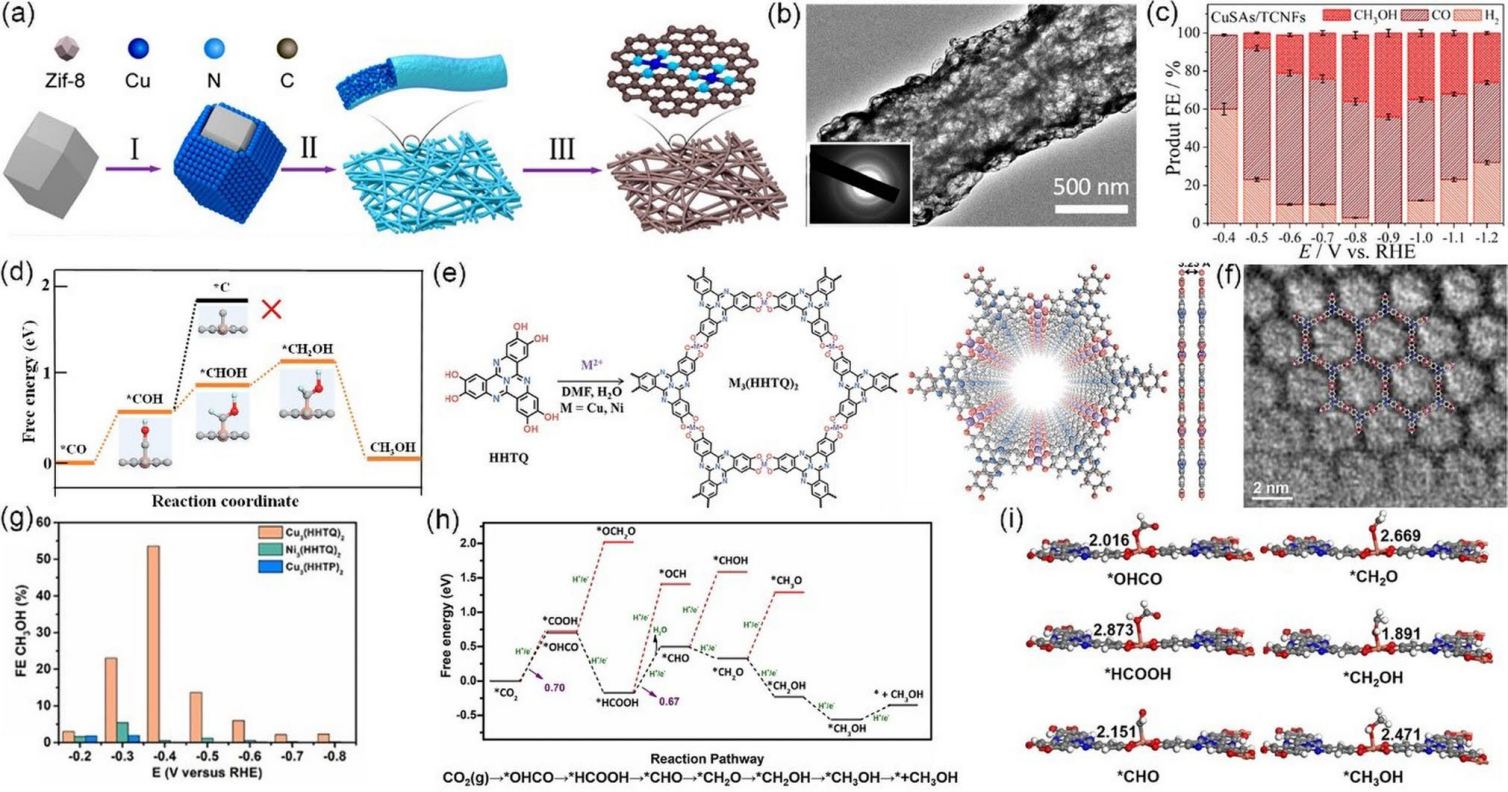
**a** Synthesis procedure of CuSAs/THCF: I, adsorption of Cu ions; II, electrospinning of polymer fibers; III, carbonization and etching. **b** HR-TEM images of CuSAs/TCNFs; the inset of **b** shows the SAED pattern. **c** FEs of all products at CuSAs/TCNFs. **d** Free energies for conversion of *CO to CH_3_OH on Cu-N_4_ structure. Orange, gray, dark blue, red, and light blue spheres stand for Cu, C, N, O, and H atoms, respectively. Reproduced with permission from Ref. [167]. **e** Synthesis of M_3_(HHTQ)_2_ (M = Cu, Ni), C gray, N blue, O red, Cu purple, H white spheres. **f** Zoom-in view of HR-TEM image for Cu_3_(HHTQ)_2_ taken along the c axis that shows a hexagonal pore and ligand termination, overlaid with a structure model. **g** CH_3_OH FEs for Cu_3_(HHTQ)_2_, Ni_3_(HHTQ)_2_, Cu_3_(HHTP)_2_ at different potentials. **h** Free energy profiles for the CO_2_RR on Cu_3_(HHTQ)_2_ and the proposed catalytic mechanism for electrochemical reduction of CO_2_ to CH_3_OH by CuO_4_ sites of Cu_3_(HHTQ)_2_. **i** Structures of the catalyst and key reaction intermediates involved in the proposed reaction mechanism for the CO_2_RR on Cu_3_(HHTQ)_2_. Reproduced with permission from Ref. [168]

Downsizing the active metal component to the atomic level is also a promising way to improve the CH_3_OH selectivity. For example, Yang et al. [167] dispersed numerous isolated Cu atoms in through-hole carbon nanofibers to synthesize flexible and self-supported single-atom catalysts (CuSAs/TCNFs) with high CO_2_-to-CH_3_OH efficiency. Briefly, they embedded Cu/ZIF-8 precursor into PAN nanofiber and then carbonize it to form the isolated Cu atoms (Fig. 19a). Notably, no metal clusters or nanoparticles are found in the CuSAs/TCNFs (Fig. 19b). Thanks to the good mechanical strength and high conductivity, CuSAs/TCNFs can be directly used as cathodes without binder or current collector. As shown in Fig. 19c, due to the synergetic effect of the both through-hole carbon structure and abundant isolated Cu active sites, the FEs for CH_3_OH reached a maximum value of 44% at -0.9 V vs. RHE when tested in CO_2_-saturated 0.1 M KHCO_3_ electrolyte. DFT calculation showed that the Gibbs free energy for *COH to *CHOH (~ 0.86 eV) on the active sites (Cu-N_4_) is significantly lower than that for *COH to *C (~ 1.88 eV), thus CH_3_OH instead of CH_4_ is more thermodynamically favored (Fig. 19d). In addition, Liu et al. [168] also reported a honeycomb-like single-atom catalyst (M_3_(HHTQ)_2_, M = Cu, Ni; HHTQ = C_3_-symmetric 2,3,7,8,12,13-hexahydroxytricyclo-quinazoline), where the Cu or Ni atoms are uniformly anchored in the hexagonal lattices (Fig. 19e, f). The content of metal atoms was up to 20% in the M_3_(HHTQ)_2_ and the interplay between both metal centers and nitrogen-rich organic ligands in Cu_3_(HHTQ)_2_ significantly improved the CO_2_-to-CH_3_OH selectivity, showing a FE up to 53.6% at a low overpotential of 0.4 V (Fig. 19g). DFT calculations were also conducted to reveal the catalyst mechanism of Cu_3_(HHTQ)_2_ and the most favorable reacting pathway was proposed as follows: CO_2_(g) → *OHCO → *HCOOH → *CHO → *CH_2_O → *CH_2_OH → *CH_3_OH → * + CH_3_OH (Fig. 19h, i). This work provides new insights into designing novel 2D conductive MOFs as electrocatalysts for CO_2_RR by enhancing the interplay between metal centers and organic ligands.

### Multi-carbon (C_2+_) Species

C_2+_ chemicals such as C_2_H_4_ and CH_3_CH_2_OH, are attractive and desirable high-energy–density CO_2_RR products [169–171]. However, it is still challenging to produce C_2+_ chemicals through CO_2_RR because of the high energy barrier and low selectivity for C–C coupling in aqueous electrolytes [172]. MOF-related materials provide a promising way for catalyzing CO_2_ to C_2+_ products, several representative examples are listed in Table 6.

**Table 6:** Representative MOF-related catalysts for selectively CO_2_RR toward C_2+_ products

Catalyst	MOF precursor	Main product	Electrolyte	FE (%)	Potential (V *vs.* RHE)	Refs.
Cu GNC-VL	ZIF-L	C_2_H_5_OH	0.5 M KHCO_3_	70.52	−0.87	[173]
Cu(111)@Cu-THQ	Cu-THQ	C_2_H_4_	0.1 M KHCO_3_	44.2	−1.2	[174]
HKUST-1_15_	HKUST-1	CH_3_CH_2_OH + C_2_H_4_	0.1 M KHCO_3_	58.6	−0.98	[175]
Cu nanoribbons	Cu-MOFs	CH_3_CH_2_OH + C_2_H_4_	1 M KOH	82.3	−1.23	[176]
PcCu-Cu–O	–	C_2_H_4_	0.1 M KHCO_3_	50	−1.2	[177]
S-HKUST-1	HKUST-1	C_2_H_4_	1 M KOH	57.2	−1.2	[178]
AuNP@PCN-222(Cu)	PCN-222(Cu)	C_2_H_4_	0.1 M KHCO_3_	52.5	−1.2	[179]
Cu_0.5_NC	ZIF-8	CH_3_CH_2_OH	0.1 M CsHCO_3_	55	−1.2	[180]
Cu-SA/NPC	ZIF-8	CH_3_COCH_3_	0.1 M KHCO_3_	36.7	−0.36	[181]

Cu-based MOF-related catalysts exhibit remarkable activity and selectivity for the production of C_2+_ chemicals in CO_2_RR because of their moderate binding energy to *CO intermediates, which is critical for the formation of C–C coupling. Zhang et al. [173] designed a 3D Cu-base catalyst (Cu GNC-VL) by introducing copper precursors into the oriented ZIF-L-derived GO nanosheets (vZIF-L@GO). Amorphous Cu/Cu_2_O NPs are found to uniformly disperse inside the Cu GNC-VL catalyst and facilitates electron transport. The synergistic effect between Cu (111) and Cu_2_O (111) not only enhances the CO_2_ adsorption but also facilitates the C–C coupling, thus resulting in a high CH_3_CH_2_OH FE of 70.52% and a total current density of 10.4 mA cm^−2^ at -0.87 V vs. RHE. As mentioned above (Sect. 4.1), Cu-THQ exhibits excellent performance for the electroreduction of CO_2_ to CO, thus creating a high local concentration of *CO on the catalyst surface. Zhao et al. [174] borrowed this idea and prepared a tandem catalyst (Cu(111)@Cu-THQ) via in-situ electroreduction. The integration of two kinds of active catalytic sites increased the *CO intermediate coverage on the Cu surface and also reduced the C–C coupling energy barrier. Owing to the synergistic effect of two active sites (Cu(111) and CuO_4_ nodes), such catalyst delivers a high C_2_H_4_ FE of up to 44.2% at -1.2 V vs. RHE in CO_2_-saturated 0.1 M KHCO_3_ electrolyte. Han et al. [175] prepared an HKUST-1 thin film to investigate the relationship between the structural evolution of these materials and product selectivity during continuous electrolysis. Electrochemical experiments showed that the catalytic activity of HKUST-1 varies with electrolysis time. After continuous electrolysis for 15 min, HKUST-1 was found to evolve into 3D nanospheres made from numerous small fragments (HKUST-1_15_). When electrolysis time increased to 120 min, HKUST-1 was thoroughly transformed into cross-linked nanobelts (HKUST-1_120_). Compared with HKUST-1_120_, HKUST-1_15_ had a higher Cu^+^/Cu ratio on the surface, which greatly promotes CO_2_ activation and facilitates the C–C coupling, thus exhibiting higher activity for CO_2_ reduction to CH_3_CH_2_OH and C_2_H_4_. Another study reported Cu-MOF-derived mesoporous Cu nanoribbons by a similar in-situ electrochemical reduction method [176]. The inherited mesoporous structure of Cu contains many edges and pores that induce an enhanced electric field, which could not only reduce the thermodynamic energy barrier for the formation of CO by forming concentrated K^+^ on the active site [182], but also confine the OH^−^ ions diffusion and thus promote the local pH, leading to enhanced selectivity of C_2+_ chemicals [183–185]. A flow cell was used to evaluate the performance of the mesoporous Cu nanoribbons for CO_2_RR in 1 M KOH electrolyte. The results show that such a catalyst can selectively produce C_2+_ product with FE up to 82.3% at the potential of −1.2 V vs RHE. At the same time, the partial current density toward C_2+_ product is up to 347.9 mA cm^−2^, which is sufficient to satisfy the requirements for industrial applications.

**Fig. 20:**
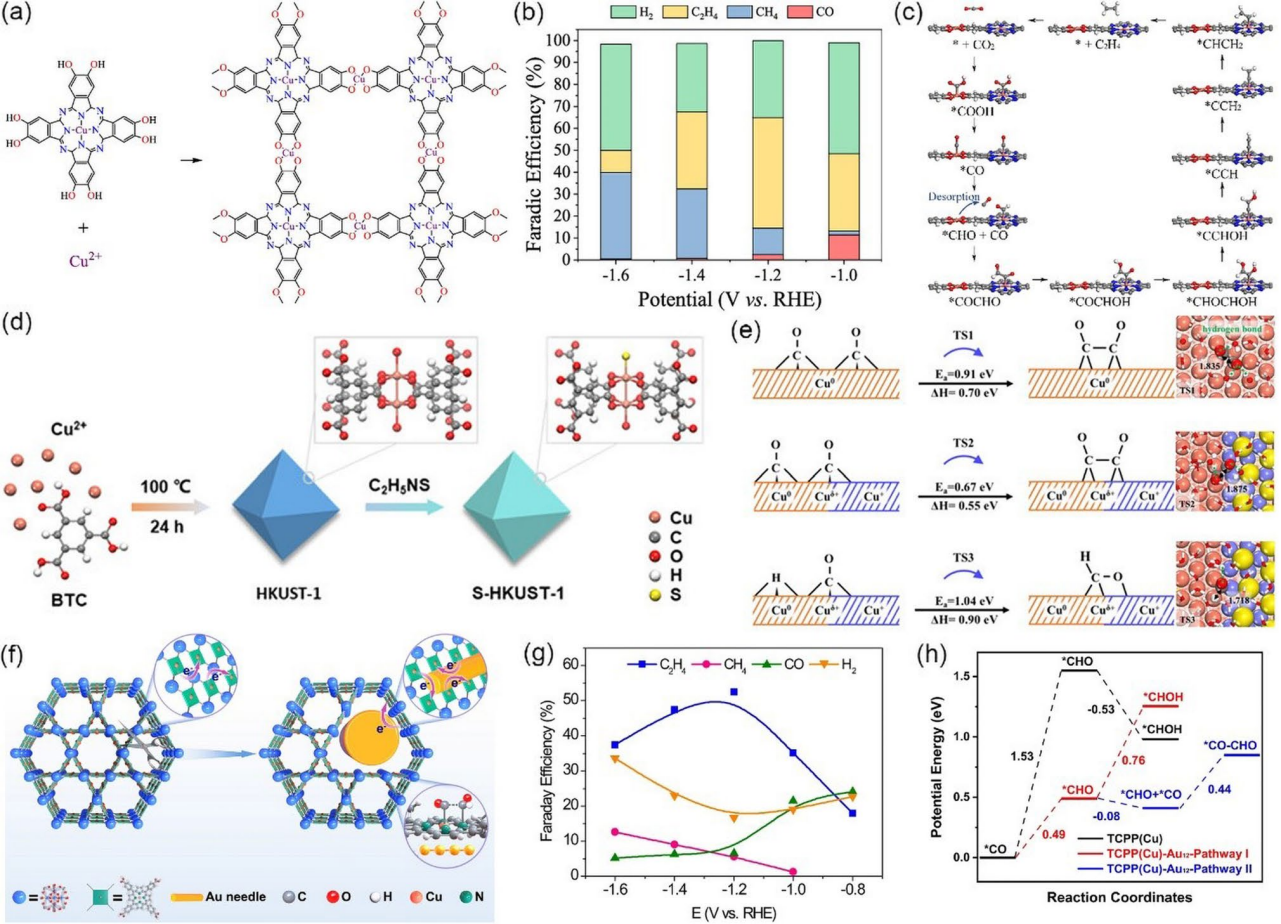
**a** Illustration of the structure of PcCu-Cu–O. **b** FEs of C_2_H_4_, CH_4_, CO, and H_2_ for PcCu-Cu–O. **c** Proposed CO_2_RR mechanism of PcCu-Cu–O. (Color codes: carbon (gray), nitrogen (blue), oxygen (red), hydrogen (white), copper (orange)). Reproduced with permission from Ref. [177]. **d** Schematic illustration of the preparation of S-HKUST-1, which indicates a local H_2_O molecule might be replaced by S atoms. **e** The reaction barriers together with enthalpies and corresponding transition state configurations for *CO dimerization and hydrogenation over Cu(111) and Cu/Cu_2_S surfaces, respectively. (Yellow, red, gray, white, orange and blue balls refer to S, O, C, H, Cu0 and Cu^δ+^ atoms, respectively). Reproduced with permission from Ref. [178]. **f** Impregnation of Au nanoneedles into PCN-222(Cu) with cleaved ligand-node linkage to alter the charge conduction path and steer the CO_2_RR pathway. **g** FEs of various reduction products for AuNN@PCN-222(Cu). **h** Formation energy of key intermediates along with the reaction coordinates for TCPP(Cu) and TCPP(Cu)-Au_12_. Reproduced with permission from Ref. [179]

It is universally acknowledged that the linker or ligand in MOFs plays an essential role in the selective reduction of CO_2_ to value-added chemicals. Choosing an appropriate linker not only determines the distance between metal centers but also regulates the electronic structure of MOFs, thereby influencing the activity and selectivity of CO_2_RR. For example, Qiu et al. [177] employed PcCu-(OH)_8_ (2,3,9,10,16,17,23,24-octahydroxy- phthalo-cyaninato)copper(II)) and CuCl_2_ to assemble a PcCu–Cu–O MOF (Fig. 20a). The as-obtained PcCu-Cu–O MOF was further exfoliated to small particles and coated on a glassy carbon electrode to prepare the working electrode. They used an H-type cell and CO_2_-saturated 0.1 M KHCO_3_ aqueous solution to evaluate the CO_2_RR performance of such material. As shown in Fig. 20b, such catalyst exhibits high selectivity toward C_2_H_4_ (FE = 50% at -1.2 V vs. RHE) and shows good stability after 4 h continuous electrolysis, reflecting the significant synergistic effect between CuPc and CuO_4_ nodes. Periodic DFT (PDFT) calculations show that the CuO_4_ unit serves as an active site for the formation of *CO, while the CuPc unit has a high adsorption energy for *CO and is beneficial for the hydrogenation of *CO toward *CHO. Further, the CO molecule desorbed on the CuO_4_ unit can easily dimerize with the absorbed *CHO intermediate on CuPc unit to form the *COCHO intermediate, and C_2_H_2_ is finally obtained through multi-step reactions (Fig. 20c). In situ ATR-FTIR analysis also confirmed the proposed C_2_H_4_ mechanism. As demonstrated by Wen et al. [178], sulfur doping in HKUST-1 can change the coordination environment of Cu nodes and generate a precatalyst (S-HKUST-1) with uniformly dispersed Cu–S motifs (Fig. 20d), and their in-situ reconstruction can yield abundant active biphasic Cu/Cu_x_S_y_ interfaces, which show good C_2_H_4_ selectivity and activity. Electrochemical experiments revealed that such a catalyst gets the highest C_2_H_4_ FE of 57.2% with a total current density of up to 400 mA cm^−2^. Compared with HKUST-1, S-HKUST-1 can derive more Cu^δ+^ species at the Cu/Cu_x_S_y_ interfaces after the reconstruction process. DFT calculations show that the S-stabilized Cu^δ+^ at the Cu/Cu_x_S_y_ interface has an optimized geometric and electronic structure for *CO dimerization (Fig. 20e), thus improving the CO_2_-to-C_2_H_4_ selectivity. In addition, Xie et al. [179] demonstrated that impregnating Au nanoneedles (AuNN) into a PCN-222(Cu) MOF (AuNN@PCN-222(Cu)) is also an effective way to improve the CO_2_-to-C_2_H_4_ performance (Fig. 20f). Electrochemical experiments revealed that the catalyst exhibits a maximal C_2_H_4_ FE of 52.5% at s−1.2 V vs. RHE (Fig. 20g). A series of experiments has been conducted to explore the reaction mechanisms and the results show that AuNN@PCN-222(Cu) serves as a tandem catalyst: CO generated by AuNN are further reduced on the metalloporphyrin sites, which is consistent with theoretical results (Fig. 20h). The AuNN plays a cable-like role in the PCN-222(Cu), which provides a charge transfer path to the metalloporphyrin center, thus enhancing the structural stability of AuNP@PCN-222(Cu).

**Fig. 21:**
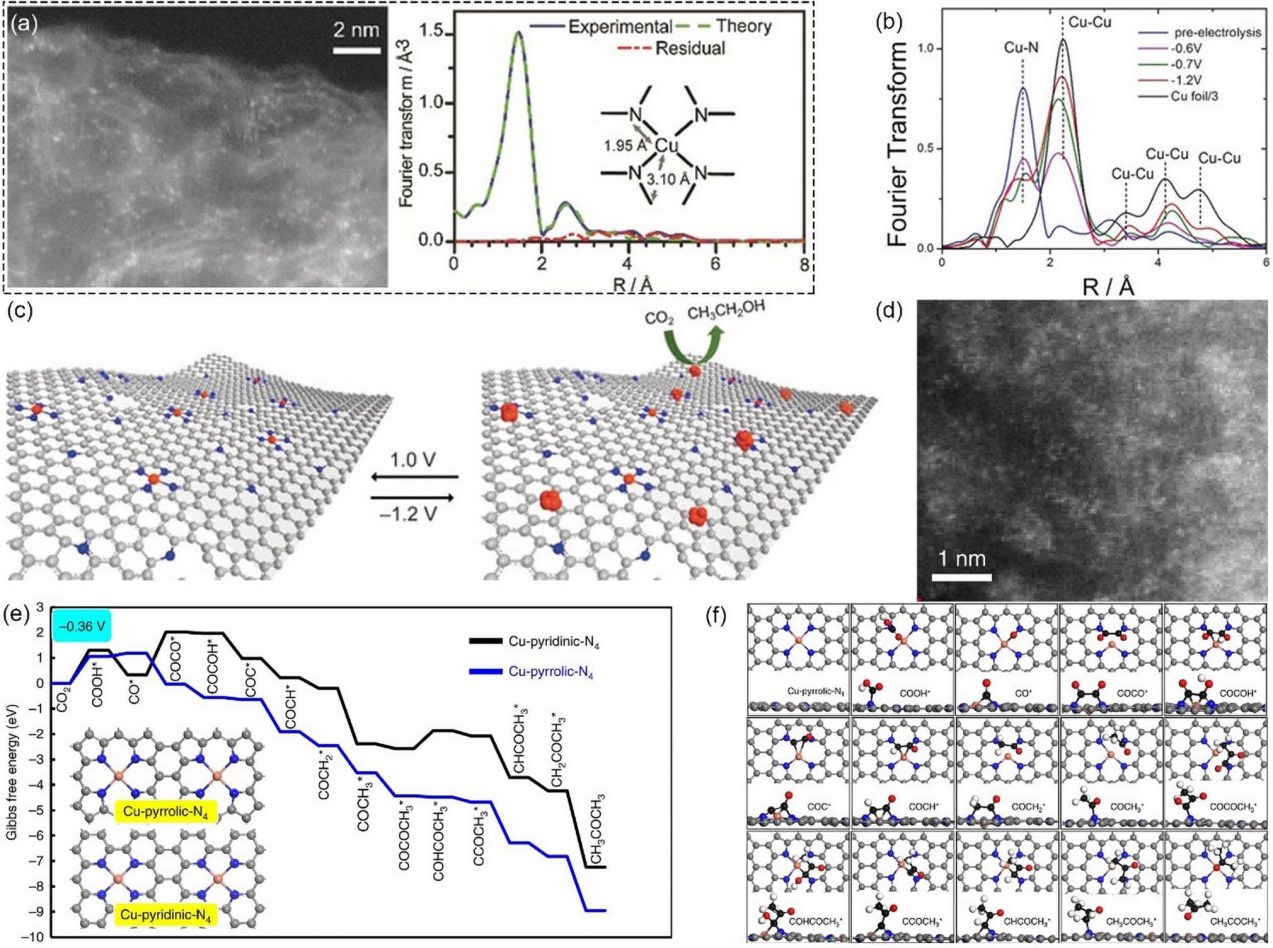
**a** Structural and morphological characterization of Cu_0.5_NC. **b** Fourier transform of the experimental EXAFS spectra of Cu_0.5_NC under operando electrolysis conditions. **c** Illustration of the reversible restructuration of metal sites. Reproduced with permission from Ref. [180]. **d** High-angle annular dark-field scanning transmission electron microscopy (HAADF-STEM) images of Cu-SA/NPC. **e** Free energy diagrams calculated at a potential of -0.36 V for CO_2_ reduction to CH_3_COCH_3_ on Cu-pyridinic-N_4_ and Cu-pyrrolic-N_4_ sites of Cu-SA/NPC. **f** Optimized structures of all reaction intermediates involved in the pathways of CO_2_ reduction on the Cu-pyrrolic-N_4_ site (gray: C of catalyst; black: C of adsorbate; red: O; orange: Cu; blue: N; white: H). Reproduced with permission from Ref. [181]

As mentioned above, SACs can effectively increase atomic utilization and enhance catalytic performance. Therefore, highly dispersed single-atom Cu catalysts with unique electronic structures may break the linear relationship between the intermediates and improve the selectivity of C_2+_ products by regulating the energy barrier of C–C dimerization. For example, by dispersing Cu atoms in a nitrogen-doped conductive carbon matrix, Karapinar et al. [180] synthesized a single-site Cu–N–C catalyst (Cu_0.5_NC) via a simple pyrolysis strategy from a dry-phase mixture of ZIF-8 and Cu^II^ precursor (Fig. 21a). The Cu site with four nitrogen coordinations (CuN_4_) enables selective CO_2_RR to CH_3_CH_2_OH with a maximum FE of 55% and exhibits a stable average current density of 16.2 mA cm^−2^ under the optimal conditions (potential of −1.2 V vs. RHE, 0.1 M CsHCO_3_ electrolyte, CO_2_ flow rate of 2.5 mL min^−1^ and gas-phase recycling set up). It is worth noting that the CuN_4_ coordination environment was destroyed during the electrochemical reaction and the oxidation state of Cu was shifted from + 2 to 0, resulting in Cu-Cu coordination, which was confirmed by XAS under operando electrolysis conditions (Fig. 21b). Therefore, the metallic Cu nanoparticles with an average size of 0.47 ± 0.04 nm were likely to be the real active species. Interestingly, the metallic Cu nanoparticles can be oxidized back to + 2 and the Cu–N_4_ site can also be restored when the used electrocatalyst is exposed to air or a positive potential is applied (Fig. 21c). This is likely the consequence of the small size of the particles and the strong Cu^II^-chelating capacity of the N_4_ sites of the material. This work rendered the assignment of single Cu as sole active sites questionable. In addition, Zhao et al. [181] synthesized a single-atom Cu on an N-doped porous carbon catalyst (Cu-SA/NPC, Fig. 21d) by the carbonization of Cu-doped ZIF-8 precursor, and Cu-SA/NPC exhibited considerable CO_2_–to–CH_3_COCH_3_ activity at a low overpotential. EXAFS fitting showed that the Cu species in Cu-SA/NPC are atomically dispersed and coordinated with four pyrrole N atoms, resulting in Cu-pyrrolic-N_4_ active sites, which facilitates the C–C coupling and stabilizes the reaction intermediates for acetone production. The FE of CH_3_COCH_3_ reached a maximum value of 36.7% at the potential of −0.36 V vs. RHE in a CO_2_− saturated 0.1 M KHCO_3_ solution. DFT calculations revealed that the conversion of CO_2_ to CH_3_COCH_3_ was thermodynamically favorable on the Cu-pyrrolic-N_4_ sites of Cu-SA/NPC and the formation of *COOH is the RDS for CH_3_COCH_3_ production (Fig. 21e). Moreover, the synergy effect of Cu and coordinated pyrrolic N species optimize the adsorption configuration of reaction intermediates on the catalyst surface, and thus promoting the reaction toward the CH_3_COCH_3_ product (Fig. 21f). Notably, they also demonstrated that both uncoordinated pyrrolic-N_4_ and oxidized N in Cu-SA/NPC do not contribute to acetone formation.

## Summary and Outlook

Transforming waste CO_2_ into value-added fuels and chemicals by using renewable energy is an environmentally friendly way to reduce CO_2_ emissions and help build a sustainable future. But implementing large-scale CO_2_RR technologies still has a long way to go to achieve social and economic benefits. One of the most challenging parts is the design of efficient catalysts that can break the scaling relations and show high selectivity and activity toward a specific reaction pathway. In this review, we first took a detailed look at the CO_2_RR mechanisms for different products and provide crucial insights for designing efficient catalysts, with a special focus on the design strategies for efficient and selective MOF-related catalysts such as pristine MOFs and MOFs-derived materials (single-atom catalysts, clusters, metallic NPs or hybrid). Then we discussed the current applications of these catalysts in the CO_2_RR. Although tremendous progress has been achieved, there are still many challenges that need to be addressed before MOF-related catalysts can be applied in industrial applications.

First of all, the real active sites for the CO_2_RR need to be clarified since the coordination between metal nodes and organic linkers plays a significant role in the formation of MOFs, which greatly affect the structural robustness of MOFs especially when negative potentials are applied. There are considerable MOFs that have been identified as being stable when they are used as electrocatalysts, but the stability of the electrocatalyst is only verified by the crystal structure of the MOFs in the CO_2_RR reaction media and/or some post-electrocatalysis analysis (XRD, SEM, XPS, and TEM). To obtain a better understanding of the MOFs’ role in the targeted reactions (real catalysts or precatalysts), it is vital to combine different techniques (especially advanced in situ/operando characterization techniques) to monitor the possible reconstruction of MOFs that may occur during reductive electrolysis. A deep insight into the structure of MOFs during the CO_2_RR process would also help to uncover the catalytic mechanism, and thus further guide the rational design of more efficient electrocatalysts.

Moreover, in order to avoid the stability problems, it is also expected that more MOFs with high stability toward water/moisture, acid, and base can be synthesized by incorporating the second type of metal center that is less affected by hydrolysis. The introduction of functional groups such as carboxyl, amino, or phosphonate groups could also improve the stability of MOFs by providing anchoring sites for the metal nodes and enhancing the interaction between the metal nodes and the organic linkers. Furthermore, MOFs are good platforms for modulating the electronic structure of the active sites. We can reasonably adjust the local environment of the active site by molecular or surface engineering, such as the reasonable design of organic ligands and incorporation of coordination heteroatoms, etc. For example, unsaturated sites can be formed by removing part of the linkers. The interaction between under-coordinated sites and reactants or reaction intermediates can tune the local concentration of reactants or intermediates around the active site, thus facilitating the intrinsic activity of CO_2_RR. Functional groups on the MOF materials can also play an important role in regulating the selectivity of CO_2_RR toward specific value-added products. It could provide additional sites for CO_2_ adsorption and facilitate the activation of CO_2_. For instance, the existence of N-containing functional groups would activate the protons to acquire COH* or CHO* intermediate [153–156], which are crucial intermediates for further hydrogenation to produce CH_4_. Thus, incorporating amino or nitryl groups onto MOF-related materials may represent a viable strategy for improving the selectivity of carbon dioxide reduction to methane. In addition, as mentioned above, most MOFs are synthesized by harsh solvothermal methods using costly ligands and organic solvents, making the technology unaffordable both economically and environmentally. Therefore, a cost-effective and environment-friendly commercial synthetic approach needs to be explored in future research.

The low conductivity of MOFs is another substantial drawback that greatly hinders the electron transfer in the CO_2_RR, resulting in low electrocatalytic activity. Combining MOFs with conductive materials (carbon black, carbon nanotube), introducing guest redox molecules into the frameworks, or choosing the electron-donating ligands are effective strategies to design more conductive MOFs. MOFs with high electrical conductivity can facilitate the transfer of electrons from the electrode to the catalytic sites, leading to enhanced electrocatalytic activity and selectivity toward specific products. Electrical or thermal treatment is also a promising way to convert MOFs into conductive and stable MOF-derived materials (such as metal oxides, alloy, metal on carbon supports, carbon-based SACs, metal-free materials, and nanocomposites). The thermal treatment under an inert atmosphere could retain the porous structure of the original MOF and uniformly dispersed metal sites. Moreover, it allows for the precise control of the material properties, such as the morphology, composition, surface area, and the electronic structure of the metal nodes, which can significantly improve the CO_2_RR performance. To enhance atomic utilization efficiency, strategies are needed to anchor metal atoms on the available surface of catalysts to synthesize SACs with a high density of single-atom sites, while avoiding the formation of any clusters or particles encapsulated within the carbon matrix.

As we have discussed above, most studies are devoted to improving the selectivity toward target products, while strategies to improve the efficiency of CO_2_ utilization are rarely mentioned. The CO_2_ utilization efficiency should be further highlighted in future studies. It seems that the CO_2_RR under acidic conditions could be a feasible way for industrial  applications. Moreover, in traditional H-cell or flow-cell reactors, the generated products, either gas or liquid, were mixed with the electrolytes (KHCO_3_, KCl or KOH) or H_2_ and surplus CO_2_. An immediate outlook for large-scale industrial applications points toward the use of systems that directly obtain pure products, avoiding extra separation and concentration processes. For gas products (such as CO, CH_4_, C_2_H_4_) we can combine the advantages of MOF materials, including catalysis and gas separation, to synthesize a functionalized catalyst. Such catalysts can not only effectively convert CO_2_ into gas products, but also be used as adsorption and separation materials to purify the gas products, finally obtaining pure gas hydrocarbons. For liquid products, several studies have demonstrated that a modified solid-state electrolyte MEA electrolyzer can generate pure liquid products such as HCOOH and CH_3_COOH [9, 186, 187]. However, other pure liquid products, such as alcohols, are rarely reported. It is worth noting that some organic products could damage the solid-state electrolyte and ion-exchange membranes during long-term operation, leading to poor durability. Therefore, optimizing the solid-state electrolyte electrolyzer system for different products is necessary.

Overall, it is not hard to predict a promising foreground of MOF-related materials as CO_2_RR electrocatalysts. Although there are still many challenges in optimizing electrolyzer configurations and MOF-related catalysts for achieving higher activity, selectivity, and stability, it is believed that with continuous research efforts and development, MOF-related catalysts will find their unique positions in building a closed-loop anthropogenic carbon cycle in the near future.

## References

[1] Chu S, Majumdar A (2012). Opportunities and challenges for a sustainable energy future. Nature.

[2] Obama B (2017). The irreversible momentum of clean energy. Science.

[3] Chu S, Cui Y, Liu N (2016). The path towards sustainable energy. Nat. Mater..

[4] Mexis I, Todeschini G (2020). Battery energy storage systems in the United Kingdom: a review of current state-of-the-art and future applications. Energies.

[5] Luna PD, Hahn C, Higgins D, Jaffer SA, Jaramillo TF (2019). What would it take for renewably powered electrosynthesis to displace petrochemical processes?. Science.

[6] Greenblatt JB, Miller DJ, Ager JW, Houle FA, Sharp ID (2018). The technical and energetic challenges of separating (photo)electrochemical carbon dioxide reduction products. Joule.

[7] Bushuyev OS, Luna PD, Dinh CT, Tao L, Saur G (2018). What should we make with CO_2_ and how can we make it?. Joule.

[8] Zheng T, Liu C, Guo C, Zhang M, Li X (2021). Copper-catalysed exclusive CO_2_ to pure formic acid conversion via single-atom alloying. Nat. Nanotechnol..

[9] Xia C, Zhu P, Jiang Q, Pan Y, Liang W (2019). Continuous production of pure liquid fuel solutions via electrocatalytic CO_2_ reduction using solid-electrolyte devices. Nat. Energy.

[10] Wang X, Wang Z, García de Arquer FP, Dinh C-T, Ozden A (2020). Efficient electrically powered CO_2_-to-ethanol via suppression of deoxygenation. Nat. Energy.

[11] Birdja YY, Pérez-Gallent E, Figueiredo MC, Göttle AJ, Calle-Vallejo F (2019). Advances and challenges in understanding the electrocatalytic conversion of carbon dioxide to fuels. Nat. Energy.

[12] Li J, Zeng H, Dong X, Ding Y, Hu S (2023). Selective CO_2_ electrolysis to co using isolated antimony alloyed copper. Nat. Commun..

[13] Fan L, Xia C, Yang FQ, Wang J, Wang HT (2020). Strategies in catalysts and electrolyzer design for electrochemical CO_2_ reduction toward C_2+_ products. Sci. Adv..

[14] Li Y, Shi G, Chen T, Zhu L, Yu D (2022). Simultaneous increase of conductivity, active sites and structural strain by nitrogen injection for high-yield CO_2_ electro-hydrogenation to liquid fuel. Appl. Catal. B Environ..

[15] Yu R, Qiu C, Lin Z, Liu H, Gao J (2022). CeO_x_ promoted electrocatalytic CO_2_ reduction to formate by assisting in the critical hydrogenation step. ACS Mater. Lett..

[16] Li J, Abbas SU, Wang H, Zhang Z, Hu W (2021). Recent advances in interface engineering for electrocatalytic CO_2_ reduction reaction. Nano-Micro Lett..

[17] Xue D, Xia H, Yan W, Zhang J, Mu S (2020). Defect engineering on carbon-based catalysts for electrocatalytic CO_2_ reduction. Nano-Micro Lett..

[18] Qiao J, Liu Y, Hong F, Zhang J (2014). A review of catalysts for the electroreduction of carbon dioxide to produce low-carbon fuels. Chem. Soc. Rev..

[19] Liu C, Zhang M, Li J, Xue W, Zheng T (2022). Nanoconfinement engineering over hollow multi-shell structured copper towards efficient electrocatalytical C-C coupling. Angew. Chem. Int. Ed..

[20] Tang T, Wang Z, Guan J (2022). Optimizing the electrocatalytic selectivity of carbon dioxide reduction reaction by regulating the electronic structure of single-atom m-n-c materials. Adv. Funct. Mater..

[21] Zhu J, Wang Y, Zhi A, Chen Z, Shi L (2022). Cation-deficiency-dependent CO_2_ electroreduction over copper-based ruddlesden-popper perovskite oxides. Angew. Chem. Int. Ed..

[22] Ahmad T, Liu S, Sajid M, Li K, Ali M (2022). Electrochemical CO_2_ reduction to C_2+_ products using Cu-based electrocatalysts: a review. Nano Res. Energy.

[23] Chen J, Wang L (2022). Effects of the catalyst dynamic changes and influence of the reaction environment on the performance of electrochemical CO_2_ reduction. Adv. Mater..

[24] Zhang B, Zhang B, Jiang Y, Ma T, Pan H (2021). Single-atom electrocatalysts for multi-electron reduction of CO_2_. Small.

[25] Abednatanzi S, Derakhshandeh PG, Depauw H, Coudert FX, Vrielinck H (2019). Mixed-metal metal-organic frameworks. Chem. Soc. Rev..

[26] Huang G, Yang L, Yin Q, Fang ZB, Hu XJ (2020). A comparison of two isoreticular metal-organic frameworks with cationic and neutral skeletons: stability, mechanism, and catalytic activity. Angew. Chem. Int. Ed..

[27] Furukawa H, Cordova KE, O'Keeffe M, Yaghi OM (2013). The chemistry and applications of metal-organic frameworks. Science.

[28] Khan U, Nairan A, Gao J, Zhang Q (2022). Current progress in 2D metal-organic frameworks for electrocatalysis. Small Struct.

[29] Wu Y, Li Y, Gao J, Zhang Q (2021). Recent advances in vacancy engineering of metal-organic frameworks and their derivatives for electrocatalysis. SusMat..

[30] Dong R, Han P, Arora H, Ballabio M, Karakusn M (2018). High-mobility band-like charge transport in a semiconducting two-dimensional metal-organic framework. Nat. Mater..

[31] Gao J, Qian X, Lin RB, Krishna R, Wu H (2020). Mixed metal-organic framework with multiple binding sites for efficient C_2_H_2_/CO_2_ separation. Angew. Chem. Int. Ed..

[32] Cui Y, Zhang J, He H, Qian G (2018). Photonic functional metal-organic frameworks. Chem. Soc. Rev..

[33] Wu HB, Lou XW (2017). Metal-organic frameworks and their derived materials for electrochemical energy storage and conversion: promises and challenges. Sci. Adv..

[34] Liang J, Liang Z, Zou R, Zhao Y (2017). Heterogeneous catalysis in zeolites, mesoporous silica, and metal-organic frameworks. Adv. Mater..

[35] Du M, Li Q, Zhao Y, Liu C-S, Pang H (2020). A review of electrochemical energy storage behaviors based on pristine metal-organic frameworks and their composites. Coordin. Chem. Rev..

[36] Shah SSA, Najam T, Aslam MK, Ashfaq M, Rahman MM (2020). Recent advances on oxygen reduction electrocatalysis: correlating the characteristic properties of metal organic frameworks and the derived nanomaterials. Appl. Catal. B Environ..

[37] Zhang H, Nai J, Yu L, Lou XW (2017). Metal-organic-framework-based materials as platforms for renewable energy and environmental applications. Joule.

[38] Yi F-Y, Zhang R, Wang H, Chen L-F, Han L (2017). Metal-organic frameworks and their composites: synthesis and electrochemical applications. Small Methods.

[39] Guan BY, Yu XY, Wu HB, Lou XWD (2017). Complex nanostructures from materials based on metal-organic frameworks for electrochemical energy storage and conversion. Adv. Mater..

[40] Maina JW, Pozo-Gonzalo C, Schütz JA, Wang J, Dumée LF (2019). Tuning CO_2_ conversion product selectivity of metal organic frameworks derived hybrid carbon photoelectrocatalytic reactors. Carbon.

[41] Sun J-K, Xu Q (2014). Functional materials derived from open framework templates/precursors: synthesis and applications. Energy Environ. Sci..

[42] Cao X, Tan C, Sindoro M, Zhang H (2017). Hybrid micro-/nano-structures derived from metal-organic frameworks: preparation and applications in energy storage and conversion. Chem. Soc. Rev..

[43] Liu J, Zhu D, Guo C, Vasileff A, Qiao SZ (2017). Design strategies toward advanced mof-derived electrocatalysts for energy-conversion reactions. Adv. Energy Mater..

[44] Lu XF, Fang Y, Luan D, Lou XWD (2021). Metal-organic frameworks derived functional materials for electrochemical energy storage and conversion: a mini review. Nano Lett..

[45] Yan R, Ma T, Cheng M, Tao X, Yang Z (2021). Metal-organic-framework-derived nanostructures as multifaceted electrodes in metal-sulfur batteries. Adv. Mater..

[46] Sanati S, Abazari R, Albero J, Morsali A, Garcia H (2021). Metal-organic framework derived bimetallic materials for electrochemical energy storage. Angew. Chem. Int. Ed..

[47] Liang Z, Qiu T, Gao S, Zhong R, Zou R (2021). Multi-scale design of metal–organic framework-derived materials for energy electrocatalysis. Adv. Energy Mater..

[48] Li L, Li XD, Sun YF, Xie Y (2022). Rational design of electrocatalytic carbon dioxide reduction for a zero-carbon network. Chem. Soc. Rev..

[49] Kortlever R, Shen J, Schouten KJ, Calle-Vallejo F, Koper MT (2015). Catalysts and reaction pathways for the electrochemical reduction of carbon dioxide. J. Phys. Chem. Lett..

[50] Koper MTM (2011). Thermodynamic theory of multi-electron transfer reactions: implications for electrocatalysis. J. Electroanal. Chem..

[51] Handoko AD, Wei F, Jenndy BS, Yeo Z.W. She (2018). Understanding heterogeneous electrocatalytic carbon dioxide reduction through operando techniques. Nat. Catal..

[52] Nitopi S, Bertheussen E, Scott SB, Liu X, Engstfeld AK (2019). Progress and perspectives of electrochemical CO_2_ reduction on copper in aqueous electrolyte. Chem. Rev..

[53] Benson EE, Kubiak CP, Sathrum AJ, Smieja JM (2009). Electrocatalytic and homogeneous approaches to conversion of CO_2_ to liquid fuels. Chem. Soc. Rev..

[54] Wu J, Huang Y, Ye W, Li Y (2017). CO_2_ reduction: from the electrochemical to photochemical approach. Adv. Sci..

[55] Feaster JT, Shi C, Cave ER, Hatsukade T, Abram DN (2017). Understanding selectivity for the electrochemical reduction of carbon dioxide to formic acid and carbon monoxide on metal electrodes. ACS Catal..

[56] Zhang S, Kang P, Meyer TJ (2014). Nanostructured tin catalysts for selective electrochemical reduction of carbon dioxide to formate. J. Am. Chem. Soc..

[57] Sun Z, Ma T, Tao H, Fan Q, Han B (2017). Fundamentals and challenges of electrochemical CO_2_ reduction using two-dimensional materials. Chem.

[58] Schmid B, Reller C, Neubauer S, Fleischer M, Dorta R (2017). Reactivity of copper electrodes towards functional groups and small molecules in the context of CO_2_ electro-reductions. Catalysts.

[59] Peterson AA, Abild-Pedersen F, Studt F, Rossmeisl J, Norskov JK (2010). How copper catalyzes the electroreduction of carbon dioxide into hydrocarbon fuels. Energy Environ. Sci..

[60] Li S, Duan H, Yu J, Qiu C, Yu R (2022). Cu vacancy induced product switching from formate to Co for CO_2_ reduction on copper sulfide. ACS Catal..

[61] Garza AJ, Bell AT, Head-Gordon M (2018). Mechanism of CO_2_ reduction at copper surfaces: pathways to C_2_ products. ACS Catal..

[62] Schouten KJ, Qin Z, Gallent EP, Koper MT (2012). Two pathways for the formation of ethylene in co reduction on single-crystal copper electrodes. J. Am. Chem. Soc..

[63] Nie X, Esopi MR, Janik MJ, Asthagiri A (2013). Selectivity of CO_2_ reduction on copper electrodes: the role of the kinetics of elementary steps. Angew. Chem. Int. Ed..

[64] Hori Y, Takahashi R, Yoshinami Y, Murata A (1997). Electrochemical reduction of CO at a copper electrode. J. Phys. Chem. B.

[65] Ma S, Sadakiyo M, Luo R, Heima M, Yamauchi M (2016). One-step electrosynthesis of ethylene and ethanol from CO_2_ in an alkaline electrolyzer. J. Power Sources.

[66] Calle-Vallejo F, Koper MT (2013). Theoretical considerations on the electroreduction of CO to C_2_ species on Cu(100) electrodes. Angew. Chem. Int. Ed..

[67] Schouten KJP, Kwon Y, van der Ham CJM, Qin Z, Koper MTM (2011). A new mechanism for the selectivity to C_1_ and C_2_ species in the electrochemical reduction of carbon dioxide on copper electrodes. Chem. Sci..

[68] Montoya JH, Shi C, Chan K, Norskov JK (2015). Theoretical insights into a co dimerization mechanism in CO_2_ electroreduction. J. Phys. Chem. Lett..

[69] Goodpaster JD, Bell AT, Head-Gordon M (2016). Identification of possible pathways for c-c bond formation during electrochemical reduction of CO_2_: new theoretical insights from an improved electrochemical model. J. Phys. Chem. Lett..

[70] Burdyny T, Smith WA (2019). CO_2_ reduction on gas-diffusion electrodes and why catalytic performance must be assessed at commercially-relevant conditions. Energy Environ. Sci..

[71] Singh MR, Clark EL, Bell AT (2015). Effects of electrolyte, catalyst, and membrane composition and operating conditions on the performance of solar-driven electrochemical reduction of carbon dioxide. Phys. Chem. Chem. Phys..

[72] Wang Y, Han P, Lv X, Zhang L, Zheng G (2018). Defect and interface engineering for aqueous electrocatalytic CO_2_ reduction. Joule.

[73] Yang Y, Li F (2021). Reactor design for electrochemical CO_2_ conversion toward large-scale applications. Curr. Opin. Green Sust..

[74] Weng LC, Bell AT, Weber AZ (2018). Modeling gas-diffusion electrodes for CO_2_ reduction. Phys. Chem. Chem. Phys..

[75] Chen C, Kotyk JFK, Sheehan SW (2018). Progress toward commercial application of electrochemical carbon dioxide reduction. Chem.

[76] Wakerley D, Lamaison S, Wicks J, Clemens A, Feaster J (2022). Gas diffusion electrodes, reactor designs and key metrics of low-temperature CO_2_ electrolysers. Nat. Energy.

[77] Xing Z, Hu L, Ripatti DS, Hu X, Feng X (2021). Enhancing carbon dioxide gas-diffusion electrolysis by creating a hydrophobic catalyst microenvironment. Nat. Commun..

[78] de Arquer FPG, Dinh CT, Ozden A, Wicks J, McCallum C (2020). CO_2_ electrolysis to multicarbon products at activities greater than 1 A cm^-2^. Science.

[79] Chen R, Su HY, Liu D, Huang R, Meng X (2020). Highly selective production of ethylene by the electroreduction of carbon monoxide. Angew. Chem. Int. Ed..

[80] Verma S, Hamasaki Y, Kim C, Huang W, Lu S (2017). Insights into the low overpotential electroreduction of CO_2_ to co on a supported gold catalyst in an alkaline flow electrolyzer. ACS Energy Lett..

[81] Zhuang T-T, Liang Z-Q, Seifitokaldani A, Li Y, Luna PD (2018). Steering post-c–c coupling selectivity enables high efficiency electroreduction of carbon dioxide to multi-carbon alcohols. Nat. Catal..

[82] Bhargava SS, Proietto F, Azmoodeh D, Cofell ER, Henckel DA (2020). System design rules for intensifying the electrochemical reduction of CO_2_ to Co on Ag nanoparticles. ChemElectroChem.

[83] Gao F-Y, Bao R-C, Gao M-R, Yu S-H (2020). Electrochemical CO_2_-to-CO conversion: electrocatalysts, electrolytes, and electrolyzers. J. Mater. Chem. A.

[84] Nesbitt NT, Burdyny T, Simonson H, Salvatore D, Bohra D (2020). Liquid–solid boundaries dominate activity of CO_2_ reduction on gas-diffusion electrodes. ACS Catal..

[85] Yin Z, Peng H, Wei X, Zhou H, Gong J (2019). An alkaline polymer electrolyte CO_2_ electrolyzer operated with pure water. Energy Environ. Sci..

[86] Endrődi B, Kecsenovity E, Samu A, Halmágyi T, Rojas-Carbonell S (2020). High carbonate ion conductance of a robust piperion membrane allows industrial current density and conversion in a zero-gap carbon dioxide electrolyzer cell. Energy Environ. Sci..

[87] Gabardo CM, O’Brien CP, Edwards JP, McCallum C, Xu Y (2019). Continuous carbon dioxide electroreduction to concentrated multi-carbon products using a membrane electrode assembly. Joule.

[88] Ge L, Rabiee H, Li M, Subramanian S, Zheng Y (2022). Electrochemical CO_2_ reduction in membrane-electrode assemblies. Chem.

[89] Gawel A, Jaster T, Siegmund D, Holzmann J, Lohmann H (2022). Electrochemical CO_2_ reduction—the macroscopic world of electrode design, reactor concepts & economic aspects. iScience.

[90] Weng L-C, Bell AT, Weber AZ (2019). Towards membrane-electrode assembly systems for CO_2_ reduction: A modeling study. Energy Environ. Sci..

[91] Hinogami R, Yotsuhashi S, Deguchi M, Zenitani Y, Hashiba H (2012). Electrochemical reduction of carbon dioxide using a copper rubeanate metal organic framework. ECS Electrochem. Lett..

[92] Kumar RS, Kumar SS, Kulandainathan MA (2012). Highly selective electrochemical reduction of carbon dioxide using Cu based metal organic framework as an electrocatalyst. Electrochem. Commun..

[93] Monteiro MCO, Philips MF, Schouten KJP, Koper MTM (2021). Efficiency and selectivity of CO_2_ reduction to co on gold gas diffusion electrodes in acidic media. Nat. Commun..

[94] Verma S, Kim B, Jhong HR, Ma S, Kenis PJ (2016). A gross-margin model for defining technoeconomic benchmarks in the electroreduction of CO_2_. ChemSusChem.

[95] Jouny M, Luc W, Jiao F (2018). General techno-economic analysis of CO_2_ electrolysis systems. Ind. Eng. Chem. Res..

[96] Kornienko N, Zhao Y, Kley CS, Zhu C, Kim D (2015). Metal-organic frameworks for electrocatalytic reduction of carbon dioxide. J. Am. Chem. Soc..

[97] Dong B-X, Qian S-L, Bu F-Y, Wu Y-C, Feng L-G (2018). Electrochemical reduction of CO_2_ to co by a heterogeneous catalyst of fe–porphyrin-based metal–organic framework. ACS Appl. Energy Mater..

[98] Zhang M-D, Si D-H, Yi J-D, Yin Q, Huang Y-B (2021). Conductive phthalocyanine-based metal-organic framework as a highly efficient electrocatalyst for carbon dioxide reduction reaction. Sci. China Chem..

[99] Yi JD, Si DH, Xie R, Yin Q, Zhang MD (2021). Conductive two-dimensional phthalocyanine-based metal-organic framework nanosheets for efficient electroreduction of CO_2_. Angew. Chem. Int. Ed..

[100] Majidi L, Ahmadiparidari A, Shan N, Misal SN, Kumar K (2021). 2D copper tetrahydroxyquinone conductive metal-organic framework for selective CO_2_ electrocatalysis at low overpotentials. Adv. Mater..

[101] Zhong H, Ghorbani-Asl M, Ly KH, Zhang J, Ge J (2020). Synergistic electroreduction of carbon dioxide to carbon monoxide on bimetallic layered conjugated metal-organic frameworks. Nat. Commun..

[102] Dou S, Song J, Xi S, Du Y, Wang J (2019). Boosting electrochemical CO_2_ reduction on metal-organic frameworks via ligand doping. Angew. Chem. Int. Ed..

[103] Huang Q, Li Q, Liu J, Wang YR, Wang R (2019). Disclosing CO_2_ activation mechanism by hydroxyl-induced crystalline structure transformation in electrocatalytic process. Matter.

[104] Al-Attas TA, Marei NN, Yong X, Yasri NG, Thangadurai V (2021). Ligand-engineered metal-organic frameworks for electrochemical reduction of carbon dioxide to carbon monoxide. ACS Catal..

[105] Xin Z, Wang Y-R, Chen Y, Li W-L, Dong L-Z (2020). Metallocene implanted metalloporphyrin organic framework for highly selective CO_2_ electroreduction. Nano Energy.

[106] Xin Z, Liu J, Wang X, Shen K, Yuan Z (2021). Implanting polypyrrole in metal-porphyrin MOFs: enhanced electrocatalytic performance for CO_2_RR. ACS Appl. Mater. Interfaces.

[107] Gong YN, Jiao L, Qian YY, Pan CY, Zheng LR (2020). Regulating the coordination environment of mof-templated single-atom nickel electrocatalysts for boosting CO_2_ reduction. Angew. Chem. Int. Ed..

[108] Chen Z, Zhang X, Liu W, Jiao M, Mou K (2021). Amination strategy to boost the CO_2_ electroreduction current density of M-N/C single-atom catalysts to the industrial application level. Energy Environ. Sci..

[109] Lin L, Li H, Yan C, Li H, Si R (2019). Synergistic catalysis over iron-nitrogen sites anchored with cobalt phthalocyanine for efficient CO_2_ electroreduction. Adv. Mater..

[110] Han J, An P, Liu S, Zhang X, Wang D (2019). Reordering d orbital energies of single-site catalysts for CO_2_ electroreduction. Angew. Chem. Int. Ed..

[111] Ye L, Chen X, Gao Y, Ding X, Hou J (2021). Ultrathin two-dimensional metal–organic framework nanosheets for efficient electrochemical CO_2_ reduction. J. Energy Chem..

[112] Yan T, Wang P, Xu ZH, Sun WY (2022). Copper(ii) frameworks with varied active site distribution for modulating selectivity of carbon dioxide electroreduction. ACS Appl. Mater. Interfaces.

[113] Guo Y, Shi W, Yang H, He Q, Zeng Z (2019). Cooperative stabilization of the [pyridinium-CO_2_-CO] adduct on a metal-organic layer enhances electrocatalytic CO_2_ reduction. J. Am. Chem. Soc..

[114] Lu Y, Zhong H, Li J, Dominic AM, Hu Y (2022). Sp-carbon incorporated conductive metal-organic framework as photocathode for photoelectrochemical hydrogen generation. Angew. Chem. Int. Ed..

[115] Zhong H, Ly KH, Wang M, Krupskaya Y, Han X (2019). A phthalocyanine-based layered two-dimensional conjugated metal-organic framework as a highly efficient electrocatalyst for the oxygen reduction reaction. Angew. Chem. Int. Ed..

[116] Wang M, Dong R, Feng X (2021). Two-dimensional conjugated metal-organic frameworks (2d C-MOFs): Chemistry and function for moftronics. Chem. Soc. Rev..

[117] Guo Z, Chen G, Cometto C, Ma B, Zhao H (2019). Selectivity control of co versus HCOO^−^ production in the visible-light-driven catalytic reduction of CO_2_ with two cooperative metal sites. Nat. Catal..

[118] Mellmann D, Sponholz P, Junge H, Beller M (2016). Formic acid as a hydrogen storage material-development of homogeneous catalysts for selective hydrogen release. Chem. Soc. Rev..

[119] Calzadiaz-Ramirez L, Meyer AS (2022). Formate dehydrogenases for CO_2_ utilization. Curr. Opin. Biotech..

[120] Zhou Y, Liu S, Gu Y, Wen GH, Ma J (2021). In(iii) metal-organic framework incorporated with enzyme-mimicking nickel bis(dithiolene) ligand for highly selective CO_2_ electroreduction. J. Am. Chem. Soc..

[121] Zhu ZH, Zhao BH, Hou SL, Jiang XL, Liang ZL (2021). A facile strategy for constructing a carbon-particle-modified metal-organic framework for enhancing the efficiency of CO_2_ electroreduction into formate. Angew. Chem. Int. Ed..

[122] Qiu C, Qian K, Yu J, Sun M, Cao S (2022). Mof-transformed In_2_O_3-x_@C nanocorn electrocatalyst for efficient CO_2_ reduction to HCOOH. Nano-Micro Lett..

[123] Geng W, Chen W, Li G, Dong X, Song Y (2020). Induced CO_2_ electroreduction to formic acid on metal-organic frameworks via node doping. ChemSusChem.

[124] Deng Y, Wang S, Huang Y, Li X (2022). Structural reconstruction of Sn-based metal-organic frameworks for efficient electrochemical CO_2_ reduction to formate. Chin. J. Chem. Eng..

[125] Wu JX, Zhu XR, Liang T, Zhang XD, Hou SZ (2021). Sn(101) derived from metal-organic frameworks for efficient electrocatalytic reduction of CO_2_. Inorg. Chem..

[126] Zhang X, Zhang Y, Li Q, Zhou X, Li Q (2020). Highly efficient and durable aqueous electrocatalytic reduction of CO_2_ to HCOOH with a novel bismuth-MOF: experimental and dft studies. J. Mater. Chem. A.

[127] Li F, Gu GH, Choi C, Kolla P, Hong S (2020). Highly stable two-dimensional bismuth metal-organic frameworks for efficient electrochemical reduction of CO_2_. Appl. Catal. B Environ..

[128] Yang J, Wang X, Qu Y, Wang X, Huo H (2020). Bi-based metal-organic framework derived leafy bismuth nanosheets for carbon dioxide electroreduction. Adv. Energy Mater..

[129] Deng P, Yang F, Wang Z, Chen S, Zhou Y (2020). Metal-organic framework-derived carbon nanorods encapsulating bismuth oxides for rapid and selective CO_2_ electroreduction to formate. Angew. Chem. Int. Ed..

[130] Ying Y, Khezri B, Kosina J, Pumera M (2021). Reconstructed bismuth-based metal-organic framework nanofibers for selective CO_2_-to-formate conversion: Morphology engineering. ChemSusChem.

[131] Wang Q, Yang X, Zang H, Chen F, Wang C (2022). Metal-organic framework-derived biin bimetallic oxide nanoparticles embedded in carbon networks for efficient electrochemical reduction of CO_2_ to formate. Inorg. Chem..

[132] Cao C, Ma DD, Gu JF, Xie X, Zeng G (2020). Metal-organic layers leading to atomically thin bismuthene for efficient carbon dioxide electroreduction to liquid fuel. Angew. Chem. Int. Ed..

[133] Yuan W-W, Wu J-X, Zhang X-D, Hou S-Z, Xu M (2020). In situ transformation of bismuth metal-organic frameworks for efficient selective electroreduction of CO_2_ to formate. J. Mater. Chem. A.

[134] Yao D, Tang C, Vasileff A, Zhi X, Jiao Y (2021). The controllable reconstruction of Bi-MOFs for electrochemical CO_2_ reduction through electrolyte and potential mediation. Angew. Chem. Int. Ed..

[135] Zhu Q, Yang D, Liu H, Sun X, Chen C (2020). Hollow metal-organic-framework-mediated in situ architecture of copper dendrites for enhanced CO_2_ electroreduction. Angew. Chem. Int. Ed..

[136] Yao K, Wang H, Yang X, Huang Y, Kou C (2022). Metal-organic framework derived dual-metal sites for electroreduction of carbon dioxide to HCOOH. Appl. Catal. B Environ..

[137] Lamagni P, Miola M, Catalano J, Hvid MS, Mamakhel MAH (2020). Restructuring metal-organic frameworks to nanoscale bismuth electrocatalysts for highly active and selective CO_2_ reduction to formate. Adv. Funct. Mater..

[138] Duan YX, Liu KH, Zhang Q, Yan JM, Jiang Q (2020). Efficient CO_2_ reduction to HCOOH with high selectivity and energy efficiency over Bi/rGO catalyst. Small Methods.

[139] Fu HQ, Liu J, Bedford NM, Wang Y, Wright J (2022). Operando converting BiOCl into Bi_2_O_2_(CO_3_)_x_Cl_y_ for efficient electrocatalytic reduction of carbon dioxide to formate. Nano-Micro Lett..

[140] Wu D, Feng R, Xu C, Sui P-F, Zhang J (2021). Regulating the electron localization of metallic bismuth for boosting CO_2_ electroreduction. Nano-Micro Lett..

[141] Qi M, Park J, Landon RS, Kim J, Liu Y (2022). Continuous and flexible renewable-power-to-methane via liquid CO_2_ energy storage: revisiting the techno-economic potential. Renew. Sust. Energy Rev..

[142] Liu X, Yang H, He J, Liu H, Song L (2018). Highly active, durable ultrathin MoTe_2_ layers for the electroreduction of CO_2_ to CH_4_. Small.

[143] Li X, Sun Y, Xu J, Shao Y, Wu J (2019). Selective visible-light-driven photocatalytic CO_2_ reduction to CH_4_ mediated by atomically thin CuIn_5_S_8_ layers. Nat. Energy.

[144] Kim MK, Kim HJ, Lim H, Kwon Y, Jeong HM (2019). Metal-organic framework-mediated strategy for enhanced methane production on copper nanoparticles in electrochemical CO_2_ reduction. Electrochim. Acta.

[145] Yang F, Chen A, Deng PL, Zhou Y, Shahid Z (2019). Highly efficient electroconversion of carbon dioxide into hydrocarbons by cathodized copper-organic frameworks. Chem. Sci..

[146] Tan X, Yu C, Zhao C, Huang H, Yao X (2019). Restructuring of Cu_2_O to Cu_2_O@Cu-metal-organic frameworks for selective electrochemical reduction of CO_2_. ACS Appl. Mater. Interfaces.

[147] Yi JD, Xie R, Xie ZL, Chai GL, Liu TF (2020). Highly selective CO_2_ electroreduction to CH_4_ by in situ generated Cu_2_O single-type sites on a conductive MOF: Stabilizing key intermediates with hydrogen bonding. Angew. Chem. Int. Ed..

[148] Zhu H-L, Huang J-R, Zhang X-W, Wang C, Huang N-Y (2021). Highly efficient electroconversion of CO_2_ into CH_4_ by a metal-organic framework with trigonal pyramidal Cu(1)N_3_ active sites. ACS Catal..

[149] Zhang L, Li XX, Lang ZL, Liu Y, Liu J (2021). Enhanced cuprophilic interactions in crystalline catalysts facilitate the highly selective electroreduction of CO_2_ to CH_4_. J. Am. Chem. Soc..

[150] Zhang Y, Dong LZ, Li S, Huang X, Chang JN (2021). Coordination environment dependent selectivity of single-site-Cu enriched crystalline porous catalysts in CO_2_ reduction to CH_4_. Nat. Commun..

[151] Liu Y, Li S, Dai L, Li J, Lv J (2021). The synthesis of hexaazatrinaphthylene-based 2d conjugated copper metal-organic framework for highly selective and stable electroreduction of CO_2_ to methane. Angew. Chem. Int. Ed..

[152] Zhang Y, Zhou Q, Qiu ZF, Zhang XY, Chen JQ (2022). Tailoring coordination microenvironment of Cu(1) in metal-organic frameworks for enhancing electroreduction of CO_2_ to CH_4_. Adv. Funct. Mater..

[153] Fang Y, Flake JC (2017). Electrochemical reduction of CO_2_ at functionalized Au electrodes. J. Am. Chem. Soc..

[154] Xie MS, Xia BY, Li Y, Yan Y, Yang Y (2016). Amino acid modified copper electrodes for the enhanced selective electroreduction of carbon dioxide towards hydrocarbons. Energy Environ. Sci..

[155] Ahn S, Klyukin K, Wakeham RJ, Rudd JA, Lewis AR (2018). Poly-amide modified copper foam electrodes for enhanced electrochemical reduction of carbon dioxide. ACS Catal..

[156] Qiu Y, Zhong H, Xu W, Zhang T, Li X (2019). Tuning the electrocatalytic properties of a Cu electrode with organic additives containing amine group for CO_2_ reduction. J. Mater. Chem. A.

[157] Firet NJ, Smith WA (2016). Probing the reaction mechanism of CO_2_ electroreduction over ag films via operando infrared spectroscopy. ACS Catal..

[158] Perez-Gallent E, Figueiredo MC, Calle-Vallejo F, Koper MT (2017). Spectroscopic observation of a hydrogenated co dimer intermediate during co reduction on Cu(100) electrodes. Angew. Chem. Int. Ed..

[159] Zhu S, Li T, Cai W-B, Shao M (2019). CO_2_ electrochemical reduction as probed through infrared spectroscopy. ACS Energy Lett..

[160] Pei Y, Zhong H, Jin F (2021). A brief review of electrocatalytic reduction of CO_2_ materials, reaction conditions, and devices. Energy Sci. Eng..

[161] Zivkovic S, Veljkovic M (2018). Environmental impacts the of production and use of biodiesel. Environ. Sci. Pollut. Res..

[162] Bilgili L (2021). Comparative assessment of alternative marine fuels in life cycle perspective. Renew. Sust. Energy Rev..

[163] Din IU, Shaharun MS, Alotaibi MA, Alharthi AI, Naeem A (2019). Recent developments on heterogeneous catalytic CO_2_ reduction to methanol. J. CO2 Util..

[164] Zhao K, Liu Y, Quan X, Chen S, Yu H (2017). CO_2_ electroreduction at low overpotential on oxide-derived Cu/carbons fabricated from metal organic framework. ACS Appl. Mater. Interfaces.

[165] Yang X, Cheng J, Yang X, Xu Y, Sun W (2022). MOF-derived Cu@Cu_2_O heterogeneous electrocatalyst with moderate intermediates adsorption for highly selective reduction of CO_2_ to methanol. Chem. Eng. J..

[166] Payra S, Shenoy S, Chakraborty C, Tarafder K, Roy S (2020). Structure-sensitive electrocatalytic reduction of CO_2_ to methanol over carbon-supported intermetallic PtZn nano-alloys. ACS Appl. Mater. Interfaces.

[167] Yang H, Wu Y, Li G, Lin Q, Hu Q (2019). Scalable production of efficient single-atom copper decorated carbon membranes for CO_2_ electroreduction to methanol. J. Am. Chem. Soc..

[168] Liu J, Yang D, Zhou Y, Zhang G, Xing G (2021). Tricycloquinazoline-based 2D conductive metal-organic frameworks as promising electrocatalysts for CO_2_ reduction. Angew. Chem. Int. Ed..

[169] Zaza L, Rossi K, Buonsanti R (2022). Well-defined copper-based nanocatalysts for selective electrochemical reduction of CO_2_ to C_2_ products. ACS Energy Lett..

[170] Zheng Y, Vasileff A, Zhou X, Jiao Y, Jaroniec M (2019). Understanding the roadmap for electrochemical reduction of CO_2_ to multi-carbon oxygenates and hydrocarbons on copper-based catalysts. J. Am. Chem. Soc..

[171] Pei W, Zhou S, Zhao J, Xu X, Du Y (2020). Immobilized trimeric metal clusters: a family of the smallest catalysts for selective CO_2_ reduction toward multi-carbon products. Nano Energy.

[172] Woldu AR, Huang Z, Zhao P, Hu L, Astruc D (2022). Electrochemical CO_2_ reduction (CO_2_RR) to multi-carbon products over copper-based catalysts. Coordin. Chem. Rev..

[173] Zhang Y, Li K, Chen M, Wang J, Liu J (2019). Cu/Cu_2_O nanoparticles supported on vertically ZIF-l-coated nitrogen-doped graphene nanosheets for electroreduction of CO_2_ to ethanol. ACS Appl. Nano Mater..

[174] Zhao ZH, Zheng K, Huang NY, Zhu HL, Huang JR (2021). A Cu(111)@metal-organic framework as a tandem catalyst for highly selective CO_2_ electroreduction to C_2_H_4_. Chem. Commun..

[175] Han Y, Zhu S, Xu S, Niu X, Xu Z (2021). Understanding structure-activity relationship on metal-organic-framework-derived catalyst for CO_2_ electroreduction to C_2_ products. ChemElectroChem.

[176] Huo H, Wang J, Fan Q, Hu Y, Yang J (2021). Cu-MOFs derived porous Cu nanoribbons with strengthened electric field for selective CO_2_ electroreduction to C_2+_ fuels. Adv. Energy Mater..

[177] Qiu XF, Zhu HL, Huang JR, Liao PQ, Chen XM (2021). Highly selective CO_2_ electroreduction to C_2_H_4_ using a metal-organic framework with dual active sites. J. Am. Chem. Soc..

[178] Wen CF, Zhou M, Liu PF, Liu Y, Wu X (2022). Highly ethylene-selective electrocatalytic CO_2_ reduction enabled by isolated Cu-S motifs in metal-organic framework based precatalysts. Angew. Chem. Int. Ed..

[179] Xie X, Zhang X, Xie M, Xiong L, Sun H (2022). Au-activated N motifs in non-coherent cupric porphyrin metal organic frameworks for promoting and stabilizing ethylene production. Nat. Commun..

[180] Karapinar D, Huan NT, Sahraie NR, Li JK, Wakerley D (2019). Electroreduction of CO_2_ on single-site copper-nitrogen-doped carbon material: Selective formation of ethanol and reversible restructuration of the metal sites. Angew. Chem. Int. Ed..

[181] Zhao K, Nie X, Wang H, Chen S, Quan X (2020). Selective electroreduction of CO_2_ to acetone by single copper atoms anchored on N-doped porous carbon. Nat. Commun..

[182] Yang Y, Ohnoutek L, Ajmal S, Zheng X, Feng Y (2019). “Hot edges” in an inverse opal structure enable efficient CO_2_ electrochemical reduction and sensitive in situ raman characterization. J. Mater. Chem. A.

[183] Iijima G, Inomata T, Yamaguchi H, Ito M, Masuda H (2019). Role of a hydroxide layer on Cu electrodes in electrochemical CO_2_ reduction. ACS Catal..

[184] Liu Y, Jiang H, Hou Z (2021). Hidden mechanism behind the roughness-enhanced selectivity of carbon monoxide electrocatalytic reduction. Angew. Chem. Int. Ed..

[185] Dinh CT, Burdyny T, Kibria MG, Seifitokaldani A, Gabardo CM (2018). CO_2_ electroreduction to ethylene via hydroxide-mediated copper catalysis at an abrupt interface. Science.

[186] Fan L, Xia C, Zhu P, Lu Y, Wang H (2020). Electrochemical CO_2_ reduction to high-concentration pure formic acid solutions in an all-solid-state reactor. Nat. Commun..

[187] Zheng T, Zhang M, Wu L, Guo S, Liu X (2022). Upcycling CO_2_ into energy-rich long-chain compounds via electrochemical and metabolic engineering. Nat. Catal..

